# Author Correction: Multiplex bead assays enable integrated serological surveillance and reveal cross-pathogen vulnerabilities in Zambezia Province, Mozambique

**DOI:** 10.1038/s41467-025-64869-y

**Published:** 2025-10-07

**Authors:** Andrea C. Carcelen, Celso Monjane, Sophie Bérubé, Saki Takahashi, Thebora Sultane, Imelda Chelene, Gretchen Cooley, E. Brook Goodhew, Catriona Patterson, Kevin Tetteh, Manuel Mutambe, Melissa M. Higdon, George Mwinnyaa, Gilberto Nhapure, Pedro Duce, Diana L. Martin, Christopher Drakeley, William J. Moss, Ivalda Macicame

**Affiliations:** 1 Department of International Health, International Vaccine Access Center, Johns Hopkins Bloomberg School of Public Health, Baltimore, MD USA; 2 Instituto Nacional de Saúde, Maputo, Mozambique; 3 Department of Biostatistics, The University of Florida, Gainesville, FL USA; 4 Department of Epidemiology, Johns Hopkins Bloomberg School of Public Health, Baltimore, MD USA; 5 Division of Parasitic Diseases and Malaria, Centers for Disease Control and Prevention, Atlanta, GA USA; 6 Department of Infection Biology, London School of Hygiene and Tropical Medicine, London, UK; 7 Instituto Nacional de Estatística, Maputo, Mozambique

**Keywords:** Infectious diseases, Developing world

Correction to: *Nature Communications* 10.1038/s41467-025-62305-9, published online 26 August 2025

In the version of the article initially published, in the Fig. 4 colour bar header and in the first sentence of the caption, the word “log” was missing from the text now reading “Adjusted log OR” and “Adjusted log odds ratios,” respectively. The figure and caption are updated in the HTML and PDF versions of the article.

