# Supplementary material for: Multiplex bead assays enable integrated serological surveillance and reveal cross-pathogen vulnerabilities in Zambezia Province, Mozambique
Source: Nat Commun. 2025 Aug 26;16:7946. doi: 10.1038/s41467-025-62305-9 (PMC12381283; doi:10.1038/s41467-025-62305-9)
Supplement: Supplementary file 1 — Supplementary Information [file 41467_2025_62305_MOESM1_ESM.pdf]

Multiplex bead assays enable integrated serological surveillance and reveal cross-pathogen vulnerabilities in Zambezia Province Mozambique : Supplemental Material

Contents

Supplementary Methods 1
- Sample weight calculation 1
- Ethical approvals and rapid diagnostic testing criteria 1
- Quality Control and Assurance 1
- Supplementary Table 1. Determination of cutoffs for seropositivity 1
- Data analysis and modeling 2
- Calculation of wealth quintiles 2

Supplemental Results 4
- Supplementary Figure 1. MFI distributions by antigen in the COMSA samples 4
- Supplementary Figure 2. MFI distributions by antigen in the CDC Negative Control samples used to establish cutoffs for arboviruses and lymphatic filariasis antigens 5
- Supplementary Figure 3. MFI distributions by antigen in the LSHTM Negative Control samples used to establish cutoffs for malaria and SARS-CoV-2 antigens 6
- Supplementary Figure 4. Further refinement of the cutoff for Pf MSP1-19 based on the MFI distribution in the LSHTM Negative Control samples 7
- Supplementary Figure 5. ROC curves for NTD antigens 8
- Supplementary Figure 6. Seroprevalence by cluster, weighted 9
- Supplementary Figure 7. Seroprevalence by wealth quintile, weighted 10
- Supplementary Table 2. Seroprevalence adjusted for sensitivity and specificity 11
- Supplementary Table 3. Sensitivity analysis for measles IgG threshold 12
- Supplementary Figure 8. Multivariate serostatus to select antigens with potential cross-reactivity 13
- Supplementary Figure 9. Serocatalytic model fit for long-term malaria antigens 14
- Supplementary Figure 10. Pairwise Pearson's correlation of raw MFI values across antigens 15
- Supplementary Figure 11. Pearson's correlation between age and MFI value by antigen 16
- Supplementary Table 4. Seroprevalence estimates 17
  - Provincial seroprevalence 17
  - Seroprevalence by age 18
  - Seroprevalence by rural-urban cluster 19
  - Seroprevalence by sex 20
  - Seroprevalence by cluster 21
- Supplementary Table 5. Adjust odds ratios from multilevel model for seropositivity in urban vs rural clusters 31
- Supplementary Table 6. 32

Supplementary References 57

Supplementary Methods

Sample weight calculation

Sampling weights were calculated using inverse probability of selection at cluster and individual levels. Weights were then adjusted for nonresponse using the full COMSA dataset in the clusters from which individuals were selected. We ran a generalized boosting model with selection for the serosurvey as the binary outcome, and age, sex, rural vs urban designation, and individual selection weights as the covariates.[1,2] We then predicted the probability of selection using this model and took the inverse of this probability to produce a propensity-based weight. Then, we multiplied this weight by the individual selection weight and the COMSA cluster weight.[2] Finally, we trimmed these weights by setting all values that were above the median plus four times the interquartile range of the weights to that value.[3]

Ethical approvals and rapid diagnostic testing criteria

In addition to consenting for the study, participants separately provided consent for their specimens to be stored in a biorepository for up to 10 years for additional testing.

Individuals 18 months of age and older with no prior HIV diagnosis were eligible to be tested for HIV using a rapid diagnostic test (RDT). The Mozambique national testing algorithm was followed using two tests in sequence: Determine HIV-1/2 RDT and UniGold RDT. If reactive on both, participants were referred to the health facility of their choice to enroll in HIV care.

All individuals with fever in the previous 24 hours were eligible for a malaria RDT. Individuals with a reactive RDT were provided antimalarials per national guidelines. Those with signs or risk of severe malaria were referred to a health facility for follow-up care.

Quality Control and Assurance

Data were output as median fluorescence intensity (MFI). To control for background reactivity, each assay included blank wells containing Buffer B only. Samples were run in singlicate. Each plate also contained 2 negative serum controls, 6 positive serum controls, and a positive control that was serially diluted to make a 6-point curve to cover MFI signal in the linear range for most antigens. As a measure of plate-to-plate variability, the average reactivities of the antigens with the controls was used to create criteria for accepting or rejecting plate data. Plates with greater than 2 curve dilutions greater than 2 standard deviations from the mean response for more than 2 antigens were repeated. No additional normalization of data was done.

Supplementary Table 1. Determination of cutoffs for seropositivity

Cutoffs for seropositivity were determined using multiple methods due to varying availability of well-characterized controls. Below we present a flowchart for considerations made to determine the methodology.

Controls	Methodology	Pick cutoff that
Availability of international standards	Translation of values to international units	corresponds to known correlates of protection
Availability of both positive and negative controls	Receiver Operating Curve	Maximize Youden's J
If sensitivity is low	Receiver Operating Curve with floor value for sensitivity	Maximum specificity possible with sensitivity of at least 75%
If sensitivity, specificity, or seroprevalence do not seem reasonable based on other data sources (e.g. case-based surveillance or previous seroprevalence estimates)	Receiver Operating Curve	Select cutoff to match previous estimates
Availability of only negative controls	Sample mean plus 3 standard deviations	Sample mean plus 3 standard deviations on the natural scale
If controls are not normally distributed or have small number of controls	Highest negative control	

If negative controls have some high values that seem to fit a bimodal distribution	Finite mixture model (2-component model with Gaussian distributions)	Sample mean plus 3 standard deviations on the logarithmic scale
No controls available	Finite mixture model	Sample mean plus 3 standard deviations on the logarithmic scale

Data analysis and modeling

The antigen-specific seroprevalence curves by age using semi-parametric cubic regression splines assumed a binomial error distribution with logit link and individual-level sampling weights. 95% confidence intervals for age-specific seroprevalence were obtained using 1,000 bootstrap samples. We also estimated antigen-specific seroprevalence curves by age using semi-parametric cubic regression splines in the GAM, using the complementary log-log link function, which is another link commonly used for modeling seroprevalence data,[4] and found similar fits to the logit link function (results not shown).

For the catalytic models for long-term p.falciparum antigens, we omitted children under 1 year of age from the analysis to account for maternal antibodies, assumed that lambda is constant by age and time, and accounted for individual-level survey weights in the model.

The Bayesian logistic random effects models were fit using the 'brms' package in R. A separate model was fit for each antigen where the outcome was: the probability of an individual being seropositive to that antigen; the predictors were: age, the MFI response to both Glutathione-S-transferase (GST) proteins, and the MFI response to the uninfected Vero cell lysate (to control for individual-level variability in the outcomes of interest); and a random intercept was included for each cluster. Three chains were run with a burn-in of 500 iterations and 1000 posterior draws. Convergence of key parameters was monitored through trace plots and the Gelman-Rubin statistic.[5] Importantly, models for the *P. falciparum* antigens Gexp18 and Etramp5Ag1, and the *P. vivax* antigen rbp2b did not show evidence of satisfactory convergence and were excluded from further analysis.

Under the overall cluster ranking, for non-vaccine preventable pathogens, we let lower values represent clusters with lower odds of being seropositive to an antigen than the general survey population and higher values represent clusters with higher odds of being seropositive to an antigen in the general survey population. For vaccine preventable diseases (VPDs), we let lower rank values represent higher odds of being seropositive to an antigen and higher rank values represent lower odds of being seropositive to an antigen.

In terms of the individual odds of seropositivity, statistical significance was determined using an alpha level of 0.05. To account for multiple hypothesis testing, p-values were adjusted using the Benjamini and Hochberg's method.[6] The median value of significant adjusted odds ratios was 2.68 (IQR= 1.98, 5.79) suggesting a moderate level of association between responses to most pathogens, however, associations with extremely high adjusted odds ratios should be interpreted cautiously in the context of very low seroprevalence and extremely wide 95% confidence intervals (e.g. adjusted OR between P. vivax(pvdbprii) and P. vivax (pvrbp2b) = 5345 (179, 1.9x10^6)). These results are likely the result of limitations in available data and statistical modeling approaches, therefore we caution against over interpreting individual adjusted odds ratios.

Calculation of wealth quintiles

A modified version of the 2018 version of the Equity Tool (https://www.equitytool.org/) was used to calculated wealth quintiles. The tool includes questions about the presence of a refrigerator, electricity, mobile telephone, and television in the home. In addition, it includes questions about toilet facilities, cooking fuel, roof materials, wall materials, floor materials, and source of drinking water. The answer options included with the tool were expanded for the COMSA Dried Blood Spot Study to capture relevant detailed information to be able to compare household characteristics to other studies and surveys in Mozambique. Before utilizing the tool, answers to each question were collapsed into the broader categories necessary to run the tool (e.g. grouping appropriate answer options into an 'other' category).

Because the tool requires information about the presence of electricity and the COMSA DBS study did not collect information on electricity, the presence or absence of electricity for each household was predicted prior to running

the tool. To predict electricity, data collected as part of the COMSA Verbal Autopsy and Social Autopsy (VASA) study,[7] which collected the same household variables as the COMSA DBS study, was utilized. The VASA data was divided into a training dataset (66.7%) and a test dataset (33.3%). Backward elimination logistic regression was performed on the training dataset using SAS version 9.4

```
proc logistic data=train;
class household_characteristics;
model electricity(event='1') = household_characteristics /
selection=backward fast slstay=0.2 ctable pprob=0 to 1 by 0.05);
run;
```

Household characteristics included the following: water source, toilet type, type of fuel used for cooking, and the primary material making up each of the following: walls, roof, and floor. In addition, information on ownership of each of the following items was included: radio, television, refrigerator, telephone landline, mobile phone, computer, bike, motorcycle, and car/truck.

The resulting, best model for classifying electricity retained water source, cooking fuel, toilet type, wall material, and ownership of each of the following items: radio, television, mobile phone, and car, resulting in an area under the curve (AUC) of 0.9566. The probability cutoff that produced the highest classification, 0.469, was selected as the threshold for determining the presence of electricity. The final model, parameter estimates, and probability threshold generated from the training dataset were then applied to the test dataset, resulting in a sensitivity for electricity of 70.0%, a specificity of 99.4%, and a total classification accuracy of 96.6%. These results indicated acceptable performance of the model, thus, the same model, parameters and threshold were applied to the COMSA DBS study to predict the presence or absence of electricity for each household:

```
proc logistic data=train plots=roc;
class WATER_SOURCE_3 toilet_3 fuel_1 wall_2;
model elec(event='1') = car tv radio mobile water_source toilet_type cooking_fuel wall_material;
score data=dbs out=dbs_pred;
run;

data dbs_final;
set dbs_pred;
if p_1>0.469 then electricity=1;
else if 0<=p_1<=0.469 then electricity=0;
else if p_1=. then electricity=.;
run;
```

Lastly, the equity tool was run on the final dataset using the predicted electricity variable to assign each household to a wealth quintile.

Supplemental Results

Supplementary Figure 1. MFI distributions by antigen in the COMSA samples

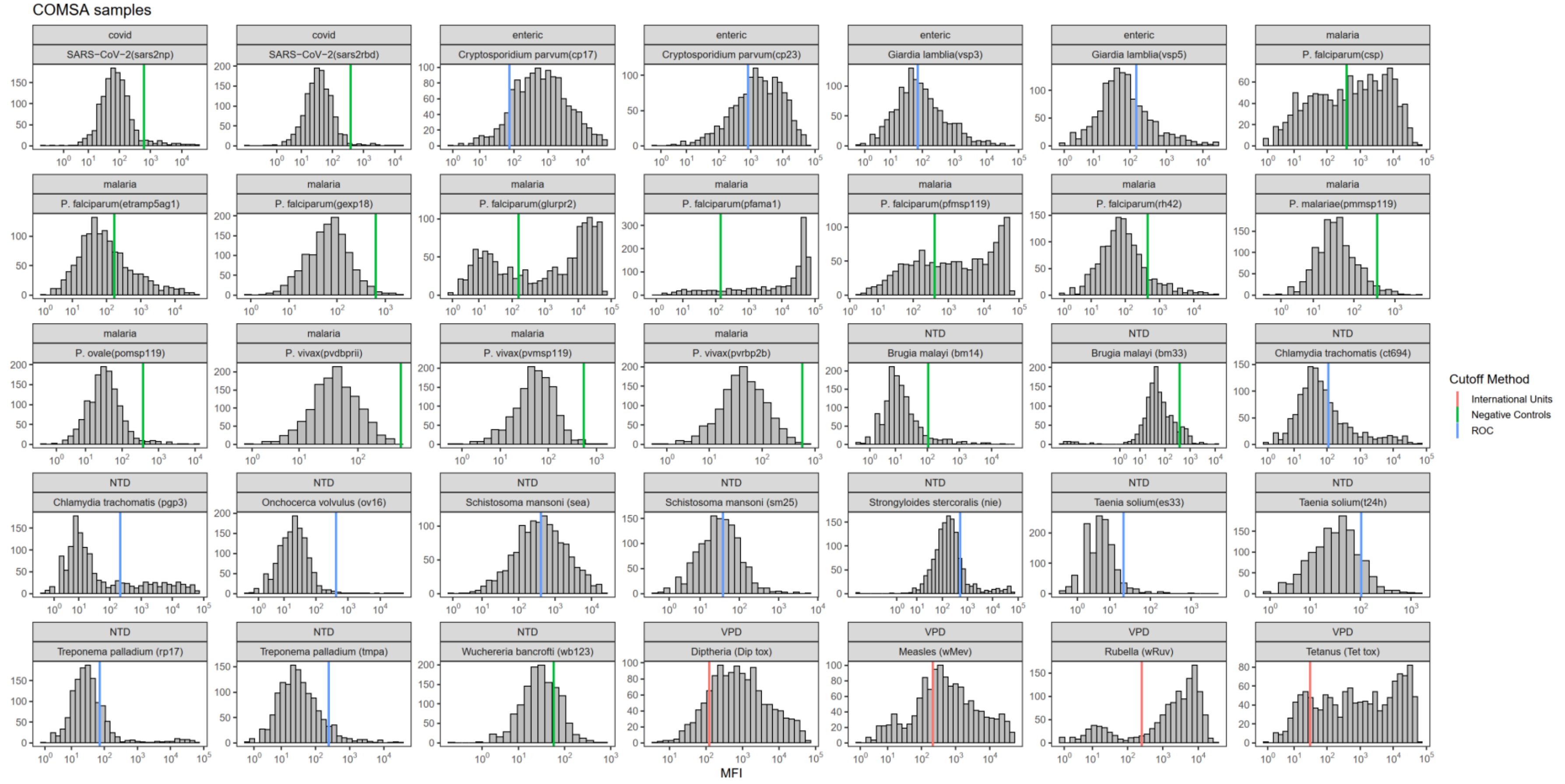

Each box corresponds to one antigen. X-axis represents the background-subtracted MFI values. Y-axis represents number of participants with that MFI value. Line represents the cutoff threshold for seropositivity. Color of the line indicates which methodology was used to determine that cutoff.

Supplementary Figure 2. MFI distributions by antigen in the CDC Negative Control samples used to establish cutoffs for arboviruses and lymphatic filariasis antigens

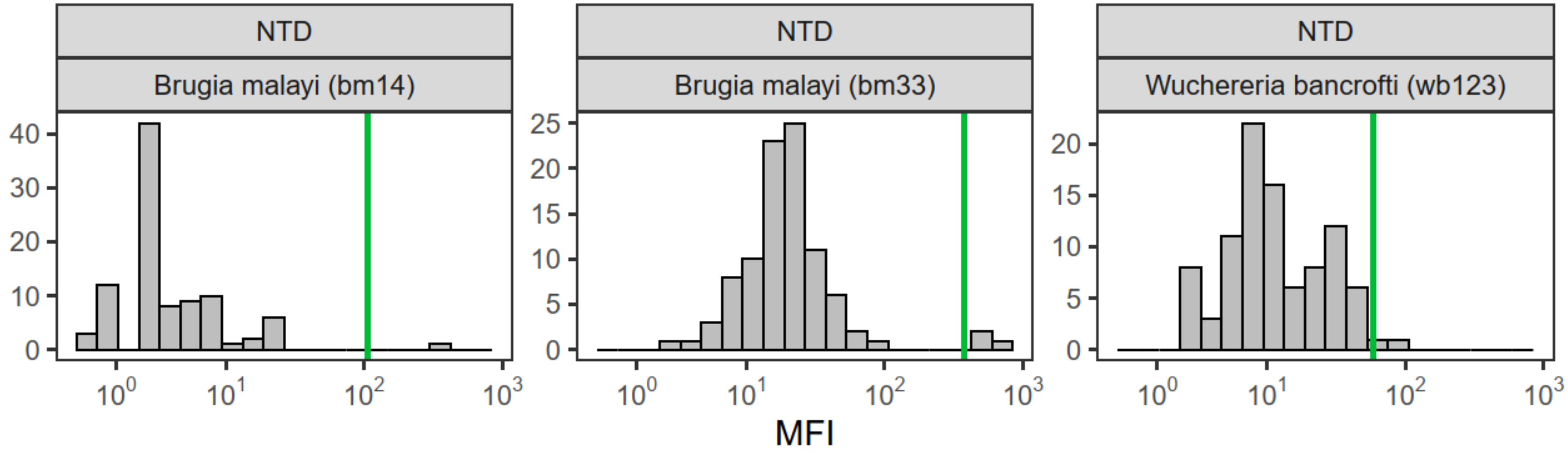

Each box corresponds to one antigen. X-axis represents the background-subtracted MFI values. Y-axis represents number of negative control samples with that MFI value. Line represents the cutoff threshold for seropositivity. Cutoffs were calculated as 3 sample standard deviations above the sample mean, on the natural scale of MFI values. Number of negative control samples was 94 for lymphatic filariasis antigens. These are samples from individuals born in US reporting no international travel when employed by CDC.

Supplementary Figure 3. MFI distributions by antigen in the LSHTM Negative Control samples used to establish cutoffs for malaria and SARS-CoV-2 antigens

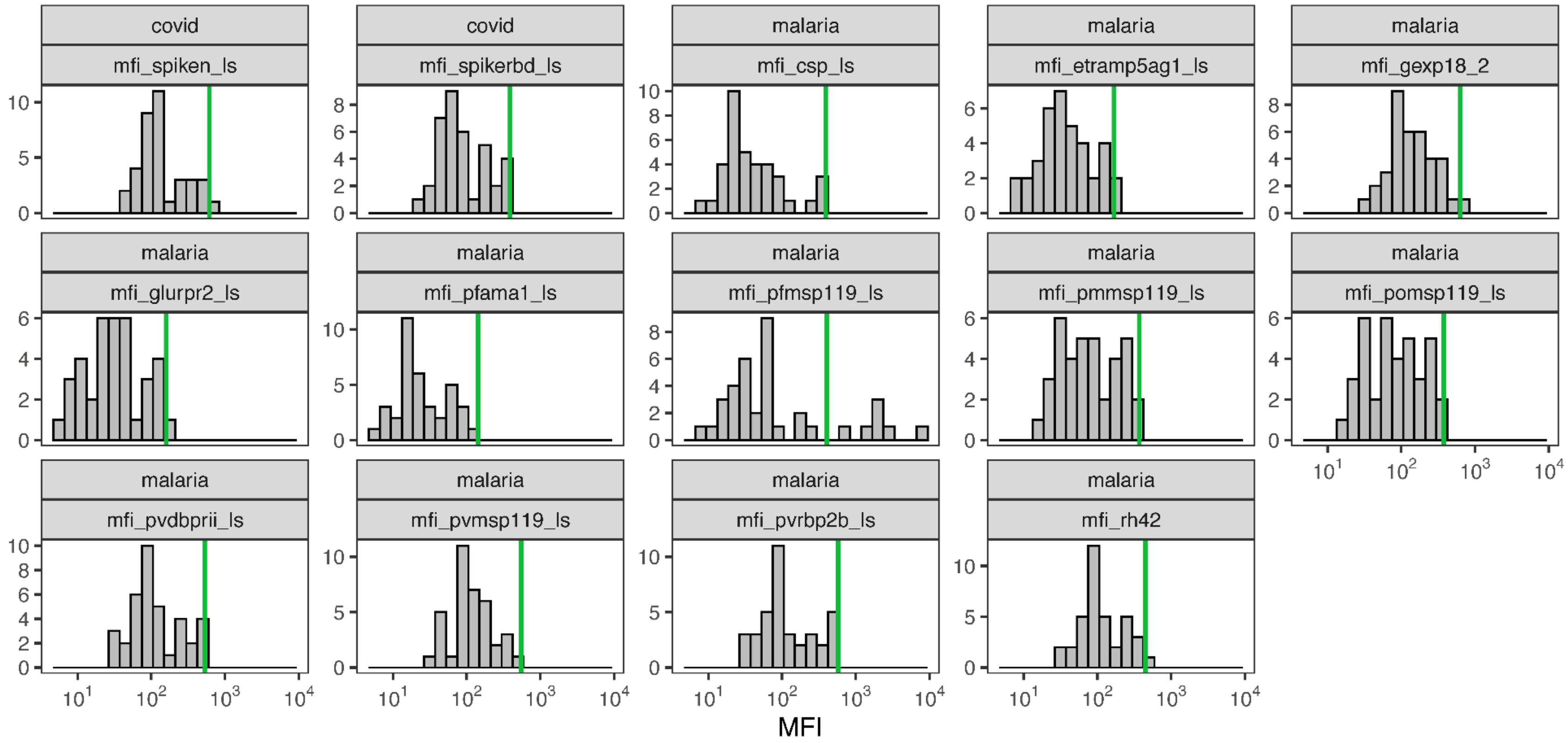

Each box corresponds to one antigen. X-axis represents the background-subtracted MFI values. Y-axis represents number of negative control samples with that MFI value. Line represents the cutoff threshold for seropositivity. Cutoffs were calculated as the maximum observed MFI value among these samples for that antigen (with the exception of Pf MSP1-19, see next page). Number of negative control samples per antigen: n=37. These are samples from blood bank donors that tested negative for malaria by a serology test.

Supplementary Figure 4. Further refinement of the cutoff for Pf MSP1-19 based on the MFI distribution in the LSHTM Negative Control samples

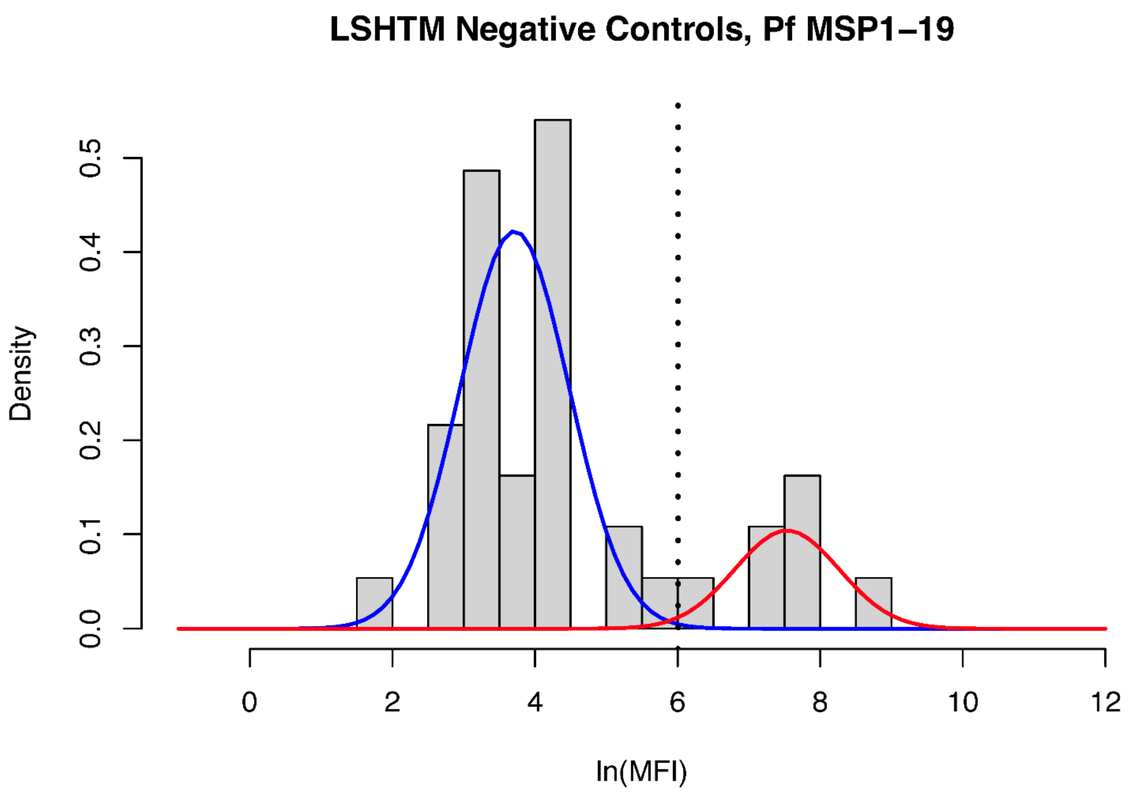

Given the bimodal distribution of background-subtracted MFI values, a two-component Gaussian finite mixture model was fit to the log MFI values of the 37 LSHTM Negative Control samples for just this antigen (seronegative distribution in blue, seropositive/cross-reactive distribution in red). The cutoff, depicted by the black dotted line, was calculated as 3 standard deviations above the mean of the seronegative distribution.

Supplementary Figure 5. ROC curves for NTD antigens

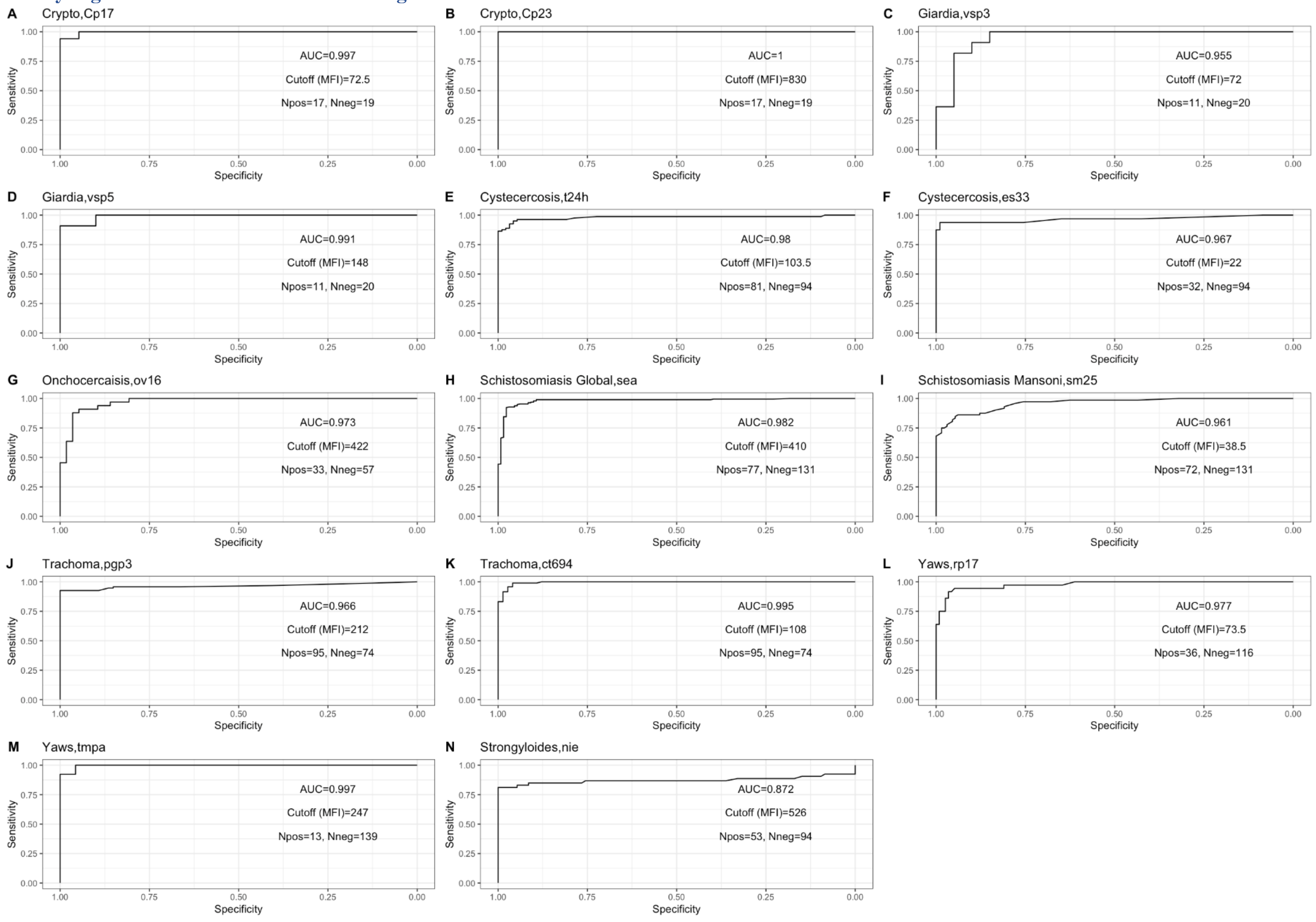

Panels (A-N) Receiver Operating Curves (ROC) for neglected tropical disease antigens that had negative and positive control panels available. Seropositivity cutoff in mean fluorescence intensity (MFI) calculated based on ROC as designated in Table 1. Number of positive and negative controls, cutoff value on the MFI scale, and AUC value are shown under each antigen.

Supplementary Figure 6. Seroprevalence by cluster, weighted

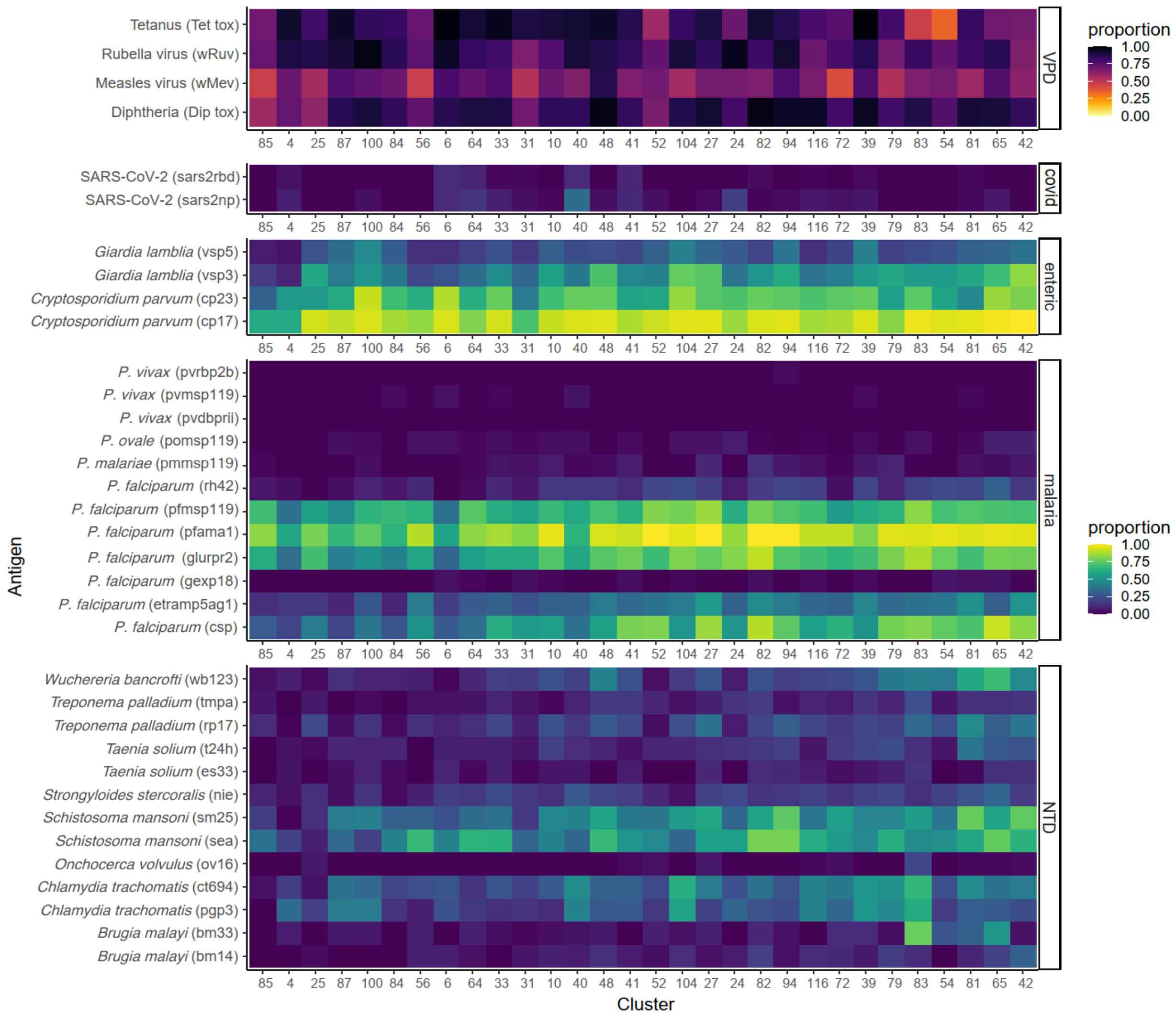

Clusters are in order of increasing seroprevalence overall, so the clusters on the right tend to have higher seroprevalence. Vaccine preventable diseases are in a different color scheme to represent that lower seroprevalence demonstrates more vulnerability. This is like higher seroprevalence representing more vulnerability to infectious diseases for all other antigens.

Supplementary Figure 7. Seroprevalence by wealth quintile, weighted

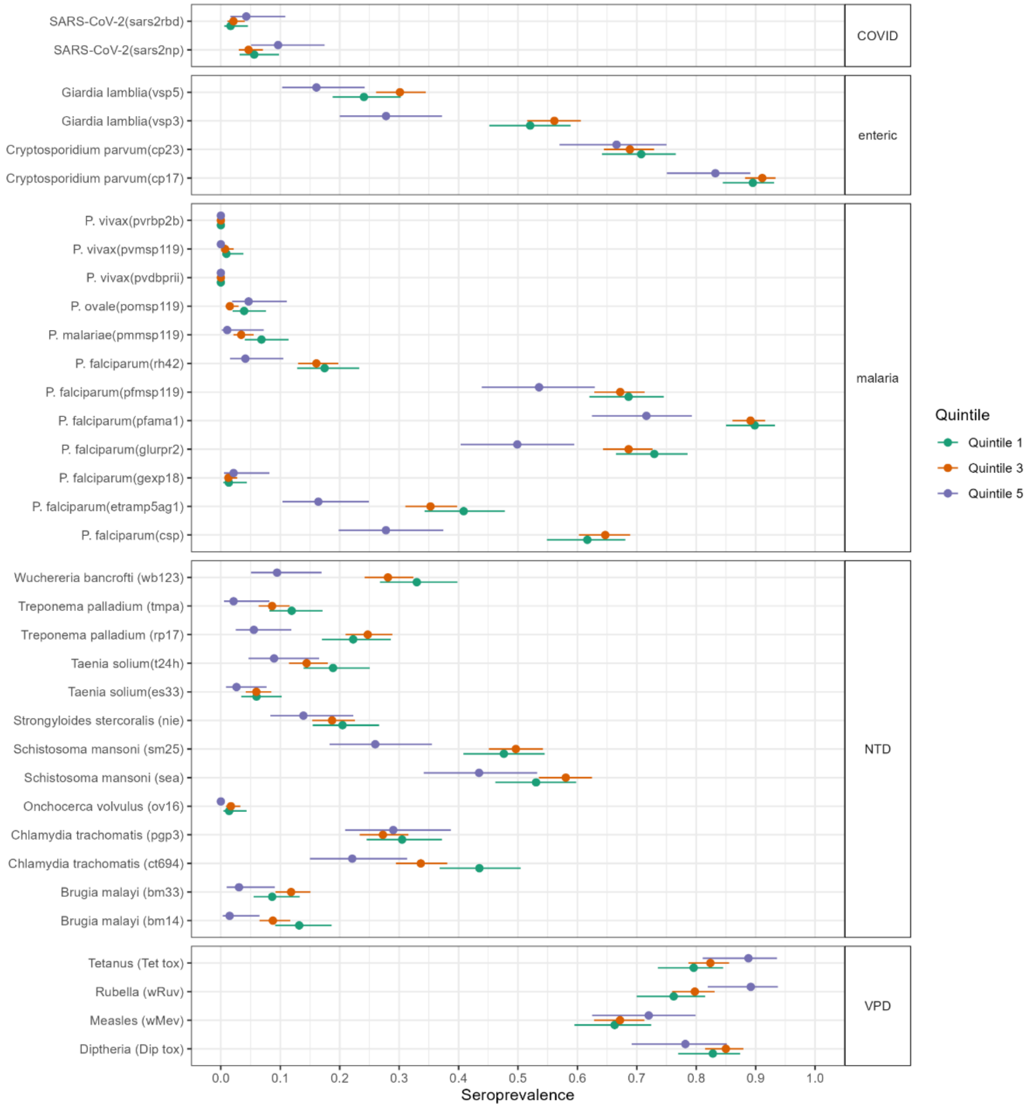

Weighted provincial seroprevalence for each wealth quintile is depicted by the dots, and 95% confidence intervals are represented by the lines for each antigen. All five wealth quintiles were calculated, but only 3 are represented here. Quintile 1 (green) is the poorest, and quintile 5 (purple) is the wealthiest.

Supplementary Table 2. Seroprevalence adjusted for sensitivity and specificity

Antigen type	Pathogen	Antigen	Sensitivity	Specificity	Seroprevalence	LCI	UCI	Adjusted seroprevalence	Adjusted LCI	Adjusted UCI
**NTD**	*Chlamydia trachomatis*	ct694	96%	97%	34%	31%	36%	33%	31%	36%
**NTD**	*Chlamydia trachomatis*	pgp3	93%	100%	28%	25%	30%	30%	27%	33%
**NTD**	*Onchocerca volvulus*	ov16	91%	95%	2%	1%	2%	0%	0%	0%
**NTD**	*Schistosoma mansoni*	sm25	75%	98%	46%	43%	49%	60%	57%	64%
**NTD**	*Schistosoma spp.*	sea	85%	98%	52%	50%	55%	61%	58%	65%
**NTD**	*Schistosoma spp.*	sea	89%	98%	52%	50%	55%	58%	55%	61%
**NTD**	*Schistosoma spp.*	sea	77%	98%	52%	50%	55%	68%	64%	71%
**NTD**	*Schistosoma spp.*	sea	91%	98%	52%	50%	55%	57%	54%	60%
**NTD**	*Strongyloides stercoralis*	nie	81%	100%	17%	15%	20%	21%	19%	24%
**NTD**	*Taenia solium*	es33	88%	100%	6%	5%	8%	7%	6%	9%
**NTD**	*Taenia solium*	t24h	86%	100%	14%	12%	16%	16%	14%	18%
**NTD**	*Treponema pallidum*	rp17	94%	95%	21%	19%	24%	18%	15%	20%
**NTD**	*Treponema pallidum*	tmpa	100%	96%	9%	7%	11%	5%	3%	6%
**enteric**	*Cryptosporidium parvum*	cp17	100%	95%	90%	88%	91%	89%	88%	91%
**enteric**	*Cryptosporidium parvum*	cp23	100%	100%	69%	66%	71%	69%	66%	71%
**enteric**	*Giardia lamblia*	vsp3	100%	85%	50%	47%	53%	41%	38%	45%
**enteric**	*Giardia lamblia*	vsp5	91%	100%	26%	24%	29%	29%	26%	32%

Provincial level weighted seroprevalence results were adjusted for sensitivity and specificity of the assay for the antigens where had known positive and negative controls.

Supplementary Table 3. Sensitivity analysis for measles IgG threshold

Although the cut-off used in this study was the previously validated 153mIU/mL , we recognize that 120mIU/mL is often considered the threshold. We adjusted the threshold down and found seropositivity increased to 71.2%. Seroprevalence patterns by age and other participant characteristics remained the similar.

Cutoffs	153 mIU/mL Seroprevalence (95% CI)	120 mIU/mL Seroprevalence (95% CI)
Overall	66.6 (63.9, 69.2)	71.2 (68.6, 73.7)
<5	66.0 (61.0, 70.6)	69.3 (64.4, 73.8)
5-17	62.2 (57.6, 66.7)	67.9 (63.3, 72.1)
18+	71.7 (67.2, 75.9)	75.9 (71.5, 79.8)
Male	62.9 (58.9, 66.8)	68.9 (64.9, 72.5)
Female	69.9 (66.2, 73.3)	73.2 (69.7, 76.5)
Urban	71.0 (63.5, 63.0)	75.6 (68.3, 81.6)
Rural	65.9 (63.0, 68.8)	70.5 (67.6, 73.2)

Supplementary Figure 8. Multivariate serostatus to select antigens with potential cross-reactivity

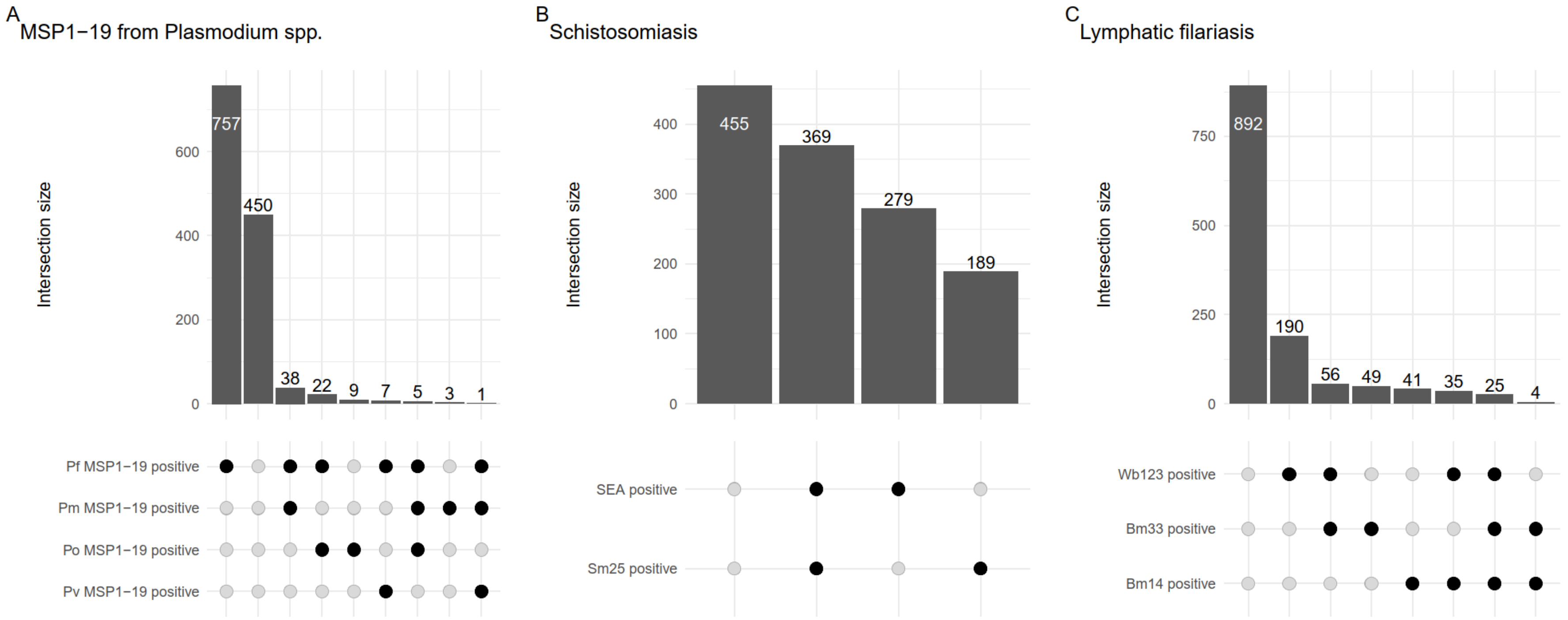

(A) homologous MSP1-19 antigens from the 4 *Plasmodium spp.* included on this panel; (B) SEA (pan-*Schistosoma spp.* marker) vs. Sm25 (*Schistosoma mansoni*-specific marker) for Schistosomiasis, and (C) Wb123 (*Wuchereria bancrofti*, which occurs in Africa) vs. Bm33 and Bm14 (*Brugia malayi*, which does not occur in Africa), which are known to cross-react, for Lymphatic filariasis.

Supplementary Figure 9. Serocatalytic model fit for long-term malaria antigens

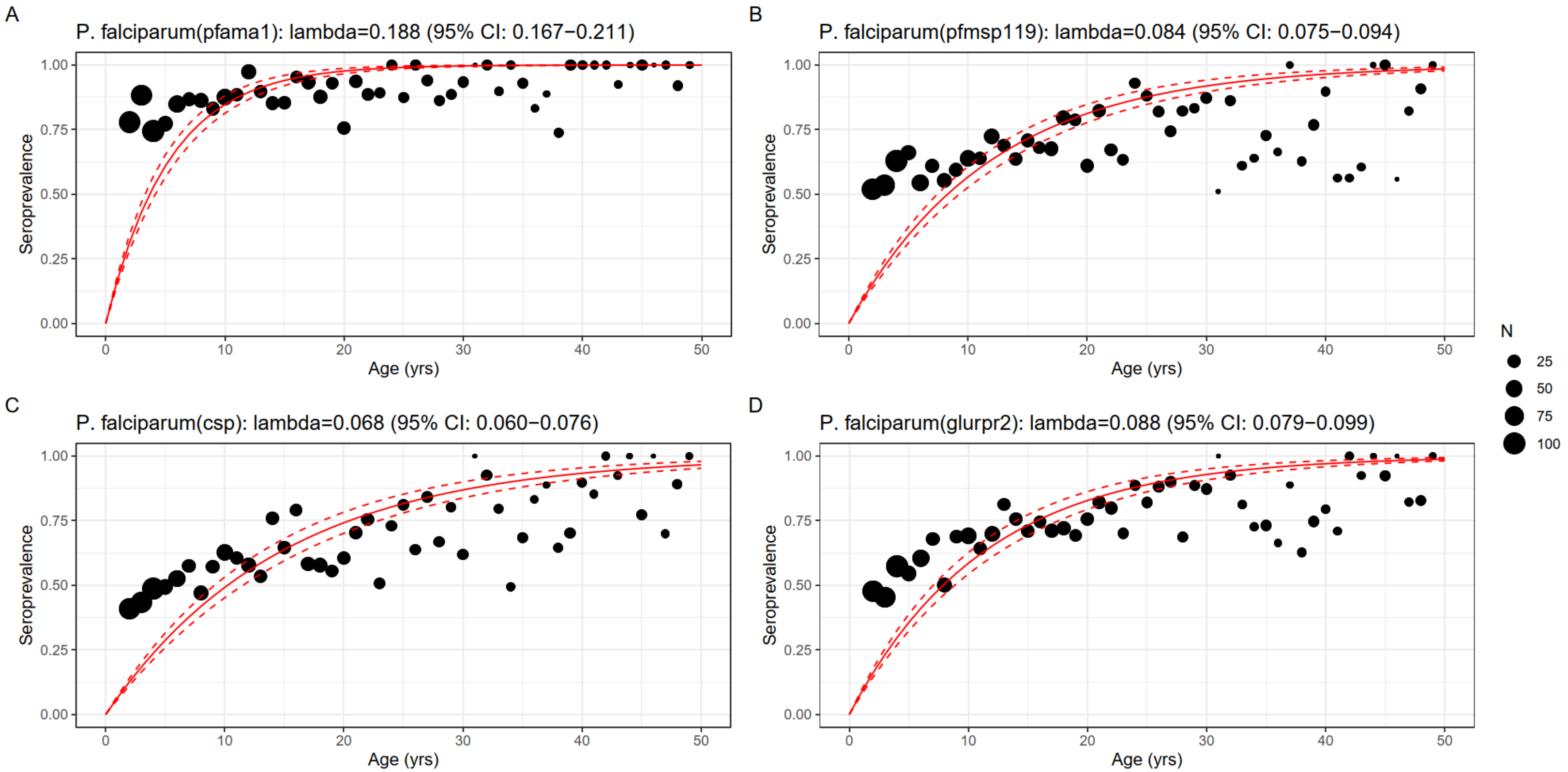

Seroprevalence (y-axis) by year of age (x-axis), with observed shown in black circles with size proportional to number of samples, for **(A)** Pf AMA-1, **(B)** Pf MSP1-19, **(C)** Pf CSP, and **(D)** GLURP-R2. Point estimate and 95% CI of the model fit is shown in red. This simple model tended to under-estimate seroprevalence in younger ages (<10 years) and over-estimate seroprevalence in adults, suggesting that more complex models that allow for variation in lambda by age or time may improve overall model fit.

Supplementary Figure 10. Pairwise Pearson's correlation of raw MFI values across antigens

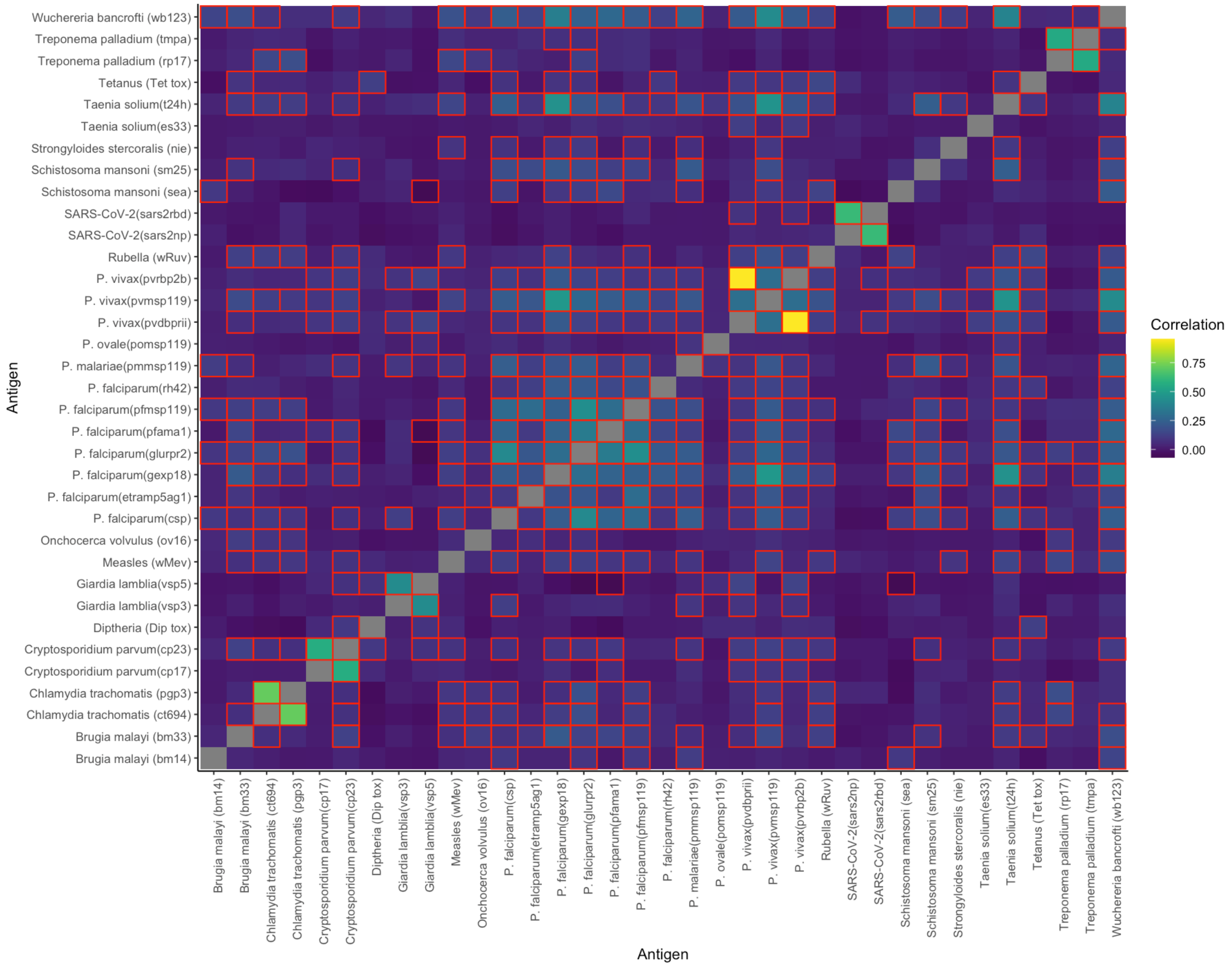

Values that are statistically significantly different from 0 after adjusting for multiple hypothesis tests using Benjamini and Hoshberg's method are in red.

Supplementary Figure 11. Pearson's correlation between age and MFI value by antigen

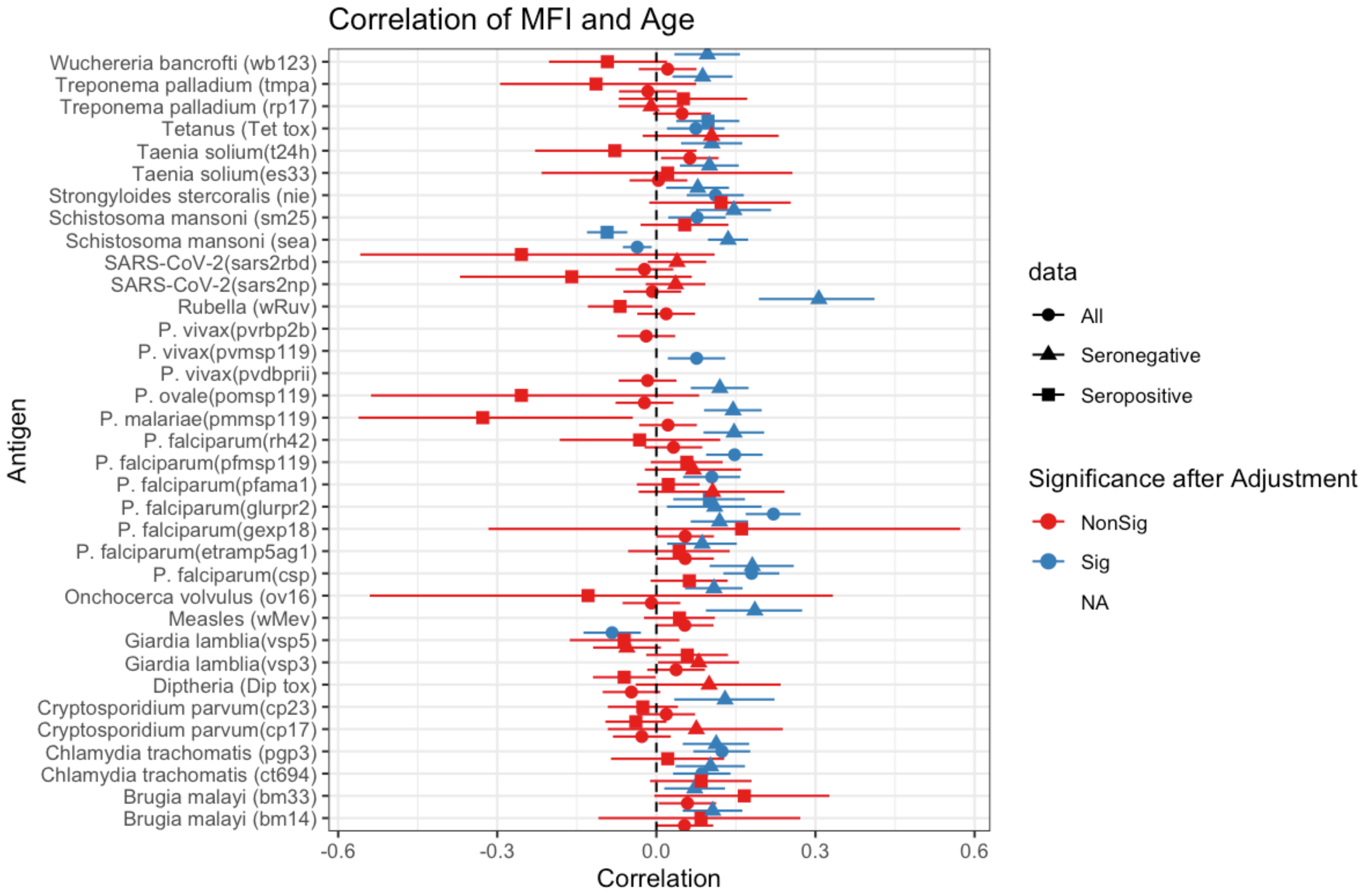

Correlations between raw MFI values and age in years is noted on the x-axis for each antigen (y-axis). The dot represents correlation when all individuals are included, triangle among only seronegative individuals and square shape among only seropositive individuals. The lines represent 95% confidence intervals around the correlation estimates. All correlations were subjected to a hypothesis test with a null value of 0, significance was determined based on a p-value of 0.05 after multiple hypotheses testing correction (Benjamini and Hochberg) was applied. Significant values are depicted in blue, and non-significant values in red.

Supplementary Table 4. Seroprevalence estimates

Provincial seroprevalence

Antigen	proportion	LCI	UCI
SARS-CoV-2 (sars2np)	0.06	0.05	0.08
SARS-CoV-2 (sars2rbd)	0.03	0.02	0.04
Cryptosporidium parvum (cp17)	0.90	0.88	0.91
Cryptosporidium parvum (cp23)	0.69	0.66	0.71
Giardia lamblia (vsp3)	0.50	0.47	0.53
Giardia lamblia (vsp5)	0.26	0.24	0.29
P. falciparum (csp)	0.59	0.56	0.62
P. falciparum (etramp5ag1)	0.34	0.31	0.37
P. falciparum (gexp18)	0.01	0.01	0.02
P. falciparum (glurpr2)	0.67	0.64	0.69
P. falciparum (pfama1)	0.87	0.85	0.89
P. falciparum (pfmsp119)	0.66	0.64	0.69
P. falciparum (rh42)	0.14	0.12	0.16
P. malariae (pmmsp119)	0.04	0.03	0.05
P. ovale (pomsp119)	0.03	0.02	0.04
P. vivax (pvdbprii)	0.00	0.00	0.00
P. vivax (pvmsp119)	0.01	0.00	0.01
P. vivax (pvrbp2b)	0.00	0.00	0.01
Brugia malayi (bm14)	0.08	0.07	0.10
Brugia malayi (bm33)	0.10	0.09	0.12
Chlamydia trachomatis (pgp3)	0.34	0.31	0.36
Chlamydia trachomatis (ct694)	0.28	0.25	0.30
Onchocerca volvulus (ov16)	0.02	0.01	0.02
Schistosoma mansoni (sea)	0.52	0.50	0.55
Schistosoma mansoni (sm25)	0.46	0.43	0.49
Strongyloides stercoralis (nie)	0.17	0.15	0.20
Taenia solium (es33)	0.06	0.05	0.08
Taenia solium (t24h)	0.14	0.12	0.16
Treponema palladium (rp17)	0.21	0.19	0.24
Treponema palladium (tmpa)	0.09	0.07	0.11
Wuchereria bancrofti (wb123)	0.25	0.22	0.27
Diphtheria (Dip tox)	0.85	0.83	0.87
Measles virus (wMev)	0.67	0.64	0.69
Rubella virus (wRuv)	0.81	0.79	0.83
Tetanus (Tet tox)	0.83	0.81	0.85

Seroprevalence by age

Antigen	6months-4years proportion	LCI	UCI	5-17 years proportion	LCI	UCI	18-49 years proportion	LCI	UCI
SARS-CoV-2 (sars2np)	0.05	0.03	0.07	0.07	0.05	0.10	0.07	0.05	0.10
SARS-CoV-2 (sars2rbd)	0.03	0.01	0.05	0.02	0.01	0.04	0.03	0.02	0.05
Cryptosporidium parvum (cp17)	0.89	0.86	0.92	0.89	0.86	0.92	0.91	0.88	0.94
Cryptosporidium parvum (cp23)	0.66	0.61	0.70	0.69	0.64	0.73	0.71	0.67	0.75
Giardia lamblia (vsp3)	0.56	0.51	0.61	0.49	0.45	0.54	0.47	0.42	0.52
Giardia lamblia (vsp5)	0.41	0.36	0.46	0.23	0.19	0.27	0.19	0.15	0.23
P. falciparum (csp)	0.41	0.36	0.46	0.59	0.54	0.63	0.72	0.68	0.76
P. falciparum (etramp5ag1)	0.30	0.25	0.35	0.34	0.30	0.39	0.36	0.32	0.41
P. falciparum (gexp18)	0.01	0.00	0.02	0.02	0.01	0.04	0.01	0.00	0.02
P. falciparum (glurpr2)	0.48	0.43	0.53	0.66	0.62	0.71	0.80	0.76	0.84
P. falciparum (pfama1)	0.79	0.75	0.83	0.87	0.84	0.90	0.92	0.89	0.94
P. falciparum (pfmsp119)	0.55	0.50	0.60	0.64	0.59	0.68	0.77	0.73	0.81
P. falciparum (rh42)	0.10	0.08	0.14	0.14	0.11	0.18	0.15	0.12	0.18
P. malariae (pmmsp119)	0.04	0.03	0.07	0.03	0.02	0.06	0.04	0.03	0.07
P. ovale (pomsp119)	0.03	0.02	0.06	0.03	0.01	0.05	0.02	0.01	0.05
P. vivax (pvdbprii)	0.00	0.00	0.00	0.00	0.00	0.00	0.00	0.00	0.00
P. vivax (pvmsp119)	0.00	0.00	0.02	0.01	0.00	0.03	0.01	0.00	0.02
P. vivax (pvrbp2b)	0.00	0.00	0.00	0.00	0.00	0.00	0.00	0.00	0.02
Brugia malayi (bm14)	0.05	0.03	0.08	0.08	0.06	0.11	0.11	0.09	0.15
Brugia malayi (bm33)	0.07	0.05	0.10	0.12	0.09	0.16	0.10	0.08	0.13
Chlamydia trachomatis (pgp3)	0.27	0.22	0.32	0.33	0.29	0.38	0.39	0.34	0.44
Chlamydia trachomatis (ct694)	0.17	0.14	0.22	0.24	0.20	0.28	0.39	0.35	0.44
Onchocerca volvulus (ov16)	0.02	0.01	0.04	0.01	0.00	0.03	0.02	0.01	0.03
Schistosoma mansoni (sea)	0.40	0.35	0.45	0.61	0.56	0.65	0.53	0.48	0.57
Schistosoma mansoni (sm25)	0.35	0.30	0.40	0.47	0.42	0.51	0.53	0.48	0.58
Strongyloides stercoralis (nie)	0.11	0.08	0.15	0.15	0.12	0.19	0.24	0.20	0.28
Taenia solium (es33)	0.05	0.04	0.08	0.08	0.06	0.11	0.05	0.03	0.07
Taenia solium (t24h)	0.10	0.08	0.14	0.14	0.11	0.18	0.16	0.12	0.20
Treponema palladium (rp17)	0.18	0.14	0.22	0.20	0.17	0.24	0.24	0.20	0.29
Treponema palladium (tmpa)	0.07	0.04	0.10	0.10	0.07	0.13	0.09	0.07	0.13
Wuchereria bancrofti (wb123)	0.19	0.16	0.24	0.28	0.24	0.32	0.25	0.21	0.30
Diphtheria (Dip tox)	0.84	0.80	0.88	0.84	0.81	0.87	0.86	0.82	0.89
Measles virus (wMev)	0.66	0.61	0.71	0.62	0.58	0.67	0.72	0.67	0.76
Rubella virus (wRuv)	0.73	0.68	0.77	0.82	0.78	0.85	0.87	0.83	0.90
Tetanus (Tet tox)	0.86	0.82	0.89	0.82	0.78	0.85	0.83	0.79	0.86

Seroprevalence by rural-urban cluster

Antigen	Rural proportion	LCI	UCI	Urban proportion	LCI	UCI
SARS-CoV-2 (sars2np)	0.05	0.04	0.07	0.12	0.08	0.18
SARS-CoV-2 (sars2rbd)	0.02	0.01	0.03	0.07	0.04	0.13
Cryptosporidium parvum (cp17)	0.91	0.89	0.93	0.82	0.75	0.87
Cryptosporidium parvum (cp23)	0.69	0.67	0.72	0.65	0.58	0.72
Giardia lamblia (vsp3)	0.53	0.50	0.56	0.32	0.26	0.40
Giardia lamblia (vsp5)	0.28	0.25	0.30	0.17	0.12	0.24
P. falciparum (csp)	0.63	0.60	0.66	0.34	0.27	0.42
P. falciparum (etramp5ag1)	0.36	0.33	0.39	0.23	0.17	0.30
P. falciparum (gexp18)	0.01	0.01	0.02	0.01	0.00	0.05
P. falciparum (glurpr2)	0.69	0.66	0.72	0.53	0.45	0.60
P. falciparum (pfama1)	0.89	0.87	0.90	0.75	0.68	0.81
P. falciparum (pfmsp119)	0.67	0.65	0.70	0.59	0.51	0.66
P. falciparum (rh42)	0.15	0.13	0.18	0.02	0.01	0.06
P. malariae (pmmsp119)	0.04	0.03	0.06	0.01	0.00	0.05
P. ovale (pomsp119)	0.03	0.02	0.04	0.02	0.01	0.06
P. vivax (pvdbprii)	0.00	0.00	0.00	0.00	0.00	0.00
P. vivax (pvmsp119)	0.01	0.00	0.01	0.01	0.00	0.05
P. vivax (pvrbp2b)	0.00	0.00	0.01	0.00	0.00	0.00
Brugia malayi (bm14)	0.09	0.08	0.11	0.04	0.02	0.09
Brugia malayi (bm33)	0.11	0.09	0.13	0.05	0.03	0.10
Chlamydia trachomatis (pgp3)	0.36	0.33	0.39	0.17	0.12	0.24
Chlamydia trachomatis (ct694)	0.29	0.26	0.31	0.22	0.16	0.30
Onchocerca volvulus (ov16)	0.02	0.01	0.03	0.01	0.00	0.05
Schistosoma mansoni (sea)	0.53	0.50	0.56	0.47	0.40	0.55
Schistosoma mansoni (sm25)	0.49	0.46	0.52	0.25	0.19	0.33
Strongyloides stercoralis (nie)	0.18	0.15	0.20	0.15	0.10	0.21
Taenia solium (es33)	0.07	0.05	0.08	0.03	0.01	0.07
Taenia solium (t24h)	0.15	0.13	0.17	0.08	0.04	0.13
Treponema palladium (rp17)	0.23	0.20	0.25	0.11	0.07	0.17
Treponema palladium (tmpa)	0.09	0.08	0.11	0.05	0.02	0.10
Wuchereria bancrofti (wb123)	0.27	0.25	0.30	0.07	0.04	0.13
Diphtheria (Dip tox)	0.85	0.83	0.87	0.82	0.75	0.87
Measles virus (wMev)	0.66	0.63	0.69	0.71	0.63	0.77
Rubella virus (wRuv)	0.80	0.78	0.83	0.87	0.81	0.91
Tetanus (Tet tox)	0.82	0.80	0.85	0.89	0.83	0.93

Seroprevalence by sex

Antigen	Male proportion	LCI	UCI	Female proportion	LCI	UCI
SARS-CoV-2 (sars2np)	0.07	0.06	0.10	0.05	0.04	0.08
SARS-CoV-2 (sars2rbd)	0.03	0.02	0.05	0.02	0.01	0.03
Cryptosporidium parvum (cp17)	0.89	0.87	0.92	0.90	0.88	0.92
Cryptosporidium parvum (cp23)	0.66	0.62	0.70	0.71	0.68	0.75
Giardia lamblia (vsp3)	0.48	0.44	0.52	0.52	0.48	0.56
Giardia lamblia (vsp5)	0.25	0.21	0.28	0.28	0.24	0.31
P. falciparum (csp)	0.58	0.54	0.62	0.60	0.56	0.64
P. falciparum (etramp5ag1)	0.34	0.31	0.38	0.34	0.30	0.37
P. falciparum (gexp18)	0.01	0.01	0.02	0.01	0.01	0.03
P. falciparum (glurpr2)	0.67	0.63	0.71	0.66	0.62	0.70
P. falciparum (pfama1)	0.85	0.82	0.88	0.88	0.86	0.90
P. falciparum (pfmsp119)	0.62	0.58	0.66	0.70	0.66	0.73
P. falciparum (rh42)	0.12	0.10	0.15	0.14	0.12	0.17
P. malariae (pmmsp119)	0.04	0.03	0.06	0.03	0.02	0.05
P. ovale (pomsp119)	0.02	0.01	0.03	0.04	0.02	0.05
P. vivax (pvdbprii)	0.00	0.00	0.00	0.00	0.00	0.00
P. vivax (pvmsp119)	0.01	0.00	0.02	0.01	0.00	0.02
P. vivax (pvrbp2b)	0.00	0.00	0.00	0.00	0.00	0.01
Brugia malayi (bm14)	0.10	0.08	0.13	0.07	0.05	0.09
Brugia malayi (bm33)	0.10	0.08	0.13	0.10	0.08	0.13
Chlamydia trachomatis (pgp3)	0.22	0.19	0.26	0.32	0.29	0.36
Chlamydia trachomatis (ct694)	0.31	0.27	0.35	0.36	0.33	0.40
Onchocerca volvulus (ov16)	0.02	0.01	0.03	0.01	0.01	0.02
Schistosoma mansoni (sea)	0.52	0.48	0.56	0.53	0.49	0.57
Schistosoma mansoni (sm25)	0.44	0.40	0.49	0.47	0.43	0.51
Strongyloides stercoralis (nie)	0.18	0.15	0.21	0.17	0.14	0.20
Taenia solium (es33)	0.05	0.03	0.07	0.07	0.05	0.10
Taenia solium (t24h)	0.11	0.09	0.14	0.16	0.13	0.19
Treponema palladium (rp17)	0.21	0.18	0.25	0.21	0.18	0.25
Treponema palladium (tmpa)	0.07	0.05	0.10	0.10	0.08	0.13
Wuchereria bancrofti (wb123)	0.24	0.21	0.28	0.25	0.22	0.28
Diphtheria (Dip tox)	0.85	0.82	0.88	0.85	0.82	0.87
Measles virus (wMev)	0.63	0.59	0.67	0.70	0.66	0.73
Rubella virus (wRuv)	0.81	0.77	0.84	0.82	0.79	0.85
Tetanus (Tet tox)	0.81	0.78	0.84	0.85	0.82	0.88

Seroprevalence by cluster

Antigen	Cluster Number	proportion	LCI	UCI	Cluster Number	proportion	LCI	UCI	Cluster Number	proportion	LCI	UCI
SARS-CoV-2 (sars2np)	10	0.02	0.00	0.11	100	0.03	0.00	0.19	104	0.00	0.00	0.00
SARS-CoV-2 (sars2rbd)	10	0.00	0.00	0.00	100	0.00	0.00	0.00	104	0.00	0.00	0.00
Cryptosporidium parvum (cp17)	10	0.91	0.81	0.96	100	0.95	0.80	0.99	104	0.95	0.73	0.99
Cryptosporidium parvum (cp23)	10	0.69	0.56	0.80	100	0.91	0.76	0.97	104	0.84	0.64	0.94
Giardia lamblia (vsp3)	10	0.57	0.44	0.69	100	0.53	0.36	0.68	104	0.76	0.56	0.89
Giardia lamblia (vsp5)	10	0.29	0.19	0.42	100	0.47	0.31	0.63	104	0.43	0.25	0.62
P. falciparum (csp)	10	0.55	0.42	0.67	100	0.40	0.25	0.57	104	0.52	0.33	0.70
P. falciparum (etramp5ag1)	10	0.31	0.21	0.44	100	0.27	0.15	0.44	104	0.42	0.24	0.61
P. falciparum (gexp18)	10	0.02	0.00	0.11	100	0.00	0.00	0.00	104	0.02	0.00	0.13
P. falciparum (glurpr2)	10	0.72	0.60	0.82	100	0.50	0.34	0.66	104	0.81	0.63	0.91
P. falciparum (pfama1)	10	0.97	0.87	0.99	100	0.75	0.59	0.87	104	0.96	0.85	0.99
P. falciparum (pfmsp119)	10	0.67	0.54	0.78	100	0.65	0.48	0.79	104	0.79	0.61	0.90
P. falciparum (rh42)	10	0.16	0.08	0.27	100	0.10	0.03	0.26	104	0.23	0.11	0.44
P. malariae (pmmsp119)	10	0.07	0.03	0.17	100	0.03	0.00	0.19	104	0.00	0.00	0.00
P. ovale (pomsp119)	10	0.03	0.01	0.13	100	0.02	0.00	0.15	104	0.04	0.01	0.24
P. vivax (pvdbprii)	10	0.00	0.00	0.00	100	0.00	0.00	0.00	104	0.00	0.00	0.00
P. vivax (pvmsp119)	10	0.00	0.00	0.00	100	0.00	0.00	0.00	104	0.00	0.00	0.00
P. vivax (pvrbp2b)	10	0.00	0.00	0.00	100	0.00	0.00	0.00	104	0.00	0.00	0.00
Brugia malayi (bm14)	10	0.09	0.04	0.19	100	0.00	0.00	0.00	104	0.12	0.04	0.32
Brugia malayi (bm33)	10	0.03	0.01	0.13	100	0.06	0.02	0.22	104	0.04	0.01	0.24
Chlamydia trachomatis (pgp3)	10	0.28	0.18	0.40	100	0.34	0.20	0.52	104	0.63	0.43	0.79
Chlamydia trachomatis (ct694)	10	0.12	0.06	0.23	100	0.42	0.26	0.59	104	0.58	0.38	0.75
Onchocerca volvulus (ov16)	10	0.00	0.00	0.00	100	0.00	0.00	0.00	104	0.00	0.00	0.00
Schistosoma mansoni (sea)	10	0.45	0.33	0.58	100	0.18	0.08	0.35	104	0.32	0.17	0.53
Schistosoma mansoni (sm25)	10	0.47	0.34	0.59	100	0.43	0.28	0.60	104	0.55	0.36	0.73
Strongyloides stercoralis (nie)	10	0.14	0.07	0.25	100	0.15	0.06	0.32	104	0.04	0.01	0.24
Taenia solium (es33)	10	0.05	0.02	0.15	100	0.10	0.03	0.26	104	0.00	0.00	0.00
Taenia solium (t24h)	10	0.19	0.11	0.31	100	0.10	0.03	0.26	104	0.10	0.03	0.29
Treponema palladium (rp17)	10	0.24	0.15	0.37	100	0.15	0.06	0.32	104	0.24	0.11	0.44
Treponema palladium (tmpa)	10	0.02	0.00	0.11	100	0.02	0.00	0.15	104	0.04	0.01	0.24
Wuchereria bancrofti (wb123)	10	0.24	0.15	0.37	100	0.10	0.03	0.26	104	0.17	0.07	0.38
Diphtheria (Dip tox)	10	0.84	0.73	0.92	100	0.90	0.76	0.96	104	0.87	0.69	0.95
Measles virus (wMev)	10	0.71	0.58	0.81	100	0.73	0.56	0.85	104	0.57	0.37	0.74
Rubella virus (wRuv)	10	0.76	0.63	0.85	100	0.97	0.81	1.00	104	0.90	0.77	0.96
Tetanus (Tet tox)	10	0.90	0.79	0.95	100	0.84	0.67	0.93	104	0.82	0.63	0.93

Antigen	Cluster Number	proportion	LCI	UCI	Cluster Number	proportion	LCI	UCI	Cluster Number	proportion	LCI	UCI
SARS-CoV-2 (sars2np)	116	0.06	0.02	0.15	24	0.18	0.06	0.43	25	0.00	0.00	0.00
SARS-CoV-2 (sars2rbd)	116	0.01	0.00	0.07	24	0.00	0.00	0.00	25	0.00	0.00	0.00
Cryptosporidium parvum (cp17)	116	0.86	0.74	0.93	24	0.85	0.60	0.95	25	0.93	0.66	0.99
Cryptosporidium parvum (cp23)	116	0.71	0.58	0.82	24	0.72	0.48	0.88	25	0.54	0.31	0.75
Giardia lamblia (vsp3)	116	0.38	0.26	0.52	24	0.38	0.19	0.62	25	0.60	0.37	0.80
Giardia lamblia (vsp5)	116	0.14	0.07	0.24	24	0.27	0.12	0.52	25	0.25	0.11	0.48
P. falciparum (csp)	116	0.56	0.42	0.68	24	0.52	0.29	0.73	25	0.41	0.21	0.65
P. falciparum (etramp5ag1)	116	0.33	0.22	0.47	24	0.30	0.13	0.55	25	0.15	0.05	0.40
P. falciparum (gexp18)	116	0.02	0.00	0.14	24	0.00	0.00	0.00	25	0.00	0.00	0.00
P. falciparum (glurpr2)	116	0.69	0.56	0.80	24	0.82	0.60	0.93	25	0.70	0.48	0.86
P. falciparum (pfama1)	116	0.91	0.81	0.96	24	0.84	0.65	0.94	25	0.80	0.56	0.92
P. falciparum (pfmsp119)	116	0.69	0.55	0.80	24	0.62	0.38	0.81	25	0.57	0.34	0.77
P. falciparum (rh42)	116	0.18	0.10	0.31	24	0.07	0.01	0.37	25	0.00	0.00	0.00
P. malariae (pmmsp119)	116	0.04	0.01	0.14	24	0.00	0.00	0.00	25	0.00	0.00	0.00
P. ovale (pomsp119)	116	0.01	0.00	0.07	24	0.08	0.02	0.29	25	0.00	0.00	0.00
P. vivax (pvdbprii)	116	0.00	0.00	0.00	24	0.00	0.00	0.00	25	0.00	0.00	0.00
P. vivax (pvmsp119)	116	0.00	0.00	0.00	24	0.00	0.00	0.00	25	0.00	0.00	0.00
P. vivax (pvrbp2b)	116	0.00	0.00	0.00	24	0.00	0.00	0.00	25	0.00	0.00	0.00
Brugia malayi (bm14)	116	0.12	0.06	0.24	24	0.07	0.01	0.37	25	0.02	0.00	0.16
Brugia malayi (bm33)	116	0.00	0.00	0.00	24	0.00	0.00	0.00	25	0.00	0.00	0.00
Chlamydia trachomatis (pgp3)	116	0.45	0.32	0.58	24	0.25	0.10	0.51	25	0.05	0.01	0.18
Chlamydia trachomatis (ct694)	116	0.45	0.32	0.58	24	0.32	0.14	0.57	25	0.18	0.06	0.42
Onchocerca volvulus (ov16)	116	0.02	0.00	0.13	24	0.00	0.00	0.00	25	0.07	0.01	0.34
Schistosoma mansoni (sea)	116	0.61	0.48	0.73	24	0.56	0.32	0.76	25	0.09	0.02	0.33
Schistosoma mansoni (sm25)	116	0.38	0.26	0.52	24	0.32	0.14	0.57	25	0.13	0.03	0.39
Strongyloides stercoralis (nie)	116	0.19	0.10	0.32	24	0.22	0.07	0.49	25	0.13	0.03	0.40
Taenia solium (es33)	116	0.07	0.03	0.19	24	0.00	0.00	0.00	25	0.00	0.00	0.00
Taenia solium (t24h)	116	0.05	0.02	0.16	24	0.14	0.04	0.42	25	0.02	0.00	0.16
Treponema palladium (rp17)	116	0.21	0.12	0.34	24	0.07	0.01	0.37	25	0.22	0.08	0.47
Treponema palladium (tmpa)	116	0.09	0.04	0.21	24	0.14	0.04	0.42	25	0.07	0.01	0.34
Wuchereria bancrofti (wb123)	116	0.20	0.12	0.33	24	0.07	0.01	0.37	25	0.02	0.00	0.16
Diphtheria (Dip tox)	116	0.93	0.82	0.97	24	0.80	0.55	0.93	25	0.61	0.37	0.81
Measles virus (wMev)	116	0.66	0.52	0.77	24	0.68	0.43	0.86	25	0.56	0.33	0.77
Rubella virus (wRuv)	116	0.70	0.56	0.81	24	0.95	0.71	0.99	25	0.87	0.61	0.97
Tetanus (Tet tox)	116	0.78	0.65	0.87	24	0.70	0.45	0.87	25	0.80	0.54	0.93

Antigen	Cluster Number	proportion	LCI	UCI	Cluster Number	proportion	LCI	UCI	Cluster Number	proportion	LCI	UCI
SARS-CoV-2 (sars2np)	27	0.02	0.00	0.14	31	0.04	0.01	0.13	33	0.08	0.03	0.18
SARS-CoV-2 (sars2rbd)	27	0.00	0.00	0.00	31	0.02	0.00	0.12	33	0.02	0.00	0.11
Cryptosporidium parvum (cp17)	27	0.96	0.84	0.99	31	0.71	0.58	0.81	33	0.93	0.84	0.98
Cryptosporidium parvum (cp23)	27	0.74	0.59	0.85	31	0.41	0.29	0.53	33	0.77	0.65	0.86
Giardia lamblia (vsp3)	27	0.74	0.59	0.85	31	0.33	0.22	0.45	33	0.49	0.37	0.62
Giardia lamblia (vsp5)	27	0.33	0.21	0.47	31	0.16	0.09	0.27	33	0.25	0.15	0.37
P. falciparum (csp)	27	0.85	0.71	0.93	31	0.54	0.42	0.66	33	0.62	0.50	0.74
P. falciparum (etramp5ag1)	27	0.54	0.40	0.68	31	0.25	0.16	0.37	33	0.31	0.21	0.44
P. falciparum (gexp18)	27	0.00	0.00	0.00	31	0.03	0.01	0.10	33	0.00	0.00	0.00
P. falciparum (glurpr2)	27	0.76	0.62	0.86	31	0.61	0.48	0.72	33	0.61	0.48	0.72
P. falciparum (pfama1)	27	1.00	0.00	0.00	31	0.82	0.70	0.90	33	0.87	0.76	0.93
P. falciparum (pfmsp119)	27	0.85	0.71	0.93	31	0.58	0.45	0.70	33	0.62	0.50	0.74
P. falciparum (rh42)	27	0.17	0.09	0.31	31	0.06	0.02	0.15	33	0.11	0.06	0.22
P. malariae (pmmsp119)	27	0.11	0.05	0.24	31	0.04	0.01	0.13	33	0.05	0.02	0.14
P. ovale (pomsp119)	27	0.04	0.01	0.16	31	0.02	0.00	0.11	33	0.03	0.01	0.12
P. vivax (pvdbprii)	27	0.00	0.00	0.00	31	0.00	0.00	0.00	33	0.00	0.00	0.00
P. vivax (pvmsp119)	27	0.00	0.00	0.00	31	0.00	0.00	0.00	33	0.02	0.00	0.11
P. vivax (pvrbp2b)	27	0.00	0.00	0.00	31	0.00	0.00	0.00	33	0.00	0.00	0.00
Brugia malayi (bm14)	27	0.04	0.01	0.16	31	0.05	0.02	0.15	33	0.00	0.00	0.00
Brugia malayi (bm33)	27	0.09	0.03	0.21	31	0.02	0.00	0.11	33	0.03	0.01	0.12
Chlamydia trachomatis (pgp3)	27	0.41	0.28	0.56	31	0.19	0.11	0.31	33	0.26	0.17	0.39
Chlamydia trachomatis (ct694)	27	0.20	0.10	0.34	31	0.15	0.08	0.26	33	0.13	0.07	0.24
Onchocerca volvulus (ov16)	27	0.07	0.02	0.18	31	0.00	0.00	0.00	33	0.00	0.00	0.00
Schistosoma mansoni (sea)	27	0.57	0.42	0.70	31	0.32	0.21	0.45	33	0.64	0.51	0.75
Schistosoma mansoni (sm25)	27	0.59	0.44	0.72	31	0.16	0.09	0.27	33	0.38	0.26	0.50
Strongyloides stercoralis (nie)	27	0.20	0.10	0.34	31	0.16	0.09	0.28	33	0.23	0.14	0.35
Taenia solium (es33)	27	0.11	0.05	0.24	31	0.02	0.00	0.11	33	0.08	0.03	0.18
Taenia solium (t24h)	27	0.13	0.06	0.26	31	0.05	0.02	0.13	33	0.08	0.03	0.18
Treponema palladium (rp17)	27	0.37	0.24	0.52	31	0.09	0.04	0.18	33	0.21	0.13	0.33
Treponema palladium (tmpa)	27	0.09	0.03	0.21	31	0.05	0.02	0.15	33	0.10	0.04	0.20
Wuchereria bancrofti (wb123)	27	0.26	0.15	0.41	31	0.17	0.10	0.29	33	0.18	0.10	0.30
Diphtheria (Dip tox)	27	0.91	0.79	0.97	31	0.68	0.56	0.79	33	0.89	0.78	0.94
Measles virus (wMev)	27	0.67	0.53	0.79	31	0.52	0.39	0.64	33	0.77	0.65	0.86
Rubella virus (wRuv)	27	0.85	0.71	0.93	31	0.65	0.52	0.76	33	0.80	0.68	0.88
Tetanus (Tet tox)	27	0.89	0.76	0.95	31	0.89	0.78	0.95	33	0.95	0.86	0.98

Antigen	Cluster Number	proportion	LCI	UCI	Cluster Number	proportion	LCI	UCI	Cluster Number	proportion	LCI	UCI
SARS-CoV-2 (sars2np)	39	0.05	0.02	0.16	4	0.08	0.02	0.27	40	0.35	0.25	0.47
SARS-CoV-2 (sars2rbd)	39	0.02	0.00	0.13	4	0.04	0.01	0.25	40	0.12	0.06	0.22
Cryptosporidium parvum (cp17)	39	0.95	0.84	0.98	4	0.60	0.41	0.77	40	0.95	0.86	0.98
Cryptosporidium parvum (cp23)	39	0.77	0.64	0.86	4	0.54	0.36	0.72	40	0.77	0.65	0.85
Giardia lamblia (vsp3)	39	0.54	0.41	0.67	4	0.10	0.03	0.27	40	0.40	0.29	0.52
Giardia lamblia (vsp5)	39	0.43	0.30	0.56	4	0.06	0.01	0.20	40	0.23	0.15	0.34
P. falciparum (csp)	39	0.51	0.38	0.64	4	0.20	0.09	0.41	40	0.37	0.26	0.49
P. falciparum (etramp5ag1)	39	0.45	0.32	0.58	4	0.15	0.06	0.35	40	0.30	0.20	0.42
P. falciparum (gexp18)	39	0.02	0.00	0.13	4	0.00	0.00	0.00	40	0.00	0.00	0.00
P. falciparum (glurpr2)	39	0.69	0.56	0.80	4	0.36	0.20	0.56	40	0.59	0.47	0.70
P. falciparum (pfama1)	39	0.81	0.69	0.89	4	0.60	0.41	0.76	40	0.64	0.52	0.75
P. falciparum (pfmsp119)	39	0.61	0.48	0.73	4	0.37	0.21	0.56	40	0.61	0.49	0.72
P. falciparum (rh42)	39	0.21	0.12	0.34	4	0.04	0.01	0.22	40	0.16	0.09	0.27
P. malariae (pmmsp119)	39	0.06	0.02	0.18	4	0.00	0.00	0.00	40	0.02	0.00	0.10
P. ovale (pomsp119)	39	0.03	0.01	0.10	4	0.00	0.00	0.00	40	0.03	0.01	0.12
P. vivax (pvdbprii)	39	0.00	0.00	0.00	4	0.00	0.00	0.00	40	0.00	0.00	0.00
P. vivax (pvmsp119)	39	0.02	0.00	0.13	4	0.00	0.00	0.00	40	0.05	0.02	0.14
P. vivax (pvrbp2b)	39	0.00	0.00	0.00	4	0.00	0.00	0.00	40	0.00	0.00	0.00
Brugia malayi (bm14)	39	0.06	0.02	0.18	4	0.00	0.00	0.00	40	0.08	0.03	0.18
Brugia malayi (bm33)	39	0.12	0.05	0.24	4	0.07	0.02	0.25	40	0.05	0.02	0.14
Chlamydia trachomatis (pgp3)	39	0.52	0.39	0.65	4	0.18	0.07	0.37	40	0.48	0.36	0.59
Chlamydia trachomatis (ct694)	39	0.52	0.39	0.65	4	0.34	0.19	0.54	40	0.44	0.33	0.56
Onchocerca volvulus (ov16)	39	0.00	0.00	0.00	4	0.00	0.00	0.00	40	0.00	0.00	0.00
Schistosoma mansoni (sea)	39	0.47	0.34	0.60	4	0.19	0.09	0.38	40	0.32	0.22	0.44
Schistosoma mansoni (sm25)	39	0.45	0.32	0.58	4	0.01	0.00	0.06	40	0.45	0.33	0.57
Strongyloides stercoralis (nie)	39	0.19	0.10	0.32	4	0.04	0.01	0.25	40	0.28	0.18	0.39
Taenia solium (es33)	39	0.05	0.02	0.16	4	0.05	0.01	0.20	40	0.05	0.02	0.14
Taenia solium (t24h)	39	0.21	0.12	0.34	4	0.04	0.01	0.25	40	0.13	0.07	0.23
Treponema palladium (rp17)	39	0.21	0.12	0.34	4	0.01	0.00	0.06	40	0.16	0.09	0.27
Treponema palladium (tmpa)	39	0.15	0.07	0.28	4	0.00	0.00	0.00	40	0.09	0.04	0.19
Wuchereria bancrofti (wb123)	39	0.26	0.16	0.40	4	0.08	0.02	0.27	40	0.19	0.11	0.30
Diphtheria (Dip tox)	39	0.95	0.84	0.98	4	0.72	0.52	0.85	40	0.85	0.74	0.92
Measles virus (wMev)	39	0.78	0.65	0.87	4	0.70	0.51	0.85	40	0.65	0.53	0.75
Rubella virus (wRuv)	39	0.86	0.75	0.93	4	0.88	0.72	0.96	40	0.90	0.80	0.95
Tetanus (Tet tox)	39	0.99	0.91	1.00	4	0.92	0.73	0.98	40	0.91	0.82	0.96

Antigen	Cluster Number	proportion	LCI	UCI	Cluster Number	proportion	LCI	UCI	Cluster Number	proportion	LCI	UCI
SARS-CoV-2 (sars2np)	41	0.11	0.05	0.23	42	0.00	0.00	0.00	48	0.04	0.01	0.21
SARS-CoV-2 (sars2rbd)	41	0.10	0.04	0.22	42	0.00	0.00	0.00	48	0.00	0.00	0.00
Cryptosporidium parvum (cp17)	41	0.88	0.76	0.94	42	1.00	0.00	0.00	48	0.95	0.81	0.99
Cryptosporidium parvum (cp23)	41	0.58	0.44	0.71	42	0.80	0.69	0.88	48	0.76	0.59	0.88
Giardia lamblia (vsp3)	41	0.46	0.32	0.60	42	0.84	0.73	0.91	48	0.72	0.54	0.85
Giardia lamblia (vsp5)	41	0.20	0.11	0.34	42	0.37	0.26	0.49	48	0.24	0.12	0.41
P. falciparum (csp)	41	0.79	0.66	0.88	42	0.83	0.72	0.90	48	0.57	0.39	0.73
P. falciparum (etramp5ag1)	41	0.33	0.22	0.48	42	0.52	0.40	0.64	48	0.41	0.25	0.59
P. falciparum (gexp18)	41	0.03	0.01	0.13	42	0.01	0.00	0.07	48	0.00	0.00	0.00
P. falciparum (glurpr2)	41	0.78	0.65	0.87	42	0.78	0.67	0.86	48	0.74	0.56	0.86
P. falciparum (pfama1)	41	0.92	0.80	0.97	42	0.96	0.87	0.99	48	0.94	0.78	0.98
P. falciparum (pfmsp119)	41	0.63	0.49	0.76	42	0.70	0.58	0.80	48	0.70	0.52	0.83
P. falciparum (rh42)	41	0.12	0.06	0.25	42	0.16	0.09	0.27	48	0.16	0.07	0.33
P. malariae (pmmsp119)	41	0.08	0.03	0.20	42	0.06	0.02	0.15	48	0.03	0.00	0.16
P. ovale (pomsp119)	41	0.03	0.01	0.09	42	0.09	0.04	0.18	48	0.00	0.00	0.00
P. vivax (pvdbprii)	41	0.00	0.00	0.00	42	0.00	0.00	0.00	48	0.00	0.00	0.00
P. vivax (pvmsp119)	41	0.00	0.00	0.00	42	0.00	0.00	0.00	48	0.00	0.00	0.00
P. vivax (pvrbp2b)	41	0.00	0.00	0.00	42	0.00	0.00	0.00	48	0.00	0.00	0.00
Brugia malayi (bm14)	41	0.08	0.03	0.20	42	0.31	0.21	0.43	48	0.13	0.05	0.31
Brugia malayi (bm33)	41	0.00	0.00	0.00	42	0.04	0.01	0.13	48	0.06	0.02	0.22
Chlamydia trachomatis (pgp3)	41	0.30	0.19	0.44	42	0.41	0.29	0.53	48	0.31	0.17	0.50
Chlamydia trachomatis (ct694)	41	0.28	0.17	0.42	42	0.23	0.14	0.35	48	0.28	0.15	0.46
Onchocerca volvulus (ov16)	41	0.02	0.00	0.14	42	0.03	0.01	0.10	48	0.00	0.00	0.00
Schistosoma mansoni (sea)	41	0.52	0.38	0.66	42	0.64	0.52	0.75	48	0.68	0.50	0.81
Schistosoma mansoni (sm25)	41	0.40	0.27	0.55	42	0.75	0.64	0.84	48	0.58	0.40	0.74
Strongyloides stercoralis (nie)	41	0.18	0.09	0.31	42	0.17	0.10	0.28	48	0.20	0.09	0.39
Taenia solium (es33)	41	0.08	0.03	0.20	42	0.14	0.07	0.24	48	0.00	0.00	0.00
Taenia solium (t24h)	41	0.05	0.01	0.16	42	0.25	0.16	0.38	48	0.10	0.03	0.26
Treponema palladium (rp17)	41	0.22	0.13	0.36	42	0.38	0.27	0.51	48	0.28	0.15	0.46
Treponema palladium (tmpa)	41	0.17	0.09	0.30	42	0.15	0.08	0.25	48	0.16	0.07	0.33
Wuchereria bancrofti (wb123)	41	0.24	0.14	0.38	42	0.47	0.35	0.59	48	0.45	0.28	0.62
Diphtheria (Dip tox)	41	0.83	0.70	0.91	42	0.83	0.73	0.91	48	0.96	0.79	0.99
Measles virus (wMev)	41	0.65	0.51	0.77	42	0.62	0.50	0.73	48	0.87	0.69	0.95
Rubella virus (wRuv)	41	0.83	0.70	0.91	42	0.65	0.53	0.75	48	0.88	0.71	0.95
Tetanus (Tet tox)	41	0.87	0.74	0.94	42	0.67	0.55	0.78	48	0.93	0.76	0.98

Antigen	Cluster Number	proportion	LCI	UCI	Cluster Number	proportion	LCI	UCI	Cluster Number	proportion	LCI	UCI
SARS-CoV-2 (sars2np)	52	0.03	0.00	0.20	54	0.00	0.00	0.00	56	0.00	0.00	0.00
SARS-CoV-2 (sars2rbd)	52	0.00	0.00	0.00	54	0.00	0.00	0.00	56	0.00	0.00	0.00
Cryptosporidium parvum (cp17)	52	0.92	0.74	0.98	54	0.95	0.71	0.99	56	0.84	0.66	0.93
Cryptosporidium parvum (cp23)	52	0.59	0.41	0.75	54	0.58	0.36	0.78	56	0.63	0.44	0.79
Giardia lamblia (vsp3)	52	0.45	0.28	0.63	54	0.55	0.33	0.75	56	0.20	0.09	0.39
Giardia lamblia (vsp5)	52	0.30	0.17	0.48	54	0.24	0.10	0.48	56	0.14	0.05	0.32
P. falciparum (csp)	52	0.81	0.62	0.91	54	0.76	0.53	0.89	56	0.51	0.33	0.69
P. falciparum (etramp5ag1)	52	0.37	0.22	0.56	54	0.39	0.20	0.63	56	0.41	0.24	0.60
P. falciparum (gexp18)	52	0.00	0.00	0.00	54	0.05	0.01	0.29	56	0.00	0.00	0.00
P. falciparum (glurpr2)	52	0.68	0.50	0.82	54	0.77	0.56	0.90	56	0.59	0.40	0.76
P. falciparum (pfama1)	52	1.00	0.00	0.00	54	0.96	0.78	1.00	56	0.89	0.71	0.96
P. falciparum (pfmsp119)	52	0.81	0.63	0.91	54	0.70	0.48	0.86	56	0.64	0.45	0.80
P. falciparum (rh42)	52	0.21	0.10	0.38	54	0.22	0.08	0.47	56	0.11	0.04	0.29
P. malariae (pmmsp119)	52	0.00	0.00	0.00	54	0.00	0.00	0.00	56	0.00	0.00	0.00
P. ovale (pomsp119)	52	0.00	0.00	0.00	54	0.00	0.00	0.00	56	0.04	0.01	0.24
P. vivax (pvdbprii)	52	0.00	0.00	0.00	54	0.00	0.00	0.00	56	0.00	0.00	0.00
P. vivax (pvmsp119)	52	0.00	0.00	0.00	54	0.00	0.00	0.00	56	0.00	0.00	0.00
P. vivax (pvrbp2b)	52	0.00	0.00	0.00	54	0.00	0.00	0.00	56	0.00	0.00	0.00
Brugia malayi (bm14)	52	0.03	0.00	0.20	54	0.00	0.00	0.00	56	0.08	0.02	0.28
Brugia malayi (bm33)	52	0.09	0.03	0.24	54	0.26	0.12	0.49	56	0.08	0.02	0.28
Chlamydia trachomatis (pgp3)	52	0.22	0.10	0.40	54	0.24	0.10	0.48	56	0.23	0.11	0.43
Chlamydia trachomatis (ct694)	52	0.17	0.07	0.35	54	0.17	0.06	0.42	56	0.08	0.02	0.28
Onchocerca volvulus (ov16)	52	0.04	0.01	0.23	54	0.00	0.00	0.00	56	0.00	0.00	0.00
Schistosoma mansoni (sea)	52	0.47	0.30	0.64	54	0.50	0.28	0.71	56	0.67	0.48	0.82
Schistosoma mansoni (sm25)	52	0.40	0.24	0.58	54	0.39	0.20	0.63	56	0.31	0.17	0.51
Strongyloides stercoralis (nie)	52	0.10	0.03	0.28	54	0.19	0.06	0.44	56	0.08	0.02	0.28
Taenia solium (es33)	52	0.00	0.00	0.00	54	0.00	0.00	0.00	56	0.00	0.00	0.00
Taenia solium (t24h)	52	0.10	0.03	0.28	54	0.05	0.01	0.29	56	0.00	0.00	0.00
Treponema palladium (rp17)	52	0.03	0.00	0.20	54	0.24	0.10	0.48	56	0.14	0.05	0.32
Treponema palladium (tmpa)	52	0.10	0.03	0.28	54	0.05	0.01	0.29	56	0.04	0.01	0.24
Wuchereria bancrofti (wb123)	52	0.04	0.01	0.23	54	0.41	0.22	0.64	56	0.08	0.02	0.28
Diphtheria (Dip tox)	52	0.66	0.48	0.81	54	0.76	0.53	0.89	56	0.72	0.52	0.86
Measles virus (wMev)	52	0.69	0.51	0.83	54	0.67	0.44	0.85	56	0.47	0.29	0.65
Rubella virus (wRuv)	52	0.72	0.54	0.85	54	0.72	0.49	0.88	56	0.74	0.54	0.87
Tetanus (Tet tox)	52	0.58	0.40	0.74	54	0.35	0.17	0.58	56	0.71	0.52	0.85

Antigen	Cluster Number	proportion	LCI	UCI	Cluster Number	proportion	LCI	UCI	Cluster Number	proportion	LCI	UCI
SARS-CoV-2 (sars2np)	6	0.12	0.04	0.31	64	0.15	0.09	0.26	65	0.06	0.02	0.17
SARS-CoV-2 (sars2rbd)	6	0.12	0.04	0.31	64	0.10	0.05	0.19	65	0.00	0.00	0.00
Cryptosporidium parvum (cp17)	6	0.94	0.78	0.99	64	0.81	0.70	0.88	65	0.98	0.93	1.00
Cryptosporidium parvum (cp23)	6	0.89	0.71	0.96	64	0.62	0.50	0.72	65	0.85	0.73	0.92
Giardia lamblia (vsp3)	6	0.26	0.13	0.46	64	0.35	0.25	0.46	65	0.69	0.55	0.79
Giardia lamblia (vsp5)	6	0.14	0.05	0.32	64	0.19	0.11	0.29	65	0.32	0.21	0.45
P. falciparum (csp)	6	0.28	0.14	0.48	64	0.36	0.26	0.47	65	0.92	0.82	0.96
P. falciparum (etramp5ag1)	6	0.18	0.07	0.37	64	0.28	0.19	0.39	65	0.30	0.19	0.43
P. falciparum (gexp18)	6	0.04	0.01	0.24	64	0.01	0.00	0.09	65	0.05	0.01	0.15
P. falciparum (glurpr2)	6	0.32	0.17	0.52	64	0.56	0.45	0.67	65	0.79	0.67	0.88
P. falciparum (pfama1)	6	0.58	0.39	0.75	64	0.83	0.73	0.90	65	0.96	0.88	0.99
P. falciparum (pfmsp119)	6	0.36	0.20	0.56	64	0.74	0.63	0.83	65	0.71	0.58	0.81
P. falciparum (rh42)	6	0.00	0.00	0.00	64	0.03	0.01	0.10	65	0.31	0.20	0.44
P. malariae (pmmsp119)	6	0.00	0.00	0.00	64	0.01	0.00	0.09	65	0.03	0.01	0.12
P. ovale (pomsp119)	6	0.04	0.01	0.24	64	0.01	0.00	0.09	65	0.08	0.03	0.19
P. vivax (pvdbprii)	6	0.00	0.00	0.00	64	0.00	0.00	0.00	65	0.00	0.00	0.00
P. vivax (pvmsp119)	6	0.04	0.01	0.24	64	0.00	0.00	0.00	65	0.00	0.00	0.00
P. vivax (pvrbp2b)	6	0.00	0.00	0.00	64	0.00	0.00	0.00	65	0.00	0.00	0.00
Brugia malayi (bm14)	6	0.08	0.02	0.27	64	0.04	0.01	0.12	65	0.19	0.10	0.31
Brugia malayi (bm33)	6	0.00	0.00	0.00	64	0.08	0.04	0.17	65	0.53	0.40	0.66
Chlamydia trachomatis (pgp3)	6	0.26	0.13	0.46	64	0.15	0.08	0.25	65	0.38	0.26	0.51
Chlamydia trachomatis (ct694)	6	0.24	0.11	0.44	64	0.17	0.10	0.27	65	0.28	0.17	0.42
Onchocerca volvulus (ov16)	6	0.00	0.00	0.00	64	0.00	0.00	0.00	65	0.02	0.00	0.13
Schistosoma mansoni (sea)	6	0.44	0.27	0.63	64	0.64	0.53	0.74	65	0.76	0.63	0.85
Schistosoma mansoni (sm25)	6	0.24	0.11	0.44	64	0.35	0.25	0.46	65	0.58	0.45	0.70
Strongyloides stercoralis (nie)	6	0.18	0.07	0.37	64	0.16	0.10	0.27	65	0.32	0.21	0.46
Taenia solium (es33)	6	0.10	0.03	0.28	64	0.01	0.00	0.09	65	0.14	0.07	0.26
Taenia solium (t24h)	6	0.08	0.02	0.27	64	0.08	0.04	0.17	65	0.28	0.17	0.41
Treponema palladium (rp17)	6	0.10	0.03	0.28	64	0.13	0.07	0.22	65	0.30	0.19	0.43
Treponema palladium (tmpa)	6	0.04	0.01	0.24	64	0.05	0.02	0.12	65	0.14	0.07	0.26
Wuchereria bancrofti (wb123)	6	0.02	0.00	0.11	64	0.10	0.05	0.20	65	0.68	0.55	0.79
Diphtheria (Dip tox)	6	0.86	0.68	0.95	64	0.89	0.80	0.95	65	0.90	0.80	0.95
Measles virus (wMev)	6	0.78	0.59	0.90	64	0.72	0.61	0.81	65	0.76	0.63	0.85
Rubella virus (wRuv)	6	0.93	0.77	0.98	64	0.83	0.73	0.90	65	0.86	0.76	0.92
Tetanus (Tet tox)	6	0.98	0.89	1.00	64	0.91	0.82	0.96	65	0.69	0.56	0.80

Antigen	Cluster Number	proportion	LCI	UCI	Cluster Number	proportion	LCI	UCI	Cluster Number	proportion	LCI	UCI
SARS-CoV-2 (sars2np)	72	0.05	0.01	0.16	79	0.00	0.00	0.00	81	0.02	0.00	0.12
SARS-CoV-2 (sars2rbd)	72	0.00	0.00	0.00	79	0.00	0.00	0.00	81	0.02	0.00	0.12
Cryptosporidium parvum (cp17)	72	0.88	0.75	0.94	79	0.82	0.61	0.93	81	0.96	0.86	0.99
Cryptosporidium parvum (cp23)	72	0.75	0.61	0.85	79	0.56	0.36	0.75	81	0.46	0.33	0.60
Giardia lamblia (vsp3)	72	0.42	0.30	0.56	79	0.60	0.39	0.78	81	0.60	0.46	0.72
Giardia lamblia (vsp5)	72	0.20	0.11	0.33	79	0.20	0.08	0.40	81	0.31	0.20	0.44
P. falciparum (csp)	72	0.64	0.50	0.76	79	0.79	0.58	0.91	81	0.71	0.57	0.82
P. falciparum (etramp5ag1)	72	0.31	0.20	0.45	79	0.34	0.18	0.55	81	0.50	0.37	0.63
P. falciparum (gexp18)	72	0.00	0.00	0.00	79	0.00	0.00	0.00	81	0.04	0.01	0.14
P. falciparum (glurpr2)	72	0.65	0.51	0.77	79	0.80	0.60	0.92	81	0.69	0.56	0.80
P. falciparum (pfama1)	72	0.91	0.79	0.96	79	0.95	0.73	0.99	81	0.94	0.84	0.98
P. falciparum (pfmsp119)	72	0.54	0.40	0.67	79	0.65	0.43	0.81	81	0.73	0.60	0.83
P. falciparum (rh42)	72	0.04	0.01	0.11	79	0.10	0.02	0.31	81	0.21	0.12	0.34
P. malariae (pmmsp119)	72	0.01	0.00	0.08	79	0.10	0.02	0.31	81	0.04	0.01	0.14
P. ovale (pomsp119)	72	0.00	0.00	0.00	79	0.00	0.00	0.00	81	0.02	0.00	0.12
P. vivax (pvdbprii)	72	0.00	0.00	0.00	79	0.00	0.00	0.00	81	0.00	0.00	0.00
P. vivax (pvmsp119)	72	0.00	0.00	0.00	79	0.00	0.00	0.00	81	0.02	0.00	0.12
P. vivax (pvrbp2b)	72	0.00	0.00	0.00	79	0.00	0.00	0.00	81	0.00	0.00	0.00
Brugia malayi (bm14)	72	0.11	0.05	0.24	79	0.18	0.07	0.39	81	0.10	0.04	0.21
Brugia malayi (bm33)	72	0.12	0.05	0.23	79	0.03	0.00	0.20	81	0.31	0.20	0.44
Chlamydia trachomatis (pgp3)	72	0.41	0.28	0.55	79	0.50	0.30	0.69	81	0.48	0.35	0.61
Chlamydia trachomatis (ct694)	72	0.32	0.21	0.47	79	0.46	0.27	0.67	81	0.29	0.18	0.43
Onchocerca volvulus (ov16)	72	0.03	0.01	0.13	79	0.00	0.00	0.00	81	0.02	0.00	0.12
Schistosoma mansoni (sea)	72	0.54	0.40	0.67	79	0.63	0.42	0.80	81	0.60	0.46	0.72
Schistosoma mansoni (sm25)	72	0.57	0.43	0.70	79	0.45	0.26	0.65	81	0.77	0.64	0.86
Strongyloides stercoralis (nie)	72	0.14	0.07	0.28	79	0.21	0.09	0.42	81	0.25	0.15	0.38
Taenia solium (es33)	72	0.04	0.01	0.14	79	0.08	0.02	0.28	81	0.02	0.00	0.12
Taenia solium (t24h)	72	0.17	0.09	0.30	79	0.22	0.10	0.44	81	0.38	0.26	0.52
Treponema palladium (rp17)	72	0.16	0.08	0.29	79	0.19	0.07	0.41	81	0.48	0.35	0.61
Treponema palladium (tmpa)	72	0.07	0.03	0.18	79	0.10	0.02	0.31	81	0.13	0.07	0.26
Wuchereria bancrofti (wb123)	72	0.22	0.13	0.36	79	0.40	0.22	0.61	81	0.60	0.46	0.72
Diphtheria (Dip tox)	72	0.79	0.66	0.88	79	0.80	0.60	0.92	81	0.90	0.79	0.96
Measles virus (wMev)	72	0.42	0.29	0.56	79	0.53	0.33	0.72	81	0.58	0.44	0.70
Rubella virus (wRuv)	72	0.78	0.65	0.87	79	0.67	0.46	0.83	81	0.81	0.68	0.89
Tetanus (Tet tox)	72	0.75	0.61	0.85	79	0.79	0.58	0.91	81	0.83	0.70	0.91

Antigen	Cluster Number	proportion	LCI	UCI	Cluster Number	proportion	LCI	UCI	Cluster Number	proportion	LCI	UCI
SARS-CoV-2 (sars2np)	82	0.01	0.00	0.09	83	0.00	0.00	0.00	84	0.00	0.00	0.00
SARS-CoV-2 (sars2rbd)	82	0.03	0.00	0.18	83	0.00	0.00	0.00	84	0.00	0.00	0.00
Cryptosporidium parvum (cp17)	82	0.94	0.79	0.99	83	0.97	0.89	0.99	84	0.87	0.70	0.95
Cryptosporidium parvum (cp23)	82	0.76	0.59	0.88	83	0.75	0.57	0.87	84	0.69	0.51	0.83
Giardia lamblia (vsp3)	82	0.52	0.35	0.68	83	0.55	0.38	0.71	84	0.40	0.25	0.58
Giardia lamblia (vsp5)	82	0.20	0.10	0.37	83	0.23	0.11	0.40	84	0.29	0.16	0.46
P. falciparum (csp)	82	0.89	0.74	0.96	83	0.81	0.65	0.91	84	0.26	0.14	0.45
P. falciparum (etramp5ag1)	82	0.48	0.32	0.64	83	0.38	0.23	0.55	84	0.10	0.03	0.28
P. falciparum (gexp18)	82	0.03	0.00	0.21	83	0.00	0.00	0.00	84	0.00	0.00	0.00
P. falciparum (glurpr2)	82	0.90	0.75	0.97	83	0.88	0.73	0.95	84	0.44	0.28	0.62
P. falciparum (pfama1)	82	1.00	0.00	0.00	83	0.94	0.86	0.98	84	0.64	0.47	0.79
P. falciparum (pfmsp119)	82	0.79	0.63	0.89	83	0.83	0.67	0.92	84	0.67	0.50	0.81
P. falciparum (rh42)	82	0.17	0.08	0.34	83	0.19	0.09	0.36	84	0.06	0.02	0.21
P. malariae (pmmsp119)	82	0.13	0.05	0.29	83	0.00	0.00	0.00	84	0.00	0.00	0.00
P. ovale (pomsp119)	82	0.01	0.00	0.09	83	0.04	0.01	0.23	84	0.00	0.00	0.00
P. vivax (pvdbprii)	82	0.00	0.00	0.00	83	0.00	0.00	0.00	84	0.00	0.00	0.00
P. vivax (pvmsp119)	82	0.00	0.00	0.00	83	0.00	0.00	0.00	84	0.03	0.00	0.19
P. vivax (pvrbp2b)	82	0.00	0.00	0.00	83	0.00	0.00	0.00	84	0.00	0.00	0.00
Brugia malayi (bm14)	82	0.18	0.08	0.35	83	0.12	0.05	0.28	84	0.00	0.00	0.00
Brugia malayi (bm33)	82	0.11	0.04	0.26	83	0.77	0.61	0.88	84	0.00	0.00	0.00
Chlamydia trachomatis (pgp3)	82	0.36	0.22	0.54	83	0.66	0.49	0.79	84	0.20	0.09	0.39
Chlamydia trachomatis (ct694)	82	0.26	0.14	0.43	83	0.61	0.44	0.76	84	0.14	0.05	0.33
Onchocerca volvulus (ov16)	82	0.00	0.00	0.00	83	0.21	0.10	0.39	84	0.00	0.00	0.00
Schistosoma mansoni (sea)	82	0.79	0.63	0.89	83	0.47	0.31	0.64	84	0.45	0.28	0.62
Schistosoma mansoni (sm25)	82	0.58	0.41	0.73	83	0.55	0.38	0.71	84	0.30	0.17	0.49
Strongyloides stercoralis (nie)	82	0.15	0.06	0.31	83	0.14	0.06	0.29	84	0.03	0.00	0.19
Taenia solium (es33)	82	0.12	0.04	0.28	83	0.15	0.06	0.32	84	0.03	0.00	0.19
Taenia solium (t24h)	82	0.16	0.07	0.32	83	0.13	0.05	0.31	84	0.10	0.03	0.28
Treponema palladium (rp17)	82	0.20	0.10	0.37	83	0.34	0.20	0.51	84	0.03	0.00	0.19
Treponema palladium (tmpa)	82	0.17	0.08	0.34	83	0.24	0.12	0.41	84	0.00	0.00	0.00
Wuchereria bancrofti (wb123)	82	0.25	0.13	0.41	83	0.43	0.27	0.60	84	0.09	0.03	0.23
Diphtheria (Dip tox)	82	0.97	0.79	1.00	83	0.88	0.72	0.95	84	0.84	0.67	0.93
Measles virus (wMev)	82	0.66	0.48	0.80	83	0.66	0.48	0.80	84	0.70	0.53	0.83
Rubella virus (wRuv)	82	0.81	0.65	0.91	83	0.83	0.67	0.92	84	0.86	0.73	0.93
Tetanus (Tet tox)	82	0.91	0.77	0.97	83	0.46	0.30	0.63	84	0.83	0.64	0.93

Antigen	Cluster Number	proportion	LCI	UCI	Cluster Number	proportion	LCI	UCI	Cluster Number	proportion	LCI	UCI
SARS-CoV-2 (sars2np)	85	0.00	0.00	0.00	87	0.00	0.00	0.00	94	0.02	0.00	0.14
SARS-CoV-2 (sars2rbd)	85	0.00	0.00	0.00	87	0.00	0.00	0.00	94	0.00	0.00	0.00
Cryptosporidium parvum (cp17)	85	0.61	0.42	0.77	87	0.90	0.74	0.97	94	0.95	0.84	0.99
Cryptosporidium parvum (cp23)	85	0.31	0.16	0.50	87	0.61	0.43	0.76	94	0.75	0.60	0.86
Giardia lamblia (vsp3)	85	0.17	0.07	0.36	87	0.42	0.26	0.60	94	0.61	0.46	0.74
Giardia lamblia (vsp5)	85	0.07	0.02	0.22	87	0.35	0.20	0.53	94	0.39	0.26	0.54
P. falciparum (csp)	85	0.28	0.14	0.48	87	0.21	0.10	0.39	94	0.75	0.60	0.86
P. falciparum (etramp5ag1)	85	0.14	0.05	0.32	87	0.10	0.03	0.27	94	0.41	0.27	0.56
P. falciparum (gexp18)	85	0.00	0.00	0.00	87	0.00	0.00	0.00	94	0.00	0.00	0.00
P. falciparum (glurpr2)	85	0.63	0.44	0.78	87	0.42	0.26	0.60	94	0.73	0.58	0.84
P. falciparum (pfama1)	85	0.83	0.65	0.93	87	0.66	0.48	0.80	94	1.00	0.00	0.00
P. falciparum (pfmsp119)	85	0.69	0.50	0.84	87	0.48	0.31	0.65	94	0.73	0.58	0.84
P. falciparum (rh42)	85	0.06	0.01	0.24	87	0.03	0.00	0.21	94	0.20	0.11	0.35
P. malariae (pmmsp119)	85	0.02	0.00	0.10	87	0.00	0.00	0.00	94	0.07	0.02	0.19
P. ovale (pomsp119)	85	0.00	0.00	0.00	87	0.03	0.00	0.21	94	0.00	0.00	0.00
P. vivax (pvdbprii)	85	0.00	0.00	0.00	87	0.00	0.00	0.00	94	0.00	0.00	0.00
P. vivax (pvmsp119)	85	0.00	0.00	0.00	87	0.00	0.00	0.00	94	0.00	0.00	0.00
P. vivax (pvrbp2b)	85	0.00	0.00	0.00	87	0.00	0.00	0.00	94	0.02	0.00	0.14
Brugia malayi (bm14)	85	0.00	0.00	0.00	87	0.00	0.00	0.00	94	0.05	0.01	0.16
Brugia malayi (bm33)	85	0.00	0.00	0.00	87	0.07	0.02	0.23	94	0.05	0.01	0.16
Chlamydia trachomatis (pgp3)	85	0.04	0.01	0.23	87	0.38	0.22	0.56	94	0.25	0.14	0.40
Chlamydia trachomatis (ct694)	85	0.00	0.00	0.00	87	0.41	0.25	0.59	94	0.23	0.13	0.37
Onchocerca volvulus (ov16)	85	0.00	0.00	0.00	87	0.00	0.00	0.00	94	0.00	0.00	0.00
Schistosoma mansoni (sea)	85	0.36	0.20	0.56	87	0.34	0.19	0.52	94	0.80	0.65	0.89
Schistosoma mansoni (sm25)	85	0.18	0.07	0.37	87	0.41	0.25	0.59	94	0.75	0.60	0.86
Strongyloides stercoralis (nie)	85	0.10	0.03	0.28	87	0.03	0.00	0.21	94	0.16	0.08	0.30
Taenia solium (es33)	85	0.00	0.00	0.00	87	0.03	0.00	0.21	94	0.11	0.05	0.25
Taenia solium (t24h)	85	0.00	0.00	0.00	87	0.10	0.03	0.28	94	0.18	0.09	0.32
Treponema palladium (rp17)	85	0.12	0.04	0.32	87	0.03	0.00	0.21	94	0.30	0.18	0.45
Treponema palladium (tmpa)	85	0.05	0.01	0.21	87	0.00	0.00	0.00	94	0.05	0.01	0.16
Wuchereria bancrofti (wb123)	85	0.04	0.01	0.23	87	0.13	0.05	0.30	94	0.18	0.09	0.32
Diphtheria (Dip tox)	85	0.57	0.38	0.74	87	0.86	0.68	0.95	94	0.93	0.81	0.98
Measles virus (wMev)	85	0.46	0.29	0.65	87	0.76	0.58	0.88	94	0.77	0.63	0.87
Rubella virus (wRuv)	85	0.72	0.52	0.86	87	0.91	0.75	0.97	94	0.91	0.78	0.97
Tetanus (Tet tox)	85	0.68	0.49	0.83	87	0.93	0.76	0.98	94	0.86	0.73	0.94

Supplementary Table 5. Adjust odds ratios from multilevel model for seropositivity in urban vs rural clusters

Antigen	OR Urban vs. Rural	Lower 95% CI	Upper 95% CI
SARS-CoV-2 (sars2np)	3.27	0.74	14.18
SARS-CoV-2 (sars2rbd)	5.73	0.82	42.71
Cryptosporidium parvum (cp17)	0.68	0.44	1.09
Cryptosporidium parvum (cp23)	1.09	0.56	2.29
Giardia lamblia (vsp3)	0.55	0.31	0.99
Giardia lamblia (vsp5)	0.69	0.41	1.10
P. falciparum (csp)	0.34	0.14	0.80
P. falciparum (etramp5ag1)	0.86	0.54	1.35
P. falciparum (gexp18)	0.64	0.25	1.46
P. falciparum (glurpr2)	0.61	0.35	1.07
P. falciparum (pfama1)	0.38	0.15	0.93
P. falciparum (pfmsp119)	0.74	0.38	1.43
P. falciparum (rh42)	0.19	0.07	0.46
P. malariae (pmmsp119)	0.47	0.17	1.22
P. ovale (pomsp119)	1.24	0.54	2.79
P. vivax (pvdbprii)	0.66	0.17	2.15
P. vivax (pvmsp119)	0.24	0.03	1.18
P. vivax (pvrbp2b)	0.51	0.16	1.57
Brugia malayi (bm14)	0.62	0.18	2.02
Brugia malayi (bm33)	0.46	0.19	1.08
Chlamydia trachomatis (pgp3)	0.91	0.41	2.06
Chlamydia trachomatis (ct694)	0.53	0.27	1.05
Onchocerca volvulus (ov16)	0.32	0.01	5.52
Schistosoma mansoni (sea)	0.79	0.38	1.66
Schistosoma mansoni (sm25)	0.57	0.34	0.92
Strongyloides stercoralis (nie)	1.23	0.65	2.31
Taenia solium (es33)	0.75	0.37	1.51
Taenia solium (t24h)	0.77	0.34	1.78
Treponema palladium (rp17)	0.58	0.27	1.12
Treponema palladium (tmpa)	0.26	0.04	1.20
Wuchereria bancrofti (wb123)	0.28	0.10	0.78
Diphtheria (Dip tox)	0.93	0.53	1.68
Measles virus (wMev)	1.62	0.96	2.66
Rubella virus (wRuv)	2.19	1.16	4.35
Tetanus (Tet tox)	2.31	1.13	4.70

Supplementary Table 6. Table showing results of all individual level associations (odds ratios) between antigen specific seroprevalence

Outcome Antigen	Regressor Antigen	Adjusted OR	Lower 95% CI	Lower 95% CI	P Val	Adj p val
"SARS-CoV-2(sars2np)"	"SARS-CoV-2(sars2rbd)"	718.77	180.81	3814.7	0	0
"SARS-CoV-2(sars2np)"	"Cryptosporidium parvum(cp17)"	1.82	0.61	6.75	0.32	0.66
"SARS-CoV-2(sars2np)"	"Cryptosporidium parvum(cp23)"	1.77	0.79	4.27	0.18	0.51
"SARS-CoV-2(sars2np)"	"Giardia lamblia(vsp3)"	0.41	0.16	0.97	0.05	0.27
"SARS-CoV-2(sars2np)"	"Giardia lamblia(vsp5)"	0.45	0.15	1.26	0.15	0.46
"SARS-CoV-2(sars2np)"	"P. falciparum(csp)"	1.07	0.46	2.53	0.87	0.96
"SARS-CoV-2(sars2np)"	"P. falciparum(etramp5ag1)"	1.04	0.43	2.38	0.94	0.99
"SARS-CoV-2(sars2np)"	"P. falciparum(gexp18)"	0.76	0.17	2.76	0.7	0.91
"SARS-CoV-2(sars2np)"	"P. falciparum(glurpr2)"	0.82	0.35	1.92	0.65	0.89
"SARS-CoV-2(sars2np)"	"P. falciparum(pfama1)"	0.28	0.12	0.62	0	0.04
"SARS-CoV-2(sars2np)"	"P. falciparum(pfmsp119)"	0.83	0.39	1.74	0.62	0.87
"SARS-CoV-2(sars2np)"	"P. falciparum(rh42)"	5.2	2.13	12.54	0	0.01
"SARS-CoV-2(sars2np)"	"P. malariae(pmmsp119)"	1.6	0.6	4.05	0.33	0.67
"SARS-CoV-2(sars2np)"	"P. ovale(pomsp119)"	2.17	0.72	5.98	0.15	0.45
"SARS-CoV-2(sars2np)"	"P. vivax(pvdbprii)"	0.44	0.03	5.76	0.54	0.83
"SARS-CoV-2(sars2np)"	"P. vivax(pvmsp119)"	0.14	0.01	1.27	0.12	0.42
"SARS-CoV-2(sars2np)"	"P. vivax(pvrbp2b)"	0.53	0.03	5.32	0.62	0.87
"SARS-CoV-2(sars2np)"	"Brugia malayi (bm14)"	1.15	0.34	3.29	0.81	0.96
"SARS-CoV-2(sars2np)"	"Brugia malayi (bm33)"	0.48	0.19	1.12	0.1	0.36
"SARS-CoV-2(sars2np)"	"Chlamydia trachomatis (ct694)"	0.29	0.11	0.73	0.01	0.12
"SARS-CoV-2(sars2np)"	"Chlamydia trachomatis (pgp3)"	1.44	0.6	3.37	0.41	0.74
"SARS-CoV-2(sars2np)"	"Onchocerca volvulus (ov16)"	0.49	0.01	10.8	0.74	0.93
"SARS-CoV-2(sars2np)"	"Schistosoma mansoni (sea)"	0.76	0.35	1.67	0.49	0.79
"SARS-CoV-2(sars2np)"	"Schistosoma mansoni (sm25)"	2.25	0.97	5.37	0.06	0.29
"SARS-CoV-2(sars2np)"	"Strongyloides stercoralis (nie)"	1.4	0.6	3.13	0.43	0.76
"SARS-CoV-2(sars2np)"	"Taenia solium(es33)"	1.31	0.45	3.4	0.6	0.86
"SARS-CoV-2(sars2np)"	"Taenia solium(t24h)"	1.43	0.56	3.49	0.44	0.76
"SARS-CoV-2(sars2np)"	"Treponema palladium (rp17)"	1.17	0.41	3.06	0.76	0.94
"SARS-CoV-2(sars2np)"	"Treponema palladium (tmpa)"	0.43	0.02	2.81	0.46	0.78
"SARS-CoV-2(sars2np)"	"Wuchereria bancrofti (wb123)"	1.19	0.45	3.06	0.72	0.92
"SARS-CoV-2(sars2np)"	"Diptheria (Dip tox)"	0.44	0.22	0.88	0.02	0.16
"SARS-CoV-2(sars2np)"	"Measles (wMev)"	2.06	0.91	5.17	0.1	0.36
"SARS-CoV-2(sars2np)"	"Rubella (wRuv)"	4.14	1.13	23.88	0.06	0.29
"SARS-CoV-2(sars2np)"	"Tetanus (Tet tox)"	2.1	1.07	4.31	0.03	0.23
"SARS-CoV-2(sars2rbd)"	"SARS-CoV-2(sars2np)"	2016.78	280.85	30707.02	0	0
"SARS-CoV-2(sars2rbd)"	"Cryptosporidium parvum(cp17)"	1.53	0.2	16.15	0.7	0.91
"SARS-CoV-2(sars2rbd)"	"Cryptosporidium parvum(cp23)"	0.23	0.04	1.29	0.1	0.36
"SARS-CoV-2(sars2rbd)"	"Giardia lamblia(vsp3)"	0.91	0.12	6.64	0.93	0.98
"SARS-CoV-2(sars2rbd)"	"Giardia lamblia(vsp5)"	14.26	1.76	159.49	0.02	0.16
"SARS-CoV-2(sars2rbd)"	"P. falciparum(csp)"	0.45	0.07	2.48	0.38	0.72
"SARS-CoV-2(sars2rbd)"	"P. falciparum(etramp5ag1)"	0.44	0.04	3.61	0.47	0.78
"SARS-CoV-2(sars2rbd)"	"P. falciparum(gexp18)"	24.63	1.99	362.26	0.01	0.13
"SARS-CoV-2(sars2rbd)"	"P. falciparum(glurpr2)"	1.45	0.25	8.98	0.68	0.9

"SARS-CoV-2(sars2rbd)"	"P. falciparum(pfama1)"	2.78	0.44	22.18	0.3	0.65
"SARS-CoV-2(sars2rbd)"	"P. falciparum(pfmsp119)"	1.6	0.31	7.92	0.56	0.85
"SARS-CoV-2(sars2rbd)"	"P. falciparum(rh42)"	0.01	0	0.18	0.01	0.08
"SARS-CoV-2(sars2rbd)"	"P. malariae(pmmsp119)"	2.58	0.19	33.21	0.46	0.78
"SARS-CoV-2(sars2rbd)"	"P. ovale(pomsp119)"	3.27	0.3	30	0.3	0.65
"SARS-CoV-2(sars2rbd)"	"P. vivax(pvdbprii)"	5.11	0.04	392.42	0.5	0.79
"SARS-CoV-2(sars2rbd)"	"P. vivax(pvmsp119)"	2.54	0.06	110.19	0.63	0.88
"SARS-CoV-2(sars2rbd)"	"P. vivax(pvrbp2b)"	1.67	0.02	202	0.83	0.96
"SARS-CoV-2(sars2rbd)"	"Brugia malayi (bm14)"	0.96	0.01	29.66	0.98	1
"SARS-CoV-2(sars2rbd)"	"Brugia malayi (bm33)"	0.69	0.07	5.18	0.72	0.92
"SARS-CoV-2(sars2rbd)"	"Chlamydia trachomatis (ct694)"	1.88	0.2	19.22	0.58	0.86
"SARS-CoV-2(sars2rbd)"	"Chlamydia trachomatis (pgp3)"	1.98	0.29	13.07	0.48	0.79
"SARS-CoV-2(sars2rbd)"	"Onchocerca volvulus (ov16)"	13.25	0.03	796.93	0.29	0.64
"SARS-CoV-2(sars2rbd)"	"Schistosoma mansoni (sea)"	1.66	0.32	9.83	0.55	0.84
"SARS-CoV-2(sars2rbd)"	"Schistosoma mansoni (sm25)"	1.7	0.36	8.91	0.51	0.8
"SARS-CoV-2(sars2rbd)"	"Strongyloides stercoralis (nie)"	0.55	0.05	4.34	0.59	0.86
"SARS-CoV-2(sars2rbd)"	"Taenia solium(es33)"	1.72	0.16	15.52	0.64	0.88
"SARS-CoV-2(sars2rbd)"	"Taenia solium(t24h)"	0.03	0	0.43	0.03	0.2
"SARS-CoV-2(sars2rbd)"	"Treponema palladium (rp17)"	0.21	0.01	2.67	0.3	0.65
"SARS-CoV-2(sars2rbd)"	"Treponema palladium (tmpa)"	0	NA	1.56816554391941e+54	0.99	1
"SARS-CoV-2(sars2rbd)"	"Wuchereria bancrofti (wb123)"	0.2	0.01	2.52	0.24	0.59
"SARS-CoV-2(sars2rbd)"	"Diptheria (Dip tox)"	0.97	0.2	4.83	0.97	1
"SARS-CoV-2(sars2rbd)"	"Measles (wMev)"	0.33	0.06	1.61	0.18	0.5
"SARS-CoV-2(sars2rbd)"	"Rubella (wRuv)"	0.36	0.04	3.5	0.37	0.72
"SARS-CoV-2(sars2rbd)"	"Tetanus (Tet tox)"	0.62	0.13	3.04	0.55	0.84
"Cryptosporidium parvum(cp17)"	"SARS-CoV-2(sars2np)"	1.62	0.53	5.88	0.43	0.76
"Cryptosporidium parvum(cp17)"	"SARS-CoV-2(sars2rbd)"	1.3	0.26	8.15	0.76	0.94
"Cryptosporidium parvum(cp17)"	"Cryptosporidium parvum(cp23)"	6.64	4.46	10.04	0	0
"Cryptosporidium parvum(cp17)"	"Giardia lamblia(vsp3)"	0.97	0.55	1.73	0.91	0.98
"Cryptosporidium parvum(cp17)"	"Giardia lamblia(vsp5)"	1.25	0.65	2.42	0.5	0.8
"Cryptosporidium parvum(cp17)"	"P. falciparum(csp)"	1.33	0.78	2.3	0.29	0.64
"Cryptosporidium parvum(cp17)"	"P. falciparum(etramp5ag1)"	1.42	0.75	2.81	0.3	0.65
"Cryptosporidium parvum(cp17)"	"P. falciparum(gexp18)"	0.28	0.1	0.96	0.03	0.2
"Cryptosporidium parvum(cp17)"	"P. falciparum(glurpr2)"	1.51	0.94	2.43	0.09	0.35
"Cryptosporidium parvum(cp17)"	"P. falciparum(pfama1)"	1.06	0.67	1.67	0.82	0.96
"Cryptosporidium parvum(cp17)"	"P. falciparum(pfmsp119)"	0.52	0.34	0.8	0	0.04
"Cryptosporidium parvum(cp17)"	"P. falciparum(rh42)"	4.26	1.42	18.57	0.02	0.18
"Cryptosporidium parvum(cp17)"	"P. malariae(pmmsp119)"	1.76	0.75	4.7	0.22	0.57
"Cryptosporidium parvum(cp17)"	"P. ovale(pomsp119)"	1.04	0.41	3	0.94	0.99
"Cryptosporidium parvum(cp17)"	"P. vivax(pvdbprii)"	0.37	0.07	2.38	0.27	0.62
"Cryptosporidium parvum(cp17)"	"P. vivax(pvmsp119)"	1721570.83	1.46156338056539e+76	4.81682100359159e+122	0.98	1
"Cryptosporidium parvum(cp17)"	"P. vivax(pvrbp2b)"	1.09	0.19	5.93	0.92	0.98
"Cryptosporidium parvum(cp17)"	"Brugia malayi (bm14)"	0.29	0.13	0.72	0.01	0.07
"Cryptosporidium parvum(cp17)"	"Brugia malayi (bm33)"	1.42	0.86	2.39	0.18	0.5
"Cryptosporidium parvum(cp17)"	"Chlamydia trachomatis (ct694)"	1.2	0.62	2.37	0.6	0.86
"Cryptosporidium parvum(cp17)"	"Chlamydia trachomatis (pgp3)"	0.81	0.42	1.59	0.54	0.83

"Cryptosporidium parvum(cp17)"	"Onchocerca volvulus (ov16)"	0.79	0.12	7.69	0.82	0.96
"Cryptosporidium parvum(cp17)"	"Schistosoma mansoni (sea)"	1.44	0.96	2.15	0.08	0.33
"Cryptosporidium parvum(cp17)"	"Schistosoma mansoni (sm25)"	1.16	0.71	1.88	0.55	0.84
"Cryptosporidium parvum(cp17)"	"Strongyloides stercoralis (nie)"	2.23	1.03	5.34	0.05	0.27
"Cryptosporidium parvum(cp17)"	"Taenia solium(es33)"	0.85	0.37	2.18	0.72	0.92
"Cryptosporidium parvum(cp17)"	"Taenia solium(t24h)"	5.98	1.53	41.34	0.03	0.2
"Cryptosporidium parvum(cp17)"	"Treponema palladium (rp17)"	1.08	0.53	2.37	0.83	0.96
"Cryptosporidium parvum(cp17)"	"Treponema palladium (tmpa)"	0.43	0.13	1.49	0.17	0.5
"Cryptosporidium parvum(cp17)"	"Wuchereria bancrofti (wb123)"	1.32	0.64	2.93	0.47	0.78
"Cryptosporidium parvum(cp17)"	"Diptheria (Dip tox)"	0.93	0.62	1.39	0.72	0.92
"Cryptosporidium parvum(cp17)"	"Measles (wMev)"	1.38	0.92	2.06	0.12	0.42
"Cryptosporidium parvum(cp17)"	"Rubella (wRuv)"	0.73	0.45	1.16	0.19	0.52
"Cryptosporidium parvum(cp17)"	"Tetanus (Tet tox)"	1.08	0.73	1.6	0.69	0.9
"Cryptosporidium parvum(cp23)"	"SARS-CoV-2(sars2np)"	1.69	0.8	3.86	0.19	0.52
"Cryptosporidium parvum(cp23)"	"SARS-CoV-2(sars2rbd)"	0.31	0.1	0.93	0.04	0.24
"Cryptosporidium parvum(cp23)"	"Cryptosporidium parvum(cp17)"	6.53	4.45	9.72	0	0
"Cryptosporidium parvum(cp23)"	"Giardia lamblia(vsp3)"	1.34	0.93	1.94	0.12	0.41
"Cryptosporidium parvum(cp23)"	"Giardia lamblia(vsp5)"	1.08	0.71	1.64	0.72	0.92
"Cryptosporidium parvum(cp23)"	"P. falciparum(csp)"	1.62	1.14	2.31	0.01	0.09
"Cryptosporidium parvum(cp23)"	"P. falciparum(etramp5ag1)"	1.86	1.22	2.88	0	0.06
"Cryptosporidium parvum(cp23)"	"P. falciparum(gexp18)"	1.66	0.8	3.7	0.19	0.52
"Cryptosporidium parvum(cp23)"	"P. falciparum(glurpr2)"	0.66	0.46	0.95	0.03	0.2
"Cryptosporidium parvum(cp23)"	"P. falciparum(pfama1)"	0.7	0.48	1	0.05	0.27
"Cryptosporidium parvum(cp23)"	"P. falciparum(pfmsp119)"	1.39	1.01	1.91	0.04	0.25
"Cryptosporidium parvum(cp23)"	"P. falciparum(rh42)"	1.06	0.65	1.76	0.83	0.96
"Cryptosporidium parvum(cp23)"	"P. malariae(pmmsp119)"	0.92	0.57	1.49	0.72	0.92
"Cryptosporidium parvum(cp23)"	"P. ovale(pomsp119)"	0.98	0.54	1.86	0.96	0.99
"Cryptosporidium parvum(cp23)"	"P. vivax(pvdbprii)"	4.08	1.33	13.43	0.02	0.15
"Cryptosporidium parvum(cp23)"	"P. vivax(pvmsp119)"	0.93	0.33	2.98	0.89	0.97
"Cryptosporidium parvum(cp23)"	"P. vivax(pvrbp2b)"	0.38	0.12	1.14	0.09	0.35
"Cryptosporidium parvum(cp23)"	"Brugia malayi (bm14)"	1.01	0.56	1.88	0.98	1
"Cryptosporidium parvum(cp23)"	"Brugia malayi (bm33)"	0.81	0.57	1.16	0.25	0.61
"Cryptosporidium parvum(cp23)"	"Chlamydia trachomatis (ct694)"	1.11	0.72	1.7	0.64	0.89
"Cryptosporidium parvum(cp23)"	"Chlamydia trachomatis (pgp3)"	1.85	1.19	2.91	0.01	0.08
"Cryptosporidium parvum(cp23)"	"Onchocerca volvulus (ov16)"	2	0.49	11.26	0.38	0.72
"Cryptosporidium parvum(cp23)"	"Schistosoma mansoni (sea)"	1.09	0.78	1.51	0.63	0.88
"Cryptosporidium parvum(cp23)"	"Schistosoma mansoni (sm25)"	1.87	1.31	2.69	0	0.02
"Cryptosporidium parvum(cp23)"	"Strongyloides stercoralis (nie)"	0.89	0.57	1.4	0.61	0.86
"Cryptosporidium parvum(cp23)"	"Taenia solium(es33)"	1.33	0.81	2.24	0.27	0.62
"Cryptosporidium parvum(cp23)"	"Taenia solium(t24h)"	1.57	0.94	2.67	0.09	0.35
"Cryptosporidium parvum(cp23)"	"Treponema palladium (rp17)"	0.87	0.55	1.38	0.54	0.83
"Cryptosporidium parvum(cp23)"	"Treponema palladium (tmpa)"	0.98	0.42	2.5	0.97	0.99
"Cryptosporidium parvum(cp23)"	"Wuchereria bancrofti (wb123)"	0.69	0.45	1.05	0.08	0.33
"Cryptosporidium parvum(cp23)"	"Diptheria (Dip tox)"	1.63	1.19	2.22	0	0.04
"Cryptosporidium parvum(cp23)"	"Measles (wMev)"	1.31	0.96	1.79	0.09	0.35
"Cryptosporidium parvum(cp23)"	"Rubella (wRuv)"	0.68	0.47	0.98	0.04	0.25

"Cryptosporidium parvum(cp23)"	"Tetanus (Tet tox)"	1.18	0.88	1.57	0.27	0.62
"Giardia lamblia(vsp3)"	"SARS-CoV-2(sars2np)"	0.43	0.18	0.95	0.04	0.25
"Giardia lamblia(vsp3)"	"SARS-CoV-2(sars2rbd)"	2.35	0.61	8.95	0.21	0.55
"Giardia lamblia(vsp3)"	"Cryptosporidium parvum(cp17)"	1.22	0.75	2	0.43	0.76
"Giardia lamblia(vsp3)"	"Cryptosporidium parvum(cp23)"	1.39	0.97	2.02	0.08	0.32
"Giardia lamblia(vsp3)"	"Giardia lamblia(vsp5)"	30.34	20	47.25	0	0
"Giardia lamblia(vsp3)"	"P. falciparum(csp)"	1.57	1.09	2.27	0.02	0.15
"Giardia lamblia(vsp3)"	"P. falciparum(etramp5ag1)"	0.81	0.55	1.21	0.31	0.65
"Giardia lamblia(vsp3)"	"P. falciparum(gexp18)"	1.8	0.95	3.4	0.07	0.31
"Giardia lamblia(vsp3)"	"P. falciparum(glurpr2)"	1.37	0.9	2.09	0.14	0.45
"Giardia lamblia(vsp3)"	"P. falciparum(pfama1)"	1.09	0.71	1.68	0.69	0.9
"Giardia lamblia(vsp3)"	"P. falciparum(pfmsp119)"	1.41	1	2	0.05	0.27
"Giardia lamblia(vsp3)"	"P. falciparum(rh42)"	1.61	1	2.59	0.05	0.26
"Giardia lamblia(vsp3)"	"P. malariae(pmmsp119)"	1.24	0.78	1.95	0.36	0.7
"Giardia lamblia(vsp3)"	"P. ovale(pomsp119)"	1.58	0.86	2.92	0.14	0.45
"Giardia lamblia(vsp3)"	"P. vivax(pvdbprii)"	0.88	0.28	2.86	0.84	0.96
"Giardia lamblia(vsp3)"	"P. vivax(pvmsp119)"	1.43	0.58	3.68	0.45	0.77
"Giardia lamblia(vsp3)"	"P. vivax(pvrbp2b)"	1.17	0.36	3.76	0.8	0.95
"Giardia lamblia(vsp3)"	"Brugia malayi (bm14)"	0.73	0.42	1.27	0.27	0.62
"Giardia lamblia(vsp3)"	"Brugia malayi (bm33)"	1.57	1.08	2.27	0.02	0.15
"Giardia lamblia(vsp3)"	"Chlamydia trachomatis (ct694)"	0.87	0.56	1.35	0.54	0.83
"Giardia lamblia(vsp3)"	"Chlamydia trachomatis (pgp3)"	1.18	0.76	1.83	0.46	0.78
"Giardia lamblia(vsp3)"	"Onchocerca volvulus (ov16)"	0.73	0.22	2.38	0.6	0.86
"Giardia lamblia(vsp3)"	"Schistosoma mansoni (sea)"	1.06	0.73	1.56	0.76	0.94
"Giardia lamblia(vsp3)"	"Schistosoma mansoni (sm25)"	1.41	0.93	2.15	0.11	0.39
"Giardia lamblia(vsp3)"	"Strongyloides stercoralis (nie)"	0.92	0.59	1.42	0.7	0.91
"Giardia lamblia(vsp3)"	"Taenia solium(es33)"	0.6	0.36	0.97	0.04	0.24
"Giardia lamblia(vsp3)"	"Taenia solium(t24h)"	1.08	0.68	1.72	0.74	0.93
"Giardia lamblia(vsp3)"	"Treponema palladium (rp17)"	1.46	0.95	2.25	0.09	0.35
"Giardia lamblia(vsp3)"	"Treponema palladium (tmpa)"	1.24	0.54	2.88	0.61	0.86
"Giardia lamblia(vsp3)"	"Wuchereria bancrofti (wb123)"	0.96	0.63	1.44	0.83	0.96
"Giardia lamblia(vsp3)"	"Diptheria (Dip tox)"	0.84	0.59	1.21	0.36	0.71
"Giardia lamblia(vsp3)"	"Measles (wMev)"	0.89	0.63	1.27	0.53	0.82
"Giardia lamblia(vsp3)"	"Rubella (wRuv)"	1.13	0.73	1.75	0.58	0.86
"Giardia lamblia(vsp3)"	"Tetanus (Tet tox)"	0.97	0.71	1.32	0.85	0.96
"Giardia lamblia(vsp5)"	"SARS-CoV-2(sars2np)"	0.64	0.2	1.74	0.41	0.75
"Giardia lamblia(vsp5)"	"SARS-CoV-2(sars2rbd)"	3.34	0.82	13.97	0.09	0.35
"Giardia lamblia(vsp5)"	"Cryptosporidium parvum(cp17)"	1.06	0.6	1.91	0.84	0.96
"Giardia lamblia(vsp5)"	"Cryptosporidium parvum(cp23)"	1.13	0.74	1.73	0.58	0.86
"Giardia lamblia(vsp5)"	"Giardia lamblia(vsp3)"	29.2	19.3	45.37	0	0
"Giardia lamblia(vsp5)"	"P. falciparum(csp)"	0.58	0.38	0.89	0.01	0.13
"Giardia lamblia(vsp5)"	"P. falciparum(etramp5ag1)"	1.28	0.82	1.99	0.28	0.62
"Giardia lamblia(vsp5)"	"P. falciparum(gexp18)"	0.63	0.33	1.2	0.17	0.49
"Giardia lamblia(vsp5)"	"P. falciparum(glurpr2)"	0.54	0.34	0.85	0.01	0.1
"Giardia lamblia(vsp5)"	"P. falciparum(pfama1)"	0.82	0.5	1.32	0.41	0.75
"Giardia lamblia(vsp5)"	"P. falciparum(pfmsp119)"	0.79	0.52	1.18	0.25	0.6

"Giardia lamblia(vsp5)"	"P. falciparum(rh42)"	1.14	0.69	1.88	0.6	0.86
"Giardia lamblia(vsp5)"	"P. malariae(pmmsp119)"	1.21	0.73	2	0.45	0.77
"Giardia lamblia(vsp5)"	"P. ovale(pomsp119)"	1.09	0.59	2.02	0.78	0.95
"Giardia lamblia(vsp5)"	"P. vivax(pvdbprii)"	0.86	0.26	2.75	0.79	0.95
"Giardia lamblia(vsp5)"	"P. vivax(pvmsp119)"	0.93	0.4	2.18	0.87	0.96
"Giardia lamblia(vsp5)"	"P. vivax(pvrbp2b)"	2.7	0.86	8.63	0.09	0.35
"Giardia lamblia(vsp5)"	"Brugia malayi (bm14)"	0.95	0.49	1.79	0.87	0.96
"Giardia lamblia(vsp5)"	"Brugia malayi (bm33)"	0.78	0.51	1.19	0.25	0.61
"Giardia lamblia(vsp5)"	"Chlamydia trachomatis (ct694)"	0.95	0.58	1.56	0.85	0.96
"Giardia lamblia(vsp5)"	"Chlamydia trachomatis (pgp3)"	0.62	0.37	1.02	0.06	0.28
"Giardia lamblia(vsp5)"	"Onchocerca volvulus (ov16)"	1.33	0.4	4.35	0.64	0.88
"Giardia lamblia(vsp5)"	"Schistosoma mansoni (sea)"	0.6	0.39	0.92	0.02	0.17
"Giardia lamblia(vsp5)"	"Schistosoma mansoni (sm25)"	0.9	0.55	1.48	0.68	0.9
"Giardia lamblia(vsp5)"	"Strongyloides stercoralis (nie)"	0.96	0.58	1.56	0.87	0.96
"Giardia lamblia(vsp5)"	"Taenia solium(es33)"	1.73	1.05	2.85	0.03	0.22
"Giardia lamblia(vsp5)"	"Taenia solium(t24h)"	1.14	0.68	1.9	0.61	0.86
"Giardia lamblia(vsp5)"	"Treponema palladium (rp17)"	1.44	0.89	2.34	0.13	0.44
"Giardia lamblia(vsp5)"	"Treponema palladium (tmpa)"	1.34	0.59	2.99	0.48	0.79
"Giardia lamblia(vsp5)"	"Wuchereria bancrofti (wb123)"	1.46	0.93	2.31	0.1	0.36
"Giardia lamblia(vsp5)"	"Diptheria (Dip tox)"	1.15	0.76	1.73	0.51	0.8
"Giardia lamblia(vsp5)"	"Measles (wMev)"	1.63	1.08	2.47	0.02	0.17
"Giardia lamblia(vsp5)"	"Rubella (wRuv)"	0.47	0.29	0.75	0	0.03
"Giardia lamblia(vsp5)"	"Tetanus (Tet tox)"	1.3	0.92	1.86	0.14	0.45
"P. falciparum(csp)"	"SARS-CoV-2(sars2np)"	1.07	0.49	2.29	0.87	0.96
"P. falciparum(csp)"	"SARS-CoV-2(sars2rbd)"	0.54	0.15	1.79	0.32	0.66
"P. falciparum(csp)"	"Cryptosporidium parvum(cp17)"	1.41	0.86	2.34	0.18	0.5
"P. falciparum(csp)"	"Cryptosporidium parvum(cp23)"	1.62	1.14	2.3	0.01	0.08
"P. falciparum(csp)"	"Giardia lamblia(vsp3)"	1.48	1.03	2.12	0.03	0.22
"P. falciparum(csp)"	"Giardia lamblia(vsp5)"	0.64	0.42	0.96	0.03	0.22
"P. falciparum(csp)"	"P. falciparum(etramp5ag1)"	1.09	0.75	1.59	0.64	0.89
"P. falciparum(csp)"	"P. falciparum(gexp18)"	1.12	0.6	2.09	0.73	0.93
"P. falciparum(csp)"	"P. falciparum(glurpr2)"	2.69	1.84	3.94	0	0
"P. falciparum(csp)"	"P. falciparum(pfama1)"	2.62	1.71	4.09	0	0
"P. falciparum(csp)"	"P. falciparum(pfmsp119)"	1.4	1.01	1.93	0.04	0.25
"P. falciparum(csp)"	"P. falciparum(rh42)"	1.16	0.74	1.83	0.51	0.8
"P. falciparum(csp)"	"P. malariae(pmmsp119)"	1.58	1.01	2.48	0.04	0.25
"P. falciparum(csp)"	"P. ovale(pomsp119)"	0.98	0.53	1.81	0.95	0.99
"P. falciparum(csp)"	"P. vivax(pvdbprii)"	0.43	0.14	1.25	0.13	0.43
"P. falciparum(csp)"	"P. vivax(pvmsp119)"	0.89	0.38	2.14	0.8	0.95
"P. falciparum(csp)"	"P. vivax(pvrbp2b)"	1.65	0.56	5.25	0.38	0.72
"P. falciparum(csp)"	"Brugia malayi (bm14)"	5.93	3.08	12.23	0	0
"P. falciparum(csp)"	"Brugia malayi (bm33)"	1.48	1.04	2.1	0.03	0.2
"P. falciparum(csp)"	"Chlamydia trachomatis (ct694)"	1.05	0.7	1.58	0.8	0.96
"P. falciparum(csp)"	"Chlamydia trachomatis (pgp3)"	1.08	0.7	1.64	0.73	0.93
"P. falciparum(csp)"	"Onchocerca volvulus (ov16)"	0.76	0.22	3.12	0.68	0.9
"P. falciparum(csp)"	"Schistosoma mansoni (sea)"	1.62	1.12	2.35	0.01	0.12

"P. falciparum(csp)"	"Schistosoma mansoni (sm25)"	0.99	0.66	1.47	0.95	0.99
"P. falciparum(csp)"	"Strongyloides stercoralis (nie)"	1.18	0.79	1.78	0.42	0.75
"P. falciparum(csp)"	"Taenia solium(es33)"	1.17	0.75	1.83	0.5	0.79
"P. falciparum(csp)"	"Taenia solium(t24h)"	1.02	0.66	1.59	0.93	0.98
"P. falciparum(csp)"	"Treponema palladium (rp17)"	1.16	0.76	1.77	0.5	0.8
"P. falciparum(csp)"	"Treponema palladium (tmpa)"	1.85	0.79	4.64	0.17	0.5
"P. falciparum(csp)"	"Wuchereria bancrofti (wb123)"	1.86	1.25	2.76	0	0.04
"P. falciparum(csp)"	"Diptheria (Dip tox)"	1.36	0.96	1.93	0.09	0.34
"P. falciparum(csp)"	"Measles (wMev)"	1.09	0.78	1.52	0.61	0.86
"P. falciparum(csp)"	"Rubella (wRuv)"	0.91	0.61	1.36	0.64	0.89
"P. falciparum(csp)"	"Tetanus (Tet tox)"	0.97	0.72	1.32	0.85	0.96
"P. falciparum(etramp5ag1)"	"SARS-CoV-2(sars2np)"	1.16	0.51	2.52	0.71	0.92
"P. falciparum(etramp5ag1)"	"SARS-CoV-2(sars2rbd)"	0.88	0.23	2.99	0.84	0.96
"P. falciparum(etramp5ag1)"	"Cryptosporidium parvum(cp17)"	1.26	0.7	2.36	0.45	0.77
"P. falciparum(etramp5ag1)"	"Cryptosporidium parvum(cp23)"	1.86	1.23	2.86	0	0.06
"P. falciparum(etramp5ag1)"	"Giardia lamblia(vsp3)"	0.84	0.57	1.23	0.38	0.72
"P. falciparum(etramp5ag1)"	"Giardia lamblia(vsp5)"	1.35	0.88	2.05	0.16	0.49
"P. falciparum(etramp5ag1)"	"P. falciparum(csp)"	1.08	0.74	1.57	0.69	0.9
"P. falciparum(etramp5ag1)"	"P. falciparum(gexp18)"	2.07	1.17	3.67	0.01	0.13
"P. falciparum(etramp5ag1)"	"P. falciparum(glurpr2)"	2.21	1.38	3.61	0	0.02
"P. falciparum(etramp5ag1)"	"P. falciparum(pfama1)"	1.17	0.71	1.95	0.54	0.83
"P. falciparum(etramp5ag1)"	"P. falciparum(pfmsp119)"	3.29	2.25	4.87	0	0
"P. falciparum(etramp5ag1)"	"P. falciparum(rh42)"	1.29	0.83	2	0.25	0.61
"P. falciparum(etramp5ag1)"	"P. malariae(pmmsp119)"	0.86	0.55	1.33	0.49	0.79
"P. falciparum(etramp5ag1)"	"P. ovale(pomsp119)"	1.58	0.9	2.75	0.1	0.38
"P. falciparum(etramp5ag1)"	"P. vivax(pvdbprii)"	1.13	0.36	3.35	0.82	0.96
"P. falciparum(etramp5ag1)"	"P. vivax(pvmsp119)"	0.66	0.29	1.45	0.3	0.65
"P. falciparum(etramp5ag1)"	"P. vivax(pvrbp2b)"	1	0.34	3.08	1	1
"P. falciparum(etramp5ag1)"	"Brugia malayi (bm14)"	1.06	0.62	1.8	0.82	0.96
"P. falciparum(etramp5ag1)"	"Brugia malayi (bm33)"	1.48	0.99	2.21	0.06	0.27
"P. falciparum(etramp5ag1)"	"Chlamydia trachomatis (ct694)"	2.33	1.51	3.6	0	0
"P. falciparum(etramp5ag1)"	"Chlamydia trachomatis (pgp3)"	0.34	0.21	0.53	0	0
"P. falciparum(etramp5ag1)"	"Onchocerca volvulus (ov16)"	0.88	0.28	2.57	0.82	0.96
"P. falciparum(etramp5ag1)"	"Schistosoma mansoni (sea)"	0.89	0.58	1.36	0.59	0.86
"P. falciparum(etramp5ag1)"	"Schistosoma mansoni (sm25)"	1.05	0.65	1.7	0.86	0.96
"P. falciparum(etramp5ag1)"	"Strongyloides stercoralis (nie)"	1.22	0.8	1.83	0.35	0.7
"P. falciparum(etramp5ag1)"	"Taenia solium(es33)"	0.93	0.58	1.46	0.75	0.93
"P. falciparum(etramp5ag1)"	"Taenia solium(t24h)"	1.51	0.98	2.32	0.06	0.29
"P. falciparum(etramp5ag1)"	"Treponema palladium (rp17)"	1.31	0.86	1.98	0.21	0.54
"P. falciparum(etramp5ag1)"	"Treponema palladium (tmpa)"	0.64	0.29	1.36	0.26	0.61
"P. falciparum(etramp5ag1)"	"Wuchereria bancrofti (wb123)"	1.2	0.79	1.81	0.39	0.73
"P. falciparum(etramp5ag1)"	"Diptheria (Dip tox)"	1.37	0.91	2.08	0.14	0.45
"P. falciparum(etramp5ag1)"	"Measles (wMev)"	0.71	0.49	1.04	0.08	0.33
"P. falciparum(etramp5ag1)"	"Rubella (wRuv)"	1.08	0.67	1.75	0.76	0.94
"P. falciparum(etramp5ag1)"	"Tetanus (Tet tox)"	0.96	0.69	1.33	0.79	0.95
"P. falciparum(gexp18)"	"SARS-CoV-2(sars2np)"	0.82	0.2	2.71	0.76	0.94

"P. falciparum(gexp18)"	"SARS-CoV-2(sars2rbd)"	6.99	1.43	32.63	0.01	0.13
"P. falciparum(gexp18)"	"Cryptosporidium parvum(cp17)"	0.5	0.18	1.64	0.21	0.55
"P. falciparum(gexp18)"	"Cryptosporidium parvum(cp23)"	1.73	0.81	3.97	0.17	0.5
"P. falciparum(gexp18)"	"Giardia lamblia(vsp3)"	1.85	0.98	3.54	0.06	0.28
"P. falciparum(gexp18)"	"Giardia lamblia(vsp5)"	0.75	0.39	1.42	0.38	0.72
"P. falciparum(gexp18)"	"P. falciparum(csp)"	1.07	0.55	2.07	0.85	0.96
"P. falciparum(gexp18)"	"P. falciparum(etramp5ag1)"	2.01	1.12	3.6	0.02	0.16
"P. falciparum(gexp18)"	"P. falciparum(glurpr2)"	0.77	0.35	1.73	0.52	0.81
"P. falciparum(gexp18)"	"P. falciparum(pfama1)"	3.07	1.12	10.05	0.04	0.25
"P. falciparum(gexp18)"	"P. falciparum(pfmsp119)"	0.73	0.38	1.42	0.35	0.7
"P. falciparum(gexp18)"	"P. falciparum(rh42)"	3.6	1.95	6.67	0	0
"P. falciparum(gexp18)"	"P. malariae(pmmsp119)"	1.66	0.87	3.12	0.12	0.41
"P. falciparum(gexp18)"	"P. ovale(pomsp119)"	1.08	0.47	2.33	0.85	0.96
"P. falciparum(gexp18)"	"P. vivax(pvdbprii)"	1.54	0.28	7.38	0.61	0.86
"P. falciparum(gexp18)"	"P. vivax(pvmsp119)"	14.71	6.35	35.52	0	0
"P. falciparum(gexp18)"	"P. vivax(pvrbp2b)"	0.95	0.2	5.01	0.95	0.99
"P. falciparum(gexp18)"	"Brugia malayi (bm14)"	1.47	0.68	3.08	0.32	0.66
"P. falciparum(gexp18)"	"Brugia malayi (bm33)"	1.45	0.7	3.06	0.32	0.66
"P. falciparum(gexp18)"	"Chlamydia trachomatis (ct694)"	1.55	0.76	3.13	0.22	0.57
"P. falciparum(gexp18)"	"Chlamydia trachomatis (pgp3)"	0.83	0.41	1.67	0.6	0.86
"P. falciparum(gexp18)"	"Onchocerca volvulus (ov16)"	1.1	0.22	4.66	0.9	0.98
"P. falciparum(gexp18)"	"Schistosoma mansoni (sea)"	0.66	0.32	1.39	0.26	0.61
"P. falciparum(gexp18)"	"Schistosoma mansoni (sm25)"	1.74	0.65	5.11	0.29	0.63
"P. falciparum(gexp18)"	"Strongyloides stercoralis (nie)"	1.87	1.02	3.4	0.04	0.25
"P. falciparum(gexp18)"	"Taenia solium(es33)"	1.2	0.61	2.29	0.58	0.86
"P. falciparum(gexp18)"	"Taenia solium(t24h)"	1.12	0.58	2.12	0.72	0.92
"P. falciparum(gexp18)"	"Treponema palladium (rp17)"	0.67	0.33	1.3	0.24	0.59
"P. falciparum(gexp18)"	"Treponema palladium (tmpa)"	1.12	0.32	3.35	0.85	0.96
"P. falciparum(gexp18)"	"Wuchereria bancrofti (wb123)"	1.53	0.79	2.94	0.2	0.54
"P. falciparum(gexp18)"	"Diptheria (Dip tox)"	0.84	0.43	1.7	0.63	0.88
"P. falciparum(gexp18)"	"Measles (wMev)"	1.22	0.63	2.42	0.57	0.85
"P. falciparum(gexp18)"	"Rubella (wRuv)"	0.75	0.34	1.71	0.48	0.79
"P. falciparum(gexp18)"	"Tetanus (Tet tox)"	1.15	0.67	2.01	0.61	0.86
"P. falciparum(glurpr2)"	"SARS-CoV-2(sars2np)"	0.78	0.35	1.77	0.56	0.84
"P. falciparum(glurpr2)"	"SARS-CoV-2(sars2rbd)"	1.36	0.39	4.85	0.63	0.88
"P. falciparum(glurpr2)"	"Cryptosporidium parvum(cp17)"	1.33	0.85	2.09	0.22	0.56
"P. falciparum(glurpr2)"	"Cryptosporidium parvum(cp23)"	0.69	0.48	1	0.05	0.27
"P. falciparum(glurpr2)"	"Giardia lamblia(vsp3)"	1.27	0.83	1.96	0.28	0.62
"P. falciparum(glurpr2)"	"Giardia lamblia(vsp5)"	0.55	0.34	0.89	0.01	0.13
"P. falciparum(glurpr2)"	"P. falciparum(csp)"	2.75	1.86	4.09	0	0
"P. falciparum(glurpr2)"	"P. falciparum(etramp5ag1)"	2.3	1.41	3.82	0	0.02
"P. falciparum(glurpr2)"	"P. falciparum(gexp18)"	0.69	0.31	1.58	0.37	0.72
"P. falciparum(glurpr2)"	"P. falciparum(pfama1)"	3.36	2.32	4.89	0	0
"P. falciparum(glurpr2)"	"P. falciparum(pfmsp119)"	2.84	2.02	3.99	0	0
"P. falciparum(glurpr2)"	"P. falciparum(rh42)"	3.66	1.84	7.84	0	0.01
"P. falciparum(glurpr2)"	"P. malariae(pmmsp119)"	1.42	0.78	2.69	0.26	0.61

"P. falciparum(glurpr2)"	"P. ovale(pomsp119)"	1.51	0.74	3.17	0.26	0.61
"P. falciparum(glurpr2)"	"P. vivax(pvdbprii)"	0.93	0.28	3.16	0.91	0.98
"P. falciparum(glurpr2)"	"P. vivax(pvmsp119)"	0.84	0.25	3.28	0.79	0.95
"P. falciparum(glurpr2)"	"P. vivax(pvrbp2b)"	1.09	0.32	3.71	0.89	0.97
"P. falciparum(glurpr2)"	"Brugia malayi (bm14)"	0.93	0.44	2.07	0.86	0.96
"P. falciparum(glurpr2)"	"Brugia malayi (bm33)"	0.94	0.63	1.4	0.77	0.94
"P. falciparum(glurpr2)"	"Chlamydia trachomatis (ct694)"	0.91	0.56	1.49	0.71	0.92
"P. falciparum(glurpr2)"	"Chlamydia trachomatis (pgp3)"	2.22	1.33	3.74	0	0.04
"P. falciparum(glurpr2)"	"Onchocerca volvulus (ov16)"	4.35	0.63	89.52	0.2	0.54
"P. falciparum(glurpr2)"	"Schistosoma mansoni (sea)"	1.25	0.87	1.81	0.22	0.57
"P. falciparum(glurpr2)"	"Schistosoma mansoni (sm25)"	1.97	1.33	2.91	0	0.02
"P. falciparum(glurpr2)"	"Strongyloides stercoralis (nie)"	0.64	0.38	1.08	0.09	0.35
"P. falciparum(glurpr2)"	"Taenia solium(es33)"	0.72	0.42	1.24	0.23	0.58
"P. falciparum(glurpr2)"	"Taenia solium(t24h)"	1.06	0.59	1.91	0.86	0.96
"P. falciparum(glurpr2)"	"Treponema palladium (rp17)"	0.97	0.55	1.73	0.91	0.98
"P. falciparum(glurpr2)"	"Treponema palladium (tmpa)"	1.9	0.61	7.2	0.31	0.65
"P. falciparum(glurpr2)"	"Wuchereria bancrofti (wb123)"	0.85	0.52	1.41	0.54	0.83
"P. falciparum(glurpr2)"	"Diptheria (Dip tox)"	1.54	1.08	2.19	0.02	0.15
"P. falciparum(glurpr2)"	"Measles (wMev)"	0.96	0.67	1.36	0.8	0.96
"P. falciparum(glurpr2)"	"Rubella (wRuv)"	1.06	0.71	1.57	0.79	0.95
"P. falciparum(glurpr2)"	"Tetanus (Tet tox)"	1.03	0.74	1.43	0.88	0.96
"P. falciparum(pfama1)"	"SARS-CoV-2(sars2np)"	0.23	0.1	0.52	0	0.01
"P. falciparum(pfama1)"	"SARS-CoV-2(sars2rbd)"	2.74	0.81	9.77	0.11	0.4
"P. falciparum(pfama1)"	"Cryptosporidium parvum(cp17)"	1.13	0.72	1.76	0.59	0.86
"P. falciparum(pfama1)"	"Cryptosporidium parvum(cp23)"	0.6	0.4	0.88	0.01	0.1
"P. falciparum(pfama1)"	"Giardia lamblia(vsp3)"	1	0.63	1.61	1	1
"P. falciparum(pfama1)"	"Giardia lamblia(vsp5)"	0.76	0.46	1.25	0.28	0.62
"P. falciparum(pfama1)"	"P. falciparum(csp)"	2.27	1.43	3.66	0	0.01
"P. falciparum(pfama1)"	"P. falciparum(etramp5ag1)"	1.08	0.63	1.87	0.79	0.95
"P. falciparum(pfama1)"	"P. falciparum(gexp18)"	4.19	1.41	14.78	0.02	0.14
"P. falciparum(pfama1)"	"P. falciparum(glurpr2)"	3.18	2.18	4.68	0	0
"P. falciparum(pfama1)"	"P. falciparum(pfmsp119)"	1.65	1.14	2.4	0.01	0.09
"P. falciparum(pfama1)"	"P. falciparum(rh42)"	2.19	1.03	5.16	0.05	0.27
"P. falciparum(pfama1)"	"P. malariae(pmmsp119)"	0.79	0.41	1.59	0.49	0.79
"P. falciparum(pfama1)"	"P. ovale(pomsp119)"	0.37	0.18	0.76	0.01	0.08
"P. falciparum(pfama1)"	"P. vivax(pvdbprii)"	5.21	1.43	20.24	0.01	0.13
"P. falciparum(pfama1)"	"P. vivax(pvmsp119)"	0.16	0.05	0.65	0.01	0.07
"P. falciparum(pfama1)"	"P. vivax(pvrbp2b)"	0.61	0.17	2.24	0.45	0.77
"P. falciparum(pfama1)"	"Brugia malayi (bm14)"	0.48	0.22	1.1	0.07	0.31
"P. falciparum(pfama1)"	"Brugia malayi (bm33)"	1.1	0.72	1.67	0.66	0.89
"P. falciparum(pfama1)"	"Chlamydia trachomatis (ct694)"	1.4	0.81	2.45	0.23	0.58
"P. falciparum(pfama1)"	"Chlamydia trachomatis (pgp3)"	0.67	0.39	1.17	0.16	0.48
"P. falciparum(pfama1)"	"Onchocerca volvulus (ov16)"	1.6	0.26	31.15	0.67	0.9
"P. falciparum(pfama1)"	"Schistosoma mansoni (sea)"	1.44	1.01	2.06	0.04	0.25
"P. falciparum(pfama1)"	"Schistosoma mansoni (sm25)"	1.2	0.79	1.82	0.39	0.72
"P. falciparum(pfama1)"	"Strongyloides stercoralis (nie)"	0.68	0.39	1.21	0.18	0.51

"P. falciparum(pfama1)"	"Taenia solium(es33)"	2.03	1.03	4.21	0.05	0.26
"P. falciparum(pfama1)"	"Taenia solium(t24h)"	1.3	0.69	2.54	0.43	0.76
"P. falciparum(pfama1)"	"Treponema palladium (rp17)"	0.83	0.44	1.63	0.58	0.86
"P. falciparum(pfama1)"	"Treponema palladium (tmpa)"	4.04	0.84	33.01	0.13	0.42
"P. falciparum(pfama1)"	"Wuchereria bancrofti (wb123)"	0.75	0.42	1.33	0.31	0.66
"P. falciparum(pfama1)"	"Diptheria (Dip tox)"	0.81	0.57	1.16	0.25	0.61
"P. falciparum(pfama1)"	"Measles (wMev)"	0.7	0.48	1.01	0.06	0.27
"P. falciparum(pfama1)"	"Rubella (wRuv)"	0.83	0.55	1.25	0.38	0.72
"P. falciparum(pfama1)"	"Tetanus (Tet tox)"	0.84	0.6	1.17	0.3	0.65
"P. falciparum(pfmsp119)"	"SARS-CoV-2(sars2np)"	0.92	0.44	1.92	0.83	0.96
"P. falciparum(pfmsp119)"	"SARS-CoV-2(sars2rbd)"	1.4	0.47	4.21	0.55	0.84
"P. falciparum(pfmsp119)"	"Cryptosporidium parvum(cp17)"	0.53	0.35	0.79	0	0.04
"P. falciparum(pfmsp119)"	"Cryptosporidium parvum(cp23)"	1.36	0.99	1.88	0.06	0.27
"P. falciparum(pfmsp119)"	"Giardia lamblia(vsp3)"	1.44	1.03	2.04	0.04	0.24
"P. falciparum(pfmsp119)"	"Giardia lamblia(vsp5)"	0.73	0.5	1.08	0.12	0.41
"P. falciparum(pfmsp119)"	"P. falciparum(csp)"	1.43	1.04	1.97	0.03	0.21
"P. falciparum(pfmsp119)"	"P. falciparum(etramp5ag1)"	3.36	2.3	4.96	0	0
"P. falciparum(pfmsp119)"	"P. falciparum(gexp18)"	0.77	0.41	1.46	0.42	0.75
"P. falciparum(pfmsp119)"	"P. falciparum(glurpr2)"	2.65	1.91	3.69	0	0
"P. falciparum(pfmsp119)"	"P. falciparum(pfama1)"	1.55	1.08	2.22	0.02	0.15
"P. falciparum(pfmsp119)"	"P. falciparum(rh42)"	2.06	1.3	3.31	0	0.04
"P. falciparum(pfmsp119)"	"P. malariae(pmmsp119)"	1.65	1.05	2.63	0.03	0.23
"P. falciparum(pfmsp119)"	"P. ovale(pomsp119)"	1.14	0.64	2.05	0.66	0.89
"P. falciparum(pfmsp119)"	"P. vivax(pvdbprii)"	1.19	0.43	3.29	0.74	0.93
"P. falciparum(pfmsp119)"	"P. vivax(pvmsp119)"	0.64	0.27	1.51	0.3	0.64
"P. falciparum(pfmsp119)"	"P. vivax(pvrbp2b)"	1.06	0.38	2.96	0.91	0.98
"P. falciparum(pfmsp119)"	"Brugia malayi (bm14)"	0.85	0.5	1.47	0.56	0.85
"P. falciparum(pfmsp119)"	"Brugia malayi (bm33)"	0.88	0.63	1.23	0.46	0.78
"P. falciparum(pfmsp119)"	"Chlamydia trachomatis (ct694)"	1.33	0.89	1.98	0.16	0.47
"P. falciparum(pfmsp119)"	"Chlamydia trachomatis (pgp3)"	0.88	0.59	1.33	0.55	0.84
"P. falciparum(pfmsp119)"	"Onchocerca volvulus (ov16)"	0.98	0.31	3.58	0.98	1
"P. falciparum(pfmsp119)"	"Schistosoma mansoni (sea)"	0.74	0.53	1.03	0.08	0.33
"P. falciparum(pfmsp119)"	"Schistosoma mansoni (sm25)"	1.22	0.86	1.75	0.27	0.62
"P. falciparum(pfmsp119)"	"Strongyloides stercoralis (nie)"	1.55	1.03	2.35	0.04	0.24
"P. falciparum(pfmsp119)"	"Taenia solium(es33)"	0.92	0.59	1.42	0.7	0.91
"P. falciparum(pfmsp119)"	"Taenia solium(t24h)"	0.66	0.43	1.03	0.07	0.3
"P. falciparum(pfmsp119)"	"Treponema palladium (rp17)"	1.97	1.27	3.08	0	0.04
"P. falciparum(pfmsp119)"	"Treponema palladium (tmpa)"	1.21	0.55	2.82	0.64	0.89
"P. falciparum(pfmsp119)"	"Wuchereria bancrofti (wb123)"	1.06	0.72	1.58	0.75	0.94
"P. falciparum(pfmsp119)"	"Diptheria (Dip tox)"	1.06	0.77	1.45	0.73	0.93
"P. falciparum(pfmsp119)"	"Measles (wMev)"	1.12	0.83	1.53	0.45	0.77
"P. falciparum(pfmsp119)"	"Rubella (wRuv)"	1.42	0.99	2.05	0.06	0.27
"P. falciparum(pfmsp119)"	"Tetanus (Tet tox)"	1.18	0.89	1.57	0.24	0.59
"P. falciparum(rh42)"	"SARS-CoV-2(sars2np)"	4.22	1.75	9.96	0	0.02
"P. falciparum(rh42)"	"SARS-CoV-2(sars2rbd)"	0.22	0.04	1.04	0.08	0.32
"P. falciparum(rh42)"	"Cryptosporidium parvum(cp17)"	4.27	1.46	18.31	0.02	0.17

"P. falciparum(rh42)"	"Cryptosporidium parvum(cp23)"	0.98	0.59	1.65	0.94	0.99
"P. falciparum(rh42)"	"Giardia lamblia(vsp3)"	1.67	1.07	2.64	0.03	0.2
"P. falciparum(rh42)"	"Giardia lamblia(vsp5)"	1.28	0.79	2.07	0.31	0.66
"P. falciparum(rh42)"	"P. falciparum(csp)"	1.18	0.75	1.86	0.48	0.79
"P. falciparum(rh42)"	"P. falciparum(etramp5ag1)"	1.41	0.91	2.17	0.12	0.42
"P. falciparum(rh42)"	"P. falciparum(gexp18)"	3.34	1.85	6.06	0	0
"P. falciparum(rh42)"	"P. falciparum(glurpr2)"	3.11	1.59	6.55	0	0.03
"P. falciparum(rh42)"	"P. falciparum(pfama1)"	2.3	1.07	5.44	0.04	0.25
"P. falciparum(rh42)"	"P. falciparum(pfmsp119)"	2.06	1.27	3.38	0	0.05
"P. falciparum(rh42)"	"P. malariae(pmmsp119)"	1.36	0.83	2.2	0.22	0.56
"P. falciparum(rh42)"	"P. ovale(pomsp119)"	1.27	0.65	2.41	0.48	0.79
"P. falciparum(rh42)"	"P. vivax(pvdbprii)"	0.88	0.22	3.31	0.86	0.96
"P. falciparum(rh42)"	"P. vivax(pvmsp119)"	1.29	0.57	2.88	0.54	0.83
"P. falciparum(rh42)"	"P. vivax(pvrbp2b)"	1.38	0.38	5.44	0.63	0.88
"P. falciparum(rh42)"	"Brugia malayi (bm14)"	1.57	0.88	2.77	0.12	0.42
"P. falciparum(rh42)"	"Brugia malayi (bm33)"	1.31	0.78	2.19	0.31	0.65
"P. falciparum(rh42)"	"Chlamydia trachomatis (ct694)"	0.95	0.57	1.59	0.85	0.96
"P. falciparum(rh42)"	"Chlamydia trachomatis (pgp3)"	0.99	0.59	1.65	0.96	0.99
"P. falciparum(rh42)"	"Onchocerca volvulus (ov16)"	0.47	0.13	1.53	0.23	0.58
"P. falciparum(rh42)"	"Schistosoma mansoni (sea)"	1.29	0.73	2.35	0.4	0.74
"P. falciparum(rh42)"	"Schistosoma mansoni (sm25)"	1.15	0.59	2.37	0.69	0.9
"P. falciparum(rh42)"	"Strongyloides stercoralis (nie)"	1.36	0.85	2.16	0.19	0.53
"P. falciparum(rh42)"	"Taenia solium(es33)"	0.81	0.47	1.37	0.45	0.77
"P. falciparum(rh42)"	"Taenia solium(t24h)"	1.53	0.94	2.47	0.09	0.35
"P. falciparum(rh42)"	"Treponema palladium (rp17)"	1.18	0.73	1.9	0.49	0.79
"P. falciparum(rh42)"	"Treponema palladium (tmpa)"	1.71	0.77	3.72	0.18	0.51
"P. falciparum(rh42)"	"Wuchereria bancrofti (wb123)"	0.99	0.61	1.58	0.96	0.99
"P. falciparum(rh42)"	"Diptheria (Dip tox)"	0.58	0.35	0.94	0.03	0.2
"P. falciparum(rh42)"	"Measles (wMev)"	1.08	0.67	1.77	0.75	0.93
"P. falciparum(rh42)"	"Rubella (wRuv)"	1.87	0.97	3.77	0.07	0.31
"P. falciparum(rh42)"	"Tetanus (Tet tox)"	0.61	0.41	0.92	0.02	0.16
"P. malariae(pmmsp119)"	"SARS-CoV-2(sars2np)"	1.35	0.52	3.23	0.52	0.81
"P. malariae(pmmsp119)"	"SARS-CoV-2(sars2rbd)"	1.61	0.38	6.04	0.5	0.79
"P. malariae(pmmsp119)"	"Cryptosporidium parvum(cp17)"	1.79	0.79	4.71	0.2	0.53
"P. malariae(pmmsp119)"	"Cryptosporidium parvum(cp23)"	0.84	0.52	1.38	0.49	0.79
"P. malariae(pmmsp119)"	"Giardia lamblia(vsp3)"	1.17	0.75	1.82	0.49	0.79
"P. malariae(pmmsp119)"	"Giardia lamblia(vsp5)"	1.07	0.66	1.75	0.77	0.95
"P. malariae(pmmsp119)"	"P. falciparum(csp)"	1.59	1.02	2.51	0.04	0.25
"P. malariae(pmmsp119)"	"P. falciparum(etramp5ag1)"	0.91	0.58	1.41	0.68	0.9
"P. malariae(pmmsp119)"	"P. falciparum(gexp18)"	1.45	0.76	2.72	0.25	0.61
"P. malariae(pmmsp119)"	"P. falciparum(glurpr2)"	1.52	0.84	2.84	0.18	0.5
"P. malariae(pmmsp119)"	"P. falciparum(pfama1)"	0.93	0.5	1.83	0.84	0.96
"P. malariae(pmmsp119)"	"P. falciparum(pfmsp119)"	1.71	1.07	2.75	0.03	0.2
"P. malariae(pmmsp119)"	"P. falciparum(rh42)"	1.29	0.79	2.1	0.31	0.65
"P. malariae(pmmsp119)"	"P. ovale(pomsp119)"	2.67	1.51	4.69	0	0.02
"P. malariae(pmmsp119)"	"P. vivax(pvdbprii)"	0.21	0.05	0.84	0.03	0.22

"P. malariae(pmmsp119)"	"P. vivax(pvmsp119)"	0.84	0.35	1.91	0.68	0.9
"P. malariae(pmmsp119)"	"P. vivax(pvrbp2b)"	2.13	0.57	8.36	0.27	0.62
"P. malariae(pmmsp119)"	"Brugia malayi (bm14)"	0.94	0.52	1.66	0.84	0.96
"P. malariae(pmmsp119)"	"Brugia malayi (bm33)"	2.06	1.25	3.44	0.01	0.07
"P. malariae(pmmsp119)"	"Chlamydia trachomatis (ct694)"	1.85	1.14	3.03	0.01	0.13
"P. malariae(pmmsp119)"	"Chlamydia trachomatis (pgp3)"	0.56	0.34	0.93	0.03	0.2
"P. malariae(pmmsp119)"	"Onchocerca volvulus (ov16)"	1.66	0.55	4.84	0.36	0.7
"P. malariae(pmmsp119)"	"Schistosoma mansoni (sea)"	2.13	1.19	4.03	0.01	0.13
"P. malariae(pmmsp119)"	"Schistosoma mansoni (sm25)"	0.96	0.51	1.86	0.91	0.98
"P. malariae(pmmsp119)"	"Strongyloides stercoralis (nie)"	1.19	0.75	1.86	0.46	0.78
"P. malariae(pmmsp119)"	"Taenia solium(es33)"	1.03	0.62	1.68	0.92	0.98
"P. malariae(pmmsp119)"	"Taenia solium(t24h)"	0.78	0.46	1.28	0.33	0.67
"P. malariae(pmmsp119)"	"Treponema palladium (rp17)"	1.33	0.84	2.09	0.22	0.56
"P. malariae(pmmsp119)"	"Treponema palladium (tmpa)"	0.68	0.28	1.52	0.36	0.71
"P. malariae(pmmsp119)"	"Wuchereria bancrofti (wb123)"	0.99	0.62	1.57	0.97	1
"P. malariae(pmmsp119)"	"Diptheria (Dip tox)"	1.38	0.83	2.34	0.23	0.57
"P. malariae(pmmsp119)"	"Measles (wMev)"	1.22	0.76	1.98	0.41	0.75
"P. malariae(pmmsp119)"	"Rubella (wRuv)"	1.01	0.56	1.87	0.97	1
"P. malariae(pmmsp119)"	"Tetanus (Tet tox)"	1	0.68	1.48	0.99	1
"P. ovale(pomsp119)"	"SARS-CoV-2(sars2np)"	1.53	0.51	4.05	0.42	0.75
"P. ovale(pomsp119)"	"SARS-CoV-2(sars2rbd)"	1.72	0.37	7.17	0.47	0.78
"P. ovale(pomsp119)"	"Cryptosporidium parvum(cp17)"	0.97	0.39	2.8	0.96	0.99
"P. ovale(pomsp119)"	"Cryptosporidium parvum(cp23)"	0.95	0.51	1.84	0.89	0.97
"P. ovale(pomsp119)"	"Giardia lamblia(vsp3)"	1.5	0.83	2.73	0.18	0.51
"P. ovale(pomsp119)"	"Giardia lamblia(vsp5)"	1.28	0.7	2.32	0.42	0.75
"P. ovale(pomsp119)"	"P. falciparum(csp)"	1	0.55	1.82	0.99	1
"P. ovale(pomsp119)"	"P. falciparum(etramp5ag1)"	1.78	1.02	3.06	0.04	0.24
"P. ovale(pomsp119)"	"P. falciparum(gexp18)"	1.05	0.47	2.23	0.91	0.98
"P. ovale(pomsp119)"	"P. falciparum(glurpr2)"	1.27	0.63	2.63	0.51	0.8
"P. ovale(pomsp119)"	"P. falciparum(pfama1)"	0.4	0.2	0.8	0.01	0.1
"P. ovale(pomsp119)"	"P. falciparum(pfmsp119)"	0.94	0.53	1.7	0.84	0.96
"P. ovale(pomsp119)"	"P. falciparum(rh42)"	1.4	0.74	2.61	0.3	0.64
"P. ovale(pomsp119)"	"P. malariae(pmmsp119)"	2.76	1.57	4.82	0	0.01
"P. ovale(pomsp119)"	"P. vivax(pvdbprii)"	1.76	0.4	7.36	0.45	0.77
"P. ovale(pomsp119)"	"P. vivax(pvmsp119)"	0.84	0.28	2.28	0.74	0.93
"P. ovale(pomsp119)"	"P. vivax(pvrbp2b)"	0.78	0.19	3.24	0.73	0.93
"P. ovale(pomsp119)"	"Brugia malayi (bm14)"	0.82	0.35	1.77	0.63	0.88
"P. ovale(pomsp119)"	"Brugia malayi (bm33)"	2.47	1.29	4.84	0.01	0.08
"P. ovale(pomsp119)"	"Chlamydia trachomatis (ct694)"	1.66	0.88	3.13	0.12	0.41
"P. ovale(pomsp119)"	"Chlamydia trachomatis (pgp3)"	0.56	0.29	1.07	0.08	0.34
"P. ovale(pomsp119)"	"Onchocerca volvulus (ov16)"	2.01	0.5	6.74	0.28	0.63
"P. ovale(pomsp119)"	"Schistosoma mansoni (sea)"	1.11	0.58	2.24	0.76	0.94
"P. ovale(pomsp119)"	"Schistosoma mansoni (sm25)"	1.37	0.63	3.07	0.43	0.76
"P. ovale(pomsp119)"	"Strongyloides stercoralis (nie)"	0.97	0.52	1.74	0.91	0.98
"P. ovale(pomsp119)"	"Taenia solium(es33)"	1.25	0.66	2.31	0.47	0.78
"P. ovale(pomsp119)"	"Taenia solium(t24h)"	0.88	0.46	1.64	0.7	0.91

"P. ovale(pomsp119)"	"Treponema palladium (rp17)"	0.7	0.37	1.29	0.26	0.62
"P. ovale(pomsp119)"	"Treponema palladium (tmpa)"	1.03	0.29	3.02	0.96	0.99
"P. ovale(pomsp119)"	"Wuchereria bancrofti (wb123)"	1.03	0.57	1.84	0.93	0.98
"P. ovale(pomsp119)"	"Diptheria (Dip tox)"	0.64	0.36	1.18	0.14	0.45
"P. ovale(pomsp119)"	"Measles (wMev)"	2.11	1.13	4.19	0.02	0.19
"P. ovale(pomsp119)"	"Rubella (wRuv)"	0.7	0.35	1.46	0.32	0.66
"P. ovale(pomsp119)"	"Tetanus (Tet tox)"	1.62	0.99	2.71	0.06	0.28
"P. vivax(pvdbprii)"	"SARS-CoV-2(sars2np)"	1.57	0	2351.93	0.92	0.98
"P. vivax(pvdbprii)"	"SARS-CoV-2(sars2rbd)"	17.48	0	4714116.77	0.61	0.86
"P. vivax(pvdbprii)"	"Cryptosporidium parvum(cp17)"	0.01	0	0.81	0.06	0.29
"P. vivax(pvdbprii)"	"Cryptosporidium parvum(cp23)"	2218.23	53.88	944629.78	0	0.02
"P. vivax(pvdbprii)"	"Giardia lamblia(vsp3)"	1.98	0.18	26.77	0.58	0.86
"P. vivax(pvdbprii)"	"Giardia lamblia(vsp5)"	0.98	0.08	11.4	0.99	1
"P. vivax(pvdbprii)"	"P. falciparum(csp)"	0.01	0	0.15	0.01	0.08
"P. vivax(pvdbprii)"	"P. falciparum(etramp5ag1)"	1.97	0.15	32.06	0.6	0.86
"P. vivax(pvdbprii)"	"P. falciparum(gexp18)"	6.61	0.07	372.41	0.36	0.7
"P. vivax(pvdbprii)"	"P. falciparum(glurpr2)"	0.24	0.01	5.96	0.39	0.73
"P. vivax(pvdbprii)"	"P. falciparum(pfama1)"	268.92	8.23	82838.31	0.01	0.12
"P. vivax(pvdbprii)"	"P. falciparum(pfmsp119)"	0.42	0.03	4.24	0.46	0.78
"P. vivax(pvdbprii)"	"P. falciparum(rh42)"	3.47	0.16	98.6	0.44	0.76
"P. vivax(pvdbprii)"	"P. malariae(pmmsp119)"	0.01	0	0.53	0.04	0.25
"P. vivax(pvdbprii)"	"P. ovale(pomsp119)"	117.36	1.24	72337.53	0.07	0.31
"P. vivax(pvdbprii)"	"P. vivax(pvmsp119)"	0	0	0.73	0.06	0.28
"P. vivax(pvdbprii)"	"P. vivax(pvrbp2b)"	5345.03	179.32	1916895.34	0	0
"P. vivax(pvdbprii)"	"Brugia malayi (bm14)"	0	0	0.19	0.04	0.24
"P. vivax(pvdbprii)"	"Brugia malayi (bm33)"	1.49	0.13	21.63	0.75	0.93
"P. vivax(pvdbprii)"	"Chlamydia trachomatis (ct694)"	2.09	0.16	40.31	0.59	0.86
"P. vivax(pvdbprii)"	"Chlamydia trachomatis (pgp3)"	0.03	0	0.75	0.06	0.27
"P. vivax(pvdbprii)"	"Onchocerca volvulus (ov16)"	0.02	0	629.92	0.91	0.98
"P. vivax(pvdbprii)"	"Schistosoma mansoni (sea)"	0.95	0.06	14	0.97	1
"P. vivax(pvdbprii)"	"Schistosoma mansoni (sm25)"	0.01	0	0.31	0.02	0.19
"P. vivax(pvdbprii)"	"Strongyloides stercoralis (nie)"	0.14	0.01	2.51	0.18	0.51
"P. vivax(pvdbprii)"	"Taenia solium(es33)"	0.54	0.03	6.58	0.64	0.89
"P. vivax(pvdbprii)"	"Taenia solium(t24h)"	0	0	0.09	0	0.04
"P. vivax(pvdbprii)"	"Treponema palladium (rp17)"	0.27	0.01	4.14	0.39	0.73
"P. vivax(pvdbprii)"	"Treponema palladium (tmpa)"	0.19	0	14752.73	0.8	0.96
"P. vivax(pvdbprii)"	"Wuchereria bancrofti (wb123)"	8.38	0.31	708.78	0.25	0.61
"P. vivax(pvdbprii)"	"Diptheria (Dip tox)"	4.35	0.26	168.39	0.36	0.7
"P. vivax(pvdbprii)"	"Measles (wMev)"	0.23	0.01	2.34	0.24	0.59
"P. vivax(pvdbprii)"	"Rubella (wRuv)"	412.89	4.61	482410.46	0.03	0.23
"P. vivax(pvdbprii)"	"Tetanus (Tet tox)"	3.23	0.26	64.76	0.39	0.72
"P. vivax(pvmsp119)"	"SARS-CoV-2(sars2np)"	0.17	0	4.14	0.42	0.75
"P. vivax(pvmsp119)"	"SARS-CoV-2(sars2rbd)"	15.03	0.32	743.72	0.22	0.57
"P. vivax(pvmsp119)"	"Cryptosporidium parvum(cp17)"	326453520727.22	0	1.86114660823163e+184	0.98	1
"P. vivax(pvmsp119)"	"Cryptosporidium parvum(cp23)"	0.95	0.28	3.82	0.94	0.99
"P. vivax(pvmsp119)"	"Giardia lamblia(vsp3)"	1.6	0.61	4.4	0.34	0.69

"P. vivax(pvmsp119)"	"Giardia lamblia(vsp5)"	1.1	0.41	2.93	0.85	0.96
"P. vivax(pvmsp119)"	"P. falciparum(csp)"	2.09	0.74	6.46	0.18	0.51
"P. vivax(pvmsp119)"	"P. falciparum(etramp5ag1)"	0.84	0.32	2.13	0.72	0.92
"P. vivax(pvmsp119)"	"P. falciparum(gexp18)"	18.75	7.15	53.52	0	0
"P. vivax(pvmsp119)"	"P. falciparum(glurpr2)"	0.74	0.21	2.92	0.65	0.89
"P. vivax(pvmsp119)"	"P. falciparum(pfama1)"	0.34	0.07	1.94	0.2	0.53
"P. vivax(pvmsp119)"	"P. falciparum(pfmsp119)"	1.26	0.44	3.65	0.67	0.9
"P. vivax(pvmsp119)"	"P. falciparum(rh42)"	0.91	0.32	2.47	0.85	0.96
"P. vivax(pvmsp119)"	"P. malariae(pmmsp119)"	0.49	0.15	1.42	0.2	0.54
"P. vivax(pvmsp119)"	"P. ovale(pomsp119)"	0.96	0.25	3.25	0.94	0.99
"P. vivax(pvmsp119)"	"P. vivax(pvdbprii)"	0.35	0.03	3.44	0.4	0.74
"P. vivax(pvmsp119)"	"P. vivax(pvrbp2b)"	0.93	0.1	9.7	0.95	0.99
"P. vivax(pvmsp119)"	"Brugia malayi (bm14)"	0.34	0.1	1.05	0.07	0.32
"P. vivax(pvmsp119)"	"Brugia malayi (bm33)"	0.99	0.27	4.11	0.99	1
"P. vivax(pvmsp119)"	"Chlamydia trachomatis (ct694)"	0.55	0.19	1.6	0.28	0.62
"P. vivax(pvmsp119)"	"Chlamydia trachomatis (pgp3)"	1.26	0.45	3.45	0.66	0.89
"P. vivax(pvmsp119)"	"Onchocerca volvulus (ov16)"	5.92	0.54	60.85	0.14	0.45
"P. vivax(pvmsp119)"	"Schistosoma mansoni (sea)"	0.96	0.26	4.22	0.95	0.99
"P. vivax(pvmsp119)"	"Schistosoma mansoni (sm25)"	1.68	0.2	39.36	0.67	0.9
"P. vivax(pvmsp119)"	"Strongyloides stercoralis (nie)"	1.23	0.49	3	0.65	0.89
"P. vivax(pvmsp119)"	"Taenia solium(es33)"	0.48	0.15	1.38	0.19	0.52
"P. vivax(pvmsp119)"	"Taenia solium(t24h)"	6.19	2.36	17.62	0	0.01
"P. vivax(pvmsp119)"	"Treponema palladium (rp17)"	0.91	0.33	2.38	0.84	0.96
"P. vivax(pvmsp119)"	"Treponema palladium (tmpa)"	0.23	0.02	1.39	0.15	0.45
"P. vivax(pvmsp119)"	"Wuchereria bancrofti (wb123)"	0.87	0.31	2.51	0.79	0.95
"P. vivax(pvmsp119)"	"Diptheria (Dip tox)"	1.91	0.58	7.77	0.32	0.66
"P. vivax(pvmsp119)"	"Measles (wMev)"	1.1	0.38	3.47	0.87	0.96
"P. vivax(pvmsp119)"	"Rubella (wRuv)"	3.26	0.8	16.53	0.12	0.42
"P. vivax(pvmsp119)"	"Tetanus (Tet tox)"	1.46	0.63	3.47	0.38	0.72
"P. vivax(pvrbp2b)"	"SARS-CoV-2(sars2np)"	0.51	0.01	14.33	0.74	0.93
"P. vivax(pvrbp2b)"	"SARS-CoV-2(sars2rbd)"	1.3	0.01	127.59	0.92	0.98
"P. vivax(pvrbp2b)"	"Cryptosporidium parvum(cp17)"	0.63	0.07	6.12	0.68	0.9
"P. vivax(pvrbp2b)"	"Cryptosporidium parvum(cp23)"	0.21	0.05	0.87	0.04	0.23
"P. vivax(pvrbp2b)"	"Giardia lamblia(vsp3)"	0.83	0.2	3.5	0.8	0.96
"P. vivax(pvrbp2b)"	"Giardia lamblia(vsp5)"	2.88	0.76	11.16	0.12	0.41
"P. vivax(pvrbp2b)"	"P. falciparum(csp)"	1.02	0.26	4.15	0.97	1
"P. vivax(pvrbp2b)"	"P. falciparum(etramp5ag1)"	1.23	0.36	4.33	0.75	0.93
"P. vivax(pvrbp2b)"	"P. falciparum(gexp18)"	1.22	0.19	7.25	0.83	0.96
"P. vivax(pvrbp2b)"	"P. falciparum(glurpr2)"	0.85	0.19	3.59	0.82	0.96
"P. vivax(pvrbp2b)"	"P. falciparum(pfama1)"	0.5	0.1	2.54	0.41	0.74
"P. vivax(pvrbp2b)"	"P. falciparum(pfmsp119)"	1.2	0.34	4.32	0.78	0.95
"P. vivax(pvrbp2b)"	"P. falciparum(rh42)"	4.29	0.93	21.19	0.07	0.29
"P. vivax(pvrbp2b)"	"P. malariae(pmmsp119)"	1.65	0.3	9.3	0.57	0.85
"P. vivax(pvrbp2b)"	"P. ovale(pomsp119)"	0.41	0.08	2	0.28	0.62
"P. vivax(pvrbp2b)"	"P. vivax(pvdbprii)"	161.79	33.91	1100.03	0	0
"P. vivax(pvrbp2b)"	"P. vivax(pvmsp119)"	1.84	0.24	14.47	0.56	0.84

"P. vivax(pvrbp2b)"	"Brugia malayi (bm14)"	0.47	0.05	3.59	0.5	0.79
"P. vivax(pvrbp2b)"	"Brugia malayi (bm33)"	0.41	0.11	1.48	0.19	0.52
"P. vivax(pvrbp2b)"	"Chlamydia trachomatis (ct694)"	0.23	0.05	0.97	0.05	0.27
"P. vivax(pvrbp2b)"	"Chlamydia trachomatis (pgp3)"	3.79	0.87	17.79	0.08	0.33
"P. vivax(pvrbp2b)"	"Onchocerca volvulus (ov16)"	0.07	0	15.6	0.57	0.85
"P. vivax(pvrbp2b)"	"Schistosoma mansoni (sea)"	2.39	0.52	12.29	0.28	0.62
"P. vivax(pvrbp2b)"	"Schistosoma mansoni (sm25)"	0.41	0.09	1.72	0.23	0.58
"P. vivax(pvrbp2b)"	"Strongyloides stercoralis (nie)"	8.48	1.6	54.29	0.02	0.15
"P. vivax(pvrbp2b)"	"Taenia solium(es33)"	1.74	0.4	7.4	0.45	0.77
"P. vivax(pvrbp2b)"	"Taenia solium(t24h)"	0.73	0.18	2.89	0.65	0.89
"P. vivax(pvrbp2b)"	"Treponema palladium (rp17)"	2.22	0.59	8.6	0.24	0.59
"P. vivax(pvrbp2b)"	"Treponema palladium (tmpa)"	0.01	0	0.75	0.04	0.24
"P. vivax(pvrbp2b)"	"Wuchereria bancrofti (wb123)"	0.96	0.2	4.74	0.96	0.99
"P. vivax(pvrbp2b)"	"Diptheria (Dip tox)"	1.22	0.26	6.14	0.8	0.96
"P. vivax(pvrbp2b)"	"Measles (wMev)"	0.51	0.13	1.97	0.33	0.67
"P. vivax(pvrbp2b)"	"Rubella (wRuv)"	0.32	0.06	1.66	0.18	0.51
"P. vivax(pvrbp2b)"	"Tetanus (Tet tox)"	1.32	0.4	4.51	0.65	0.89
"Brugia malayi (bm14)"	"SARS-CoV-2(sars2np)"	1.09	0.35	2.96	0.87	0.96
"Brugia malayi (bm14)"	"SARS-CoV-2(sars2rbd)"	0.69	0.03	4.97	0.75	0.93
"Brugia malayi (bm14)"	"Cryptosporidium parvum(cp17)"	0.48	0.21	1.2	0.1	0.37
"Brugia malayi (bm14)"	"Cryptosporidium parvum(cp23)"	0.93	0.51	1.78	0.83	0.96
"Brugia malayi (bm14)"	"Giardia lamblia(vsp3)"	0.81	0.47	1.38	0.44	0.76
"Brugia malayi (bm14)"	"Giardia lamblia(vsp5)"	0.95	0.51	1.74	0.87	0.96
"Brugia malayi (bm14)"	"P. falciparum(csp)"	6.36	3.25	13.32	0	0
"Brugia malayi (bm14)"	"P. falciparum(etramp5ag1)"	0.98	0.56	1.68	0.93	0.98
"Brugia malayi (bm14)"	"P. falciparum(gexp18)"	1.66	0.77	3.47	0.19	0.52
"Brugia malayi (bm14)"	"P. falciparum(glurpr2)"	1.16	0.56	2.5	0.7	0.91
"Brugia malayi (bm14)"	"P. falciparum(pfama1)"	0.62	0.29	1.39	0.23	0.58
"Brugia malayi (bm14)"	"P. falciparum(pfmsp119)"	0.87	0.49	1.53	0.62	0.87
"Brugia malayi (bm14)"	"P. falciparum(rh42)"	1.43	0.77	2.61	0.26	0.61
"Brugia malayi (bm14)"	"P. malariae(pmmsp119)"	0.92	0.49	1.65	0.78	0.95
"Brugia malayi (bm14)"	"P. ovale(pomsp119)"	0.8	0.34	1.78	0.6	0.86
"Brugia malayi (bm14)"	"P. vivax(pvdbprii)"	0.34	0.06	1.74	0.21	0.55
"Brugia malayi (bm14)"	"P. vivax(pvmsp119)"	0.47	0.16	1.25	0.14	0.45
"Brugia malayi (bm14)"	"P. vivax(pvrbp2b)"	1.07	0.23	5.02	0.93	0.98
"Brugia malayi (bm14)"	"Brugia malayi (bm33)"	1.31	0.71	2.46	0.39	0.72
"Brugia malayi (bm14)"	"Chlamydia trachomatis (ct694)"	0.57	0.3	1.06	0.08	0.33
"Brugia malayi (bm14)"	"Chlamydia trachomatis (pgp3)"	1.61	0.88	2.95	0.13	0.42
"Brugia malayi (bm14)"	"Onchocerca volvulus (ov16)"	3.26	0.98	10.04	0.04	0.25
"Brugia malayi (bm14)"	"Schistosoma mansoni (sea)"	0.76	0.39	1.53	0.43	0.76
"Brugia malayi (bm14)"	"Schistosoma mansoni (sm25)"	1.8	0.77	4.49	0.19	0.52
"Brugia malayi (bm14)"	"Strongyloides stercoralis (nie)"	1.34	0.77	2.29	0.3	0.64
"Brugia malayi (bm14)"	"Taenia solium(es33)"	0.7	0.36	1.32	0.29	0.63
"Brugia malayi (bm14)"	"Taenia solium(t24h)"	2.05	1.15	3.64	0.01	0.13
"Brugia malayi (bm14)"	"Treponema palladium (rp17)"	1.18	0.67	2.06	0.57	0.85
"Brugia malayi (bm14)"	"Treponema palladium (tmpa)"	0.61	0.22	1.53	0.31	0.66

"Brugia malayi (bm14)"	"Wuchereria bancrofti (wb123)"	1.97	1.12	3.49	0.02	0.16
"Brugia malayi (bm14)"	"Diptheria (Dip tox)"	0.66	0.37	1.21	0.17	0.49
"Brugia malayi (bm14)"	"Measles (wMev)"	1.66	0.9	3.18	0.11	0.4
"Brugia malayi (bm14)"	"Rubella (wRuv)"	0.78	0.38	1.64	0.49	0.79
"Brugia malayi (bm14)"	"Tetanus (Tet tox)"	1.3	0.81	2.11	0.28	0.62
"Brugia malayi (bm33)"	"SARS-CoV-2(sars2np)"	0.46	0.2	1.01	0.06	0.28
"Brugia malayi (bm33)"	"SARS-CoV-2(sars2rbd)"	0.74	0.21	2.49	0.63	0.88
"Brugia malayi (bm33)"	"Cryptosporidium parvum(cp17)"	1.37	0.85	2.25	0.21	0.54
"Brugia malayi (bm33)"	"Cryptosporidium parvum(cp23)"	0.75	0.52	1.07	0.11	0.4
"Brugia malayi (bm33)"	"Giardia lamblia(vsp3)"	1.39	0.95	2.02	0.09	0.35
"Brugia malayi (bm33)"	"Giardia lamblia(vsp5)"	0.79	0.51	1.22	0.29	0.64
"Brugia malayi (bm33)"	"P. falciparum(csp)"	1.36	0.95	1.95	0.09	0.35
"Brugia malayi (bm33)"	"P. falciparum(etramp5ag1)"	1.36	0.9	2.05	0.14	0.45
"Brugia malayi (bm33)"	"P. falciparum(gexp18)"	1.27	0.62	2.67	0.51	0.8
"Brugia malayi (bm33)"	"P. falciparum(glurpr2)"	0.84	0.56	1.25	0.39	0.72
"Brugia malayi (bm33)"	"P. falciparum(pfama1)"	1.1	0.73	1.66	0.64	0.89
"Brugia malayi (bm33)"	"P. falciparum(pfmsp119)"	0.84	0.59	1.18	0.32	0.66
"Brugia malayi (bm33)"	"P. falciparum(rh42)"	1.45	0.87	2.43	0.16	0.47
"Brugia malayi (bm33)"	"P. malariae(pmmsp119)"	2.06	1.24	3.47	0.01	0.07
"Brugia malayi (bm33)"	"P. ovale(pomsp119)"	2.56	1.32	5.04	0.01	0.07
"Brugia malayi (bm33)"	"P. vivax(pvdbprii)"	2.91	0.95	9.09	0.06	0.29
"Brugia malayi (bm33)"	"P. vivax(pvmsp119)"	0.87	0.29	3.02	0.81	0.96
"Brugia malayi (bm33)"	"P. vivax(pvrbp2b)"	0.36	0.11	1.09	0.07	0.31
"Brugia malayi (bm33)"	"Brugia malayi (bm14)"	1.24	0.66	2.35	0.51	0.8
"Brugia malayi (bm33)"	"Chlamydia trachomatis (ct694)"	0.89	0.57	1.38	0.59	0.86
"Brugia malayi (bm33)"	"Chlamydia trachomatis (pgp3)"	1.23	0.78	1.95	0.37	0.72
"Brugia malayi (bm33)"	"Onchocerca volvulus (ov16)"	2.54	0.54	15.15	0.27	0.62
"Brugia malayi (bm33)"	"Schistosoma mansoni (sea)"	0.99	0.69	1.43	0.96	0.99
"Brugia malayi (bm33)"	"Schistosoma mansoni (sm25)"	1.63	1.1	2.42	0.01	0.13
"Brugia malayi (bm33)"	"Strongyloides stercoralis (nie)"	1.18	0.74	1.88	0.48	0.79
"Brugia malayi (bm33)"	"Taenia solium(es33)"	1.7	1.02	2.86	0.04	0.25
"Brugia malayi (bm33)"	"Taenia solium(t24h)"	1.68	1.03	2.75	0.04	0.24
"Brugia malayi (bm33)"	"Treponema palladium (rp17)"	1.56	0.97	2.52	0.07	0.31
"Brugia malayi (bm33)"	"Treponema palladium (tmpa)"	0.86	0.34	2.26	0.76	0.94
"Brugia malayi (bm33)"	"Wuchereria bancrofti (wb123)"	1.93	1.27	2.96	0	0.04
"Brugia malayi (bm33)"	"Diptheria (Dip tox)"	1.15	0.81	1.65	0.43	0.76
"Brugia malayi (bm33)"	"Measles (wMev)"	1.38	0.97	1.95	0.07	0.31
"Brugia malayi (bm33)"	"Rubella (wRuv)"	0.73	0.48	1.1	0.13	0.44
"Brugia malayi (bm33)"	"Tetanus (Tet tox)"	0.73	0.53	0.99	0.04	0.25
"Chlamydia trachomatis (ct694)"	"SARS-CoV-2(sars2np)"	0.28	0.1	0.73	0.01	0.12
"Chlamydia trachomatis (ct694)"	"SARS-CoV-2(sars2rbd)"	0.73	0.15	3.27	0.68	0.9
"Chlamydia trachomatis (ct694)"	"Cryptosporidium parvum(cp17)"	1.22	0.67	2.25	0.53	0.82
"Chlamydia trachomatis (ct694)"	"Cryptosporidium parvum(cp23)"	1.12	0.73	1.74	0.6	0.86
"Chlamydia trachomatis (ct694)"	"Giardia lamblia(vsp3)"	0.85	0.55	1.31	0.47	0.78
"Chlamydia trachomatis (ct694)"	"Giardia lamblia(vsp5)"	0.93	0.58	1.49	0.76	0.94
"Chlamydia trachomatis (ct694)"	"P. falciparum(csp)"	1.05	0.69	1.6	0.81	0.96

"Chlamydia trachomatis (ct694)"	"P. falciparum(etramp5ag1)"	2.75	1.78	4.27	0	0
"Chlamydia trachomatis (ct694)"	"P. falciparum(gexp18)"	1.35	0.7	2.62	0.37	0.72
"Chlamydia trachomatis (ct694)"	"P. falciparum(glurpr2)"	0.87	0.54	1.4	0.57	0.85
"Chlamydia trachomatis (ct694)"	"P. falciparum(pfama1)"	1.3	0.78	2.17	0.32	0.66
"Chlamydia trachomatis (ct694)"	"P. falciparum(pfmsp119)"	1.34	0.9	2.01	0.15	0.46
"Chlamydia trachomatis (ct694)"	"P. falciparum(rh42)"	1.09	0.64	1.85	0.74	0.93
"Chlamydia trachomatis (ct694)"	"P. malariae(pmmsp119)"	2.18	1.32	3.61	0	0.04
"Chlamydia trachomatis (ct694)"	"P. ovale(pomsp119)"	1.82	0.96	3.42	0.06	0.29
"Chlamydia trachomatis (ct694)"	"P. vivax(pvdbprii)"	0.9	0.27	2.84	0.86	0.96
"Chlamydia trachomatis (ct694)"	"P. vivax(pvmsp119)"	0.72	0.29	1.83	0.49	0.79
"Chlamydia trachomatis (ct694)"	"P. vivax(pvrbp2b)"	0.44	0.14	1.43	0.16	0.49
"Chlamydia trachomatis (ct694)"	"Brugia malayi (bm14)"	0.58	0.3	1.1	0.1	0.36
"Chlamydia trachomatis (ct694)"	"Brugia malayi (bm33)"	0.74	0.47	1.16	0.2	0.53
"Chlamydia trachomatis (ct694)"	"Chlamydia trachomatis (pgp3)"	39.9	25.9	63.1	0	0
"Chlamydia trachomatis (ct694)"	"Onchocerca volvulus (ov16)"	2.22	0.51	11.14	0.31	0.65
"Chlamydia trachomatis (ct694)"	"Schistosoma mansoni (sea)"	1.11	0.7	1.75	0.65	0.89
"Chlamydia trachomatis (ct694)"	"Schistosoma mansoni (sm25)"	1.51	0.92	2.49	0.1	0.37
"Chlamydia trachomatis (ct694)"	"Strongyloides stercoralis (nie)"	0.85	0.52	1.38	0.52	0.81
"Chlamydia trachomatis (ct694)"	"Taenia solium(es33)"	1.54	0.93	2.55	0.09	0.35
"Chlamydia trachomatis (ct694)"	"Taenia solium(t24h)"	0.98	0.58	1.64	0.94	0.99
"Chlamydia trachomatis (ct694)"	"Treponema palladium (rp17)"	1.89	1.18	3.03	0.01	0.09
"Chlamydia trachomatis (ct694)"	"Treponema palladium (tmpa)"	0.91	0.33	2.43	0.84	0.96
"Chlamydia trachomatis (ct694)"	"Wuchereria bancrofti (wb123)"	1.03	0.64	1.65	0.89	0.97
"Chlamydia trachomatis (ct694)"	"Diptheria (Dip tox)"	0.96	0.63	1.48	0.85	0.96
"Chlamydia trachomatis (ct694)"	"Measles (wMev)"	1	0.67	1.5	0.99	1
"Chlamydia trachomatis (ct694)"	"Rubella (wRuv)"	0.79	0.48	1.3	0.35	0.7
"Chlamydia trachomatis (ct694)"	"Tetanus (Tet tox)"	1.08	0.75	1.55	0.68	0.9
"Chlamydia trachomatis (pgp3)"	"SARS-CoV-2(sars2np)"	1.18	0.49	2.73	0.7	0.91
"Chlamydia trachomatis (pgp3)"	"SARS-CoV-2(sars2rbd)"	2.84	0.75	10.24	0.12	0.41
"Chlamydia trachomatis (pgp3)"	"Cryptosporidium parvum(cp17)"	1.03	0.55	1.95	0.93	0.98
"Chlamydia trachomatis (pgp3)"	"Cryptosporidium parvum(cp23)"	1.76	1.13	2.75	0.01	0.13
"Chlamydia trachomatis (pgp3)"	"Giardia lamblia(vsp3)"	1.12	0.73	1.73	0.59	0.86
"Chlamydia trachomatis (pgp3)"	"Giardia lamblia(vsp5)"	0.62	0.38	1.01	0.06	0.27
"Chlamydia trachomatis (pgp3)"	"P. falciparum(csp)"	0.95	0.61	1.47	0.82	0.96
"Chlamydia trachomatis (pgp3)"	"P. falciparum(etramp5ag1)"	0.3	0.18	0.47	0	0
"Chlamydia trachomatis (pgp3)"	"P. falciparum(gexp18)"	0.8	0.38	1.69	0.56	0.85
"Chlamydia trachomatis (pgp3)"	"P. falciparum(glurpr2)"	2.13	1.3	3.54	0	0.04
"Chlamydia trachomatis (pgp3)"	"P. falciparum(pfama1)"	0.74	0.45	1.23	0.25	0.6
"Chlamydia trachomatis (pgp3)"	"P. falciparum(pfmsp119)"	0.82	0.53	1.25	0.36	0.7
"Chlamydia trachomatis (pgp3)"	"P. falciparum(rh42)"	0.97	0.57	1.64	0.92	0.98
"Chlamydia trachomatis (pgp3)"	"P. malariae(pmmsp119)"	0.6	0.35	0.99	0.05	0.26
"Chlamydia trachomatis (pgp3)"	"P. ovale(pomsp119)"	0.55	0.28	1.07	0.08	0.34
"Chlamydia trachomatis (pgp3)"	"P. vivax(pvdbprii)"	0.53	0.14	2.05	0.35	0.7
"Chlamydia trachomatis (pgp3)"	"P. vivax(pvmsp119)"	1.22	0.46	3.26	0.69	0.9
"Chlamydia trachomatis (pgp3)"	"P. vivax(pvrbp2b)"	2.23	0.59	8.2	0.23	0.58
"Chlamydia trachomatis (pgp3)"	"Brugia malayi (bm14)"	1.59	0.85	2.96	0.14	0.45

"Chlamydia trachomatis (pgp3)"	"Brugia malayi (bm33)"	1.13	0.71	1.81	0.61	0.86
"Chlamydia trachomatis (pgp3)"	"Chlamydia trachomatis (ct694)"	35.16	23.19	54.58	0	0
"Chlamydia trachomatis (pgp3)"	"Onchocerca volvulus (ov16)"	1.6	0.46	6.03	0.47	0.78
"Chlamydia trachomatis (pgp3)"	"Schistosoma mansoni (sea)"	0.73	0.46	1.14	0.17	0.49
"Chlamydia trachomatis (pgp3)"	"Schistosoma mansoni (sm25)"	1.11	0.67	1.84	0.68	0.9
"Chlamydia trachomatis (pgp3)"	"Strongyloides stercoralis (nie)"	1.32	0.83	2.11	0.24	0.59
"Chlamydia trachomatis (pgp3)"	"Taenia solium(es33)"	0.83	0.49	1.39	0.48	0.79
"Chlamydia trachomatis (pgp3)"	"Taenia solium(t24h)"	0.99	0.59	1.66	0.98	1
"Chlamydia trachomatis (pgp3)"	"Treponema palladium (rp17)"	1.19	0.74	1.92	0.47	0.78
"Chlamydia trachomatis (pgp3)"	"Treponema palladium (tmpa)"	1.48	0.62	3.52	0.37	0.72
"Chlamydia trachomatis (pgp3)"	"Wuchereria bancrofti (wb123)"	1.33	0.82	2.15	0.25	0.61
"Chlamydia trachomatis (pgp3)"	"Diptheria (Dip tox)"	0.81	0.53	1.24	0.33	0.67
"Chlamydia trachomatis (pgp3)"	"Measles (wMev)"	1.13	0.75	1.71	0.57	0.85
"Chlamydia trachomatis (pgp3)"	"Rubella (wRuv)"	2.59	1.49	4.64	0	0.02
"Chlamydia trachomatis (pgp3)"	"Tetanus (Tet tox)"	1.44	0.99	2.09	0.06	0.27
"Onchocerca volvulus (ov16)"	"SARS-CoV-2(sars2np)"	0.87	0.02	14.6	0.94	0.99
"Onchocerca volvulus (ov16)"	"SARS-CoV-2(sars2rbd)"	4.58	0.09	136.04	0.41	0.75
"Onchocerca volvulus (ov16)"	"Cryptosporidium parvum(cp17)"	0.38	0.06	3.33	0.32	0.66
"Onchocerca volvulus (ov16)"	"Cryptosporidium parvum(cp23)"	1.91	0.41	11.63	0.44	0.76
"Onchocerca volvulus (ov16)"	"Giardia lamblia(vsp3)"	0.94	0.26	3.35	0.92	0.98
"Onchocerca volvulus (ov16)"	"Giardia lamblia(vsp5)"	0.95	0.23	3.87	0.95	0.99
"Onchocerca volvulus (ov16)"	"P. falciparum(csp)"	1.05	0.29	4.23	0.94	0.99
"Onchocerca volvulus (ov16)"	"P. falciparum(etramp5ag1)"	1.8	0.51	6.38	0.36	0.7
"Onchocerca volvulus (ov16)"	"P. falciparum(gexp18)"	1.6	0.27	8.02	0.58	0.86
"Onchocerca volvulus (ov16)"	"P. falciparum(glurpr2)"	3.95	0.52	85.5	0.25	0.61
"Onchocerca volvulus (ov16)"	"P. falciparum(pfama1)"	2.88	0.38	65.08	0.38	0.72
"Onchocerca volvulus (ov16)"	"P. falciparum(pfmsp119)"	1.11	0.28	4.72	0.88	0.97
"Onchocerca volvulus (ov16)"	"P. falciparum(rh42)"	0.39	0.08	1.67	0.23	0.57
"Onchocerca volvulus (ov16)"	"P. malariae(pmmsp119)"	2.15	0.59	7.63	0.24	0.59
"Onchocerca volvulus (ov16)"	"P. ovale(pomsp119)"	2.57	0.46	12.29	0.25	0.61
"Onchocerca volvulus (ov16)"	"P. vivax(pvdbprii)"	0.84	0	126.25	0.96	0.99
"Onchocerca volvulus (ov16)"	"P. vivax(pvmsp119)"	13.98	1.58	133.02	0.02	0.16
"Onchocerca volvulus (ov16)"	"P. vivax(pvrbp2b)"	0.18	0	24.74	0.65	0.89
"Onchocerca volvulus (ov16)"	"Brugia malayi (bm14)"	4.15	1.12	15.28	0.03	0.21
"Onchocerca volvulus (ov16)"	"Brugia malayi (bm33)"	2.57	0.53	15.86	0.27	0.62
"Onchocerca volvulus (ov16)"	"Chlamydia trachomatis (ct694)"	2.04	0.49	9.34	0.34	0.68
"Onchocerca volvulus (ov16)"	"Chlamydia trachomatis (pgp3)"	2.08	0.5	9.12	0.32	0.66
"Onchocerca volvulus (ov16)"	"Schistosoma mansoni (sea)"	2.39	0.47	17.29	0.33	0.68
"Onchocerca volvulus (ov16)"	"Schistosoma mansoni (sm25)"	0.83	0.12	7.84	0.86	0.96
"Onchocerca volvulus (ov16)"	"Strongyloides stercoralis (nie)"	0.87	0.21	3.13	0.83	0.96
"Onchocerca volvulus (ov16)"	"Taenia solium(es33)"	2.74	0.72	9.9	0.13	0.42
"Onchocerca volvulus (ov16)"	"Taenia solium(t24h)"	0.29	0.05	1.41	0.15	0.46
"Onchocerca volvulus (ov16)"	"Treponema palladium (rp17)"	2.87	0.8	10.58	0.11	0.38
"Onchocerca volvulus (ov16)"	"Treponema palladium (tmpa)"	10.85	2.24	57.15	0	0.05
"Onchocerca volvulus (ov16)"	"Wuchereria bancrofti (wb123)"	1.79	0.46	7.19	0.4	0.74
"Onchocerca volvulus (ov16)"	"Diptheria (Dip tox)"	0.56	0.15	2.46	0.41	0.75

"Onchocerca volvulus (ov16)"	"Measles (wMev)"	1.67	0.41	8.2	0.5	0.79
"Onchocerca volvulus (ov16)"	"Rubella (wRuv)"	0.17	0.04	0.83	0.03	0.2
"Onchocerca volvulus (ov16)"	"Tetanus (Tet tox)"	0.28	0.07	0.91	0.05	0.25
"Schistosoma mansoni (sea)"	"SARS-CoV-2(sars2np)"	0.96	0.47	2.04	0.92	0.98
"Schistosoma mansoni (sea)"	"SARS-CoV-2(sars2rbd)"	1.04	0.36	3.21	0.94	0.99
"Schistosoma mansoni (sea)"	"Cryptosporidium parvum(cp17)"	1.39	0.94	2.05	0.1	0.37
"Schistosoma mansoni (sea)"	"Cryptosporidium parvum(cp23)"	1.05	0.75	1.46	0.78	0.95
"Schistosoma mansoni (sea)"	"Giardia lamblia(vsp3)"	1.05	0.7	1.56	0.83	0.96
"Schistosoma mansoni (sea)"	"Giardia lamblia(vsp5)"	0.57	0.37	0.87	0.01	0.1
"Schistosoma mansoni (sea)"	"P. falciparum(csp)"	1.54	1.06	2.24	0.02	0.19
"Schistosoma mansoni (sea)"	"P. falciparum(etramp5ag1)"	0.86	0.56	1.32	0.49	0.79
"Schistosoma mansoni (sea)"	"P. falciparum(gexp18)"	0.54	0.27	1.11	0.08	0.34
"Schistosoma mansoni (sea)"	"P. falciparum(glurpr2)"	1.26	0.88	1.81	0.2	0.53
"Schistosoma mansoni (sea)"	"P. falciparum(pfama1)"	1.55	1.1	2.2	0.01	0.13
"Schistosoma mansoni (sea)"	"P. falciparum(pfmsp119)"	0.78	0.56	1.09	0.14	0.45
"Schistosoma mansoni (sea)"	"P. falciparum(rh42)"	1.19	0.69	2.1	0.54	0.83
"Schistosoma mansoni (sea)"	"P. malariae(pmmsp119)"	1.84	1.04	3.45	0.04	0.25
"Schistosoma mansoni (sea)"	"P. ovale(pomsp119)"	1.17	0.61	2.34	0.64	0.89
"Schistosoma mansoni (sea)"	"P. vivax(pvdbprii)"	0.7	0.24	2.12	0.53	0.82
"Schistosoma mansoni (sea)"	"P. vivax(pvmsp119)"	1.31	0.42	5.08	0.67	0.9
"Schistosoma mansoni (sea)"	"P. vivax(pvrbp2b)"	1	0.33	2.95	0.99	1
"Schistosoma mansoni (sea)"	"Brugia malayi (bm14)"	0.7	0.37	1.37	0.29	0.63
"Schistosoma mansoni (sea)"	"Brugia malayi (bm33)"	1.06	0.74	1.51	0.77	0.94
"Schistosoma mansoni (sea)"	"Chlamydia trachomatis (ct694)"	1.03	0.66	1.63	0.89	0.97
"Schistosoma mansoni (sea)"	"Chlamydia trachomatis (pgp3)"	0.76	0.48	1.19	0.23	0.58
"Schistosoma mansoni (sea)"	"Onchocerca volvulus (ov16)"	1.08	0.27	5.69	0.92	0.98
"Schistosoma mansoni (sea)"	"Schistosoma mansoni (sm25)"	2.65	1.87	3.76	0	0
"Schistosoma mansoni (sea)"	"Strongyloides stercoralis (nie)"	0.81	0.51	1.29	0.36	0.7
"Schistosoma mansoni (sea)"	"Taenia solium(es33)"	0.93	0.55	1.62	0.8	0.96
"Schistosoma mansoni (sea)"	"Taenia solium(t24h)"	1.06	0.62	1.85	0.84	0.96
"Schistosoma mansoni (sea)"	"Treponema palladium (rp17)"	0.88	0.54	1.45	0.61	0.86
"Schistosoma mansoni (sea)"	"Treponema palladium (tmpa)"	1	0.4	2.74	0.99	1
"Schistosoma mansoni (sea)"	"Wuchereria bancrofti (wb123)"	2.18	1.33	3.67	0	0.04
"Schistosoma mansoni (sea)"	"Diptheria (Dip tox)"	0.91	0.66	1.25	0.56	0.85
"Schistosoma mansoni (sea)"	"Measles (wMev)"	1.03	0.75	1.42	0.83	0.96
"Schistosoma mansoni (sea)"	"Rubella (wRuv)"	1.34	0.93	1.92	0.12	0.41
"Schistosoma mansoni (sea)"	"Tetanus (Tet tox)"	1.03	0.77	1.39	0.82	0.96
"Schistosoma mansoni (sm25)"	"SARS-CoV-2(sars2np)"	1.83	0.76	4.52	0.18	0.51
"Schistosoma mansoni (sm25)"	"SARS-CoV-2(sars2rbd)"	1.05	0.3	3.83	0.94	0.99
"Schistosoma mansoni (sm25)"	"Cryptosporidium parvum(cp17)"	1.11	0.69	1.79	0.66	0.89
"Schistosoma mansoni (sm25)"	"Cryptosporidium parvum(cp23)"	1.64	1.13	2.4	0.01	0.1
"Schistosoma mansoni (sm25)"	"Giardia lamblia(vsp3)"	1.19	0.75	1.91	0.46	0.77
"Schistosoma mansoni (sm25)"	"Giardia lamblia(vsp5)"	1.01	0.59	1.72	0.98	1
"Schistosoma mansoni (sm25)"	"P. falciparum(csp)"	0.84	0.55	1.29	0.43	0.76
"Schistosoma mansoni (sm25)"	"P. falciparum(etramp5ag1)"	0.96	0.57	1.62	0.87	0.96
"Schistosoma mansoni (sm25)"	"P. falciparum(gexp18)"	0.97	0.35	3.01	0.96	0.99

"Schistosoma mansoni (sm25)"	"P. falciparum(glurpr2)"	1.81	1.21	2.72	0	0.06
"Schistosoma mansoni (sm25)"	"P. falciparum(pfama1)"	1.33	0.88	2.02	0.18	0.5
"Schistosoma mansoni (sm25)"	"P. falciparum(pfmsp119)"	1.31	0.9	1.91	0.16	0.47
"Schistosoma mansoni (sm25)"	"P. falciparum(rh42)"	1.87	0.91	4.04	0.1	0.36
"Schistosoma mansoni (sm25)"	"P. malariae(pmmsp119)"	1.08	0.57	2.14	0.81	0.96
"Schistosoma mansoni (sm25)"	"P. ovale(pomsp119)"	1.65	0.71	3.94	0.25	0.61
"Schistosoma mansoni (sm25)"	"P. vivax(pvdbprii)"	0.16	0.04	0.59	0.01	0.07
"Schistosoma mansoni (sm25)"	"P. vivax(pvmsp119)"	1.37	0.15	39.93	0.82	0.96
"Schistosoma mansoni (sm25)"	"P. vivax(pvrbp2b)"	0.59	0.16	2.1	0.41	0.74
"Schistosoma mansoni (sm25)"	"Brugia malayi (bm14)"	1.17	0.49	3.09	0.73	0.93
"Schistosoma mansoni (sm25)"	"Brugia malayi (bm33)"	1.83	1.22	2.77	0	0.05
"Schistosoma mansoni (sm25)"	"Chlamydia trachomatis (ct694)"	1.68	0.98	2.91	0.06	0.29
"Schistosoma mansoni (sm25)"	"Chlamydia trachomatis (pgp3)"	0.99	0.56	1.72	0.96	0.99
"Schistosoma mansoni (sm25)"	"Onchocerca volvulus (ov16)"	1.67	0.16	21.65	0.68	0.9
"Schistosoma mansoni (sm25)"	"Schistosoma mansoni (sea)"	2.6	1.81	3.76	0	0
"Schistosoma mansoni (sm25)"	"Strongyloides stercoralis (nie)"	1.48	0.81	2.76	0.21	0.55
"Schistosoma mansoni (sm25)"	"Taenia solium(es33)"	1.52	0.73	3.36	0.28	0.62
"Schistosoma mansoni (sm25)"	"Taenia solium(t24h)"	5.37	2.14	16.22	0	0.02
"Schistosoma mansoni (sm25)"	"Treponema palladium (rp17)"	1.3	0.7	2.46	0.41	0.75
"Schistosoma mansoni (sm25)"	"Treponema palladium (tmpa)"	1.37	0.33	6.47	0.68	0.9
"Schistosoma mansoni (sm25)"	"Wuchereria bancrofti (wb123)"	1.62	0.89	3.07	0.12	0.42
"Schistosoma mansoni (sm25)"	"Diptheria (Dip tox)"	1.11	0.76	1.61	0.59	0.86
"Schistosoma mansoni (sm25)"	"Measles (wMev)"	1.12	0.77	1.63	0.54	0.83
"Schistosoma mansoni (sm25)"	"Rubella (wRuv)"	1.16	0.75	1.79	0.51	0.81
"Schistosoma mansoni (sm25)"	"Tetanus (Tet tox)"	0.99	0.69	1.41	0.93	0.99
"Strongyloides stercoralis (nie)"	"SARS-CoV-2(sars2np)"	1.4	0.62	3.03	0.4	0.74
"Strongyloides stercoralis (nie)"	"SARS-CoV-2(sars2rbd)"	0.68	0.16	2.42	0.57	0.85
"Strongyloides stercoralis (nie)"	"Cryptosporidium parvum(cp17)"	2.17	1.03	5.14	0.06	0.27
"Strongyloides stercoralis (nie)"	"Cryptosporidium parvum(cp23)"	0.86	0.55	1.35	0.5	0.79
"Strongyloides stercoralis (nie)"	"Giardia lamblia(vsp3)"	0.96	0.63	1.44	0.83	0.96
"Strongyloides stercoralis (nie)"	"Giardia lamblia(vsp5)"	0.89	0.55	1.4	0.61	0.86
"Strongyloides stercoralis (nie)"	"P. falciparum(csp)"	1.23	0.81	1.87	0.34	0.68
"Strongyloides stercoralis (nie)"	"P. falciparum(etramp5ag1)"	1.16	0.76	1.74	0.49	0.79
"Strongyloides stercoralis (nie)"	"P. falciparum(gexp18)"	1.82	1	3.27	0.05	0.25
"Strongyloides stercoralis (nie)"	"P. falciparum(glurpr2)"	0.83	0.5	1.39	0.48	0.79
"Strongyloides stercoralis (nie)"	"P. falciparum(pfama1)"	0.8	0.46	1.41	0.43	0.76
"Strongyloides stercoralis (nie)"	"P. falciparum(pfmsp119)"	1.53	1	2.35	0.05	0.26
"Strongyloides stercoralis (nie)"	"P. falciparum(rh42)"	1.3	0.81	2.06	0.28	0.62
"Strongyloides stercoralis (nie)"	"P. malariae(pmmsp119)"	1.13	0.71	1.79	0.6	0.86
"Strongyloides stercoralis (nie)"	"P. ovale(pomsp119)"	0.84	0.45	1.55	0.6	0.86
"Strongyloides stercoralis (nie)"	"P. vivax(pvdbprii)"	0.38	0.09	1.35	0.15	0.46
"Strongyloides stercoralis (nie)"	"P. vivax(pvmsp119)"	1.07	0.48	2.35	0.87	0.96
"Strongyloides stercoralis (nie)"	"P. vivax(pvrbp2b)"	3.01	0.87	11.48	0.09	0.35
"Strongyloides stercoralis (nie)"	"Brugia malayi (bm14)"	1.05	0.6	1.81	0.86	0.96
"Strongyloides stercoralis (nie)"	"Brugia malayi (bm33)"	1.02	0.64	1.6	0.94	0.99
"Strongyloides stercoralis (nie)"	"Chlamydia trachomatis (ct694)"	0.91	0.57	1.45	0.7	0.91

"Strongyloides stercoralis (nie)"	"Chlamydia trachomatis (pgp3)"	1.3	0.82	2.05	0.27	0.62
"Strongyloides stercoralis (nie)"	"Onchocerca volvulus (ov16)"	0.67	0.19	2.1	0.51	0.8
"Strongyloides stercoralis (nie)"	"Schistosoma mansoni (sea)"	0.8	0.5	1.29	0.35	0.7
"Strongyloides stercoralis (nie)"	"Schistosoma mansoni (sm25)"	1.76	1.01	3.16	0.05	0.27
"Strongyloides stercoralis (nie)"	"Taenia solium(es33)"	1.05	0.65	1.68	0.83	0.96
"Strongyloides stercoralis (nie)"	"Taenia solium(t24h)"	0.84	0.52	1.34	0.47	0.78
"Strongyloides stercoralis (nie)"	"Treponema palladium (rp17)"	1.29	0.83	1.98	0.26	0.61
"Strongyloides stercoralis (nie)"	"Treponema palladium (tmpa)"	1.11	0.5	2.39	0.79	0.95
"Strongyloides stercoralis (nie)"	"Wuchereria bancrofti (wb123)"	1.23	0.79	1.9	0.37	0.71
"Strongyloides stercoralis (nie)"	"Diptheria (Dip tox)"	1.11	0.71	1.75	0.66	0.89
"Strongyloides stercoralis (nie)"	"Measles (wMev)"	1.27	0.83	1.98	0.27	0.62
"Strongyloides stercoralis (nie)"	"Rubella (wRuv)"	0.9	0.53	1.57	0.71	0.92
"Strongyloides stercoralis (nie)"	"Tetanus (Tet tox)"	0.99	0.69	1.43	0.98	1
"Taenia solium(es33)"	"SARS-CoV-2(sars2np)"	1.24	0.44	3.05	0.66	0.89
"Taenia solium(es33)"	"SARS-CoV-2(sars2rbd)"	1.52	0.35	5.86	0.56	0.85
"Taenia solium(es33)"	"Cryptosporidium parvum(cp17)"	1.12	0.51	2.73	0.8	0.95
"Taenia solium(es33)"	"Cryptosporidium parvum(cp23)"	1.3	0.79	2.19	0.31	0.65
"Taenia solium(es33)"	"Giardia lamblia(vsp3)"	0.66	0.42	1.03	0.07	0.31
"Taenia solium(es33)"	"Giardia lamblia(vsp5)"	1.59	0.99	2.54	0.05	0.27
"Taenia solium(es33)"	"P. falciparum(csp)"	1.2	0.77	1.89	0.42	0.75
"Taenia solium(es33)"	"P. falciparum(etramp5ag1)"	1.03	0.65	1.59	0.91	0.98
"Taenia solium(es33)"	"P. falciparum(gexp18)"	1.28	0.67	2.36	0.45	0.77
"Taenia solium(es33)"	"P. falciparum(glurpr2)"	0.75	0.45	1.28	0.29	0.64
"Taenia solium(es33)"	"P. falciparum(pfama1)"	1.98	1.06	3.92	0.04	0.24
"Taenia solium(es33)"	"P. falciparum(pfmsp119)"	0.91	0.58	1.43	0.68	0.9
"Taenia solium(es33)"	"P. falciparum(rh42)"	0.81	0.48	1.36	0.44	0.76
"Taenia solium(es33)"	"P. malariae(pmmsp119)"	0.98	0.59	1.6	0.93	0.98
"Taenia solium(es33)"	"P. ovale(pomsp119)"	1.52	0.83	2.72	0.16	0.48
"Taenia solium(es33)"	"P. vivax(pvdbprii)"	1.06	0.33	3.2	0.92	0.98
"Taenia solium(es33)"	"P. vivax(pvmsp119)"	0.81	0.35	1.81	0.62	0.87
"Taenia solium(es33)"	"P. vivax(pvrbp2b)"	1	0.33	3.16	1	1
"Taenia solium(es33)"	"Brugia malayi (bm14)"	0.65	0.34	1.19	0.18	0.5
"Taenia solium(es33)"	"Brugia malayi (bm33)"	1.98	1.21	3.28	0.01	0.08
"Taenia solium(es33)"	"Chlamydia trachomatis (ct694)"	1.27	0.78	2.09	0.34	0.68
"Taenia solium(es33)"	"Chlamydia trachomatis (pgp3)"	0.86	0.52	1.42	0.55	0.84
"Taenia solium(es33)"	"Onchocerca volvulus (ov16)"	2.19	0.73	6.35	0.15	0.46
"Taenia solium(es33)"	"Schistosoma mansoni (sea)"	1.08	0.63	1.88	0.79	0.95
"Taenia solium(es33)"	"Schistosoma mansoni (sm25)"	2.02	1.05	4.05	0.04	0.24
"Taenia solium(es33)"	"Strongyloides stercoralis (nie)"	1.07	0.66	1.7	0.78	0.95
"Taenia solium(es33)"	"Taenia solium(t24h)"	1.62	1.01	2.56	0.04	0.25
"Taenia solium(es33)"	"Treponema palladium (rp17)"	1.49	0.93	2.37	0.09	0.35
"Taenia solium(es33)"	"Treponema palladium (tmpa)"	1.2	0.52	2.61	0.66	0.89
"Taenia solium(es33)"	"Wuchereria bancrofti (wb123)"	1.29	0.82	2.03	0.27	0.62
"Taenia solium(es33)"	"Diptheria (Dip tox)"	2.13	1.24	3.85	0.01	0.1
"Taenia solium(es33)"	"Measles (wMev)"	1.21	0.76	1.97	0.42	0.75
"Taenia solium(es33)"	"Rubella (wRuv)"	0.97	0.55	1.73	0.91	0.98

"Taenia solium(es33)"	"Tetanus (Tet tox)"	1.07	0.73	1.58	0.73	0.93
"Taenia solium(t24h)"	"SARS-CoV-2(sars2np)"	0.95	0.38	2.21	0.9	0.98
"Taenia solium(t24h)"	"SARS-CoV-2(sars2rbd)"	0.47	0.08	2.17	0.38	0.72
"Taenia solium(t24h)"	"Cryptosporidium parvum(cp17)"	5.43	1.52	35.09	0.03	0.2
"Taenia solium(t24h)"	"Cryptosporidium parvum(cp23)"	1.69	0.99	2.98	0.06	0.28
"Taenia solium(t24h)"	"Giardia lamblia(vsp3)"	1.17	0.74	1.85	0.5	0.8
"Taenia solium(t24h)"	"Giardia lamblia(vsp5)"	1.38	0.84	2.26	0.2	0.53
"Taenia solium(t24h)"	"P. falciparum(csp)"	1.07	0.68	1.7	0.76	0.94
"Taenia solium(t24h)"	"P. falciparum(etramp5ag1)"	1.49	0.95	2.31	0.08	0.33
"Taenia solium(t24h)"	"P. falciparum(gexp18)"	1.15	0.6	2.18	0.67	0.9
"Taenia solium(t24h)"	"P. falciparum(glurpr2)"	1.05	0.61	1.84	0.87	0.96
"Taenia solium(t24h)"	"P. falciparum(pfama1)"	1.08	0.57	2.13	0.81	0.96
"Taenia solium(t24h)"	"P. falciparum(pfmsp119)"	0.63	0.4	1.01	0.05	0.27
"Taenia solium(t24h)"	"P. falciparum(rh42)"	1.45	0.88	2.37	0.14	0.45
"Taenia solium(t24h)"	"P. malariae(pmmsp119)"	0.8	0.47	1.35	0.41	0.75
"Taenia solium(t24h)"	"P. ovale(pomsp119)"	0.84	0.43	1.6	0.6	0.86
"Taenia solium(t24h)"	"P. vivax(pvdbprii)"	0.28	0.08	0.93	0.04	0.25
"Taenia solium(t24h)"	"P. vivax(pvmsp119)"	4.12	1.74	10.44	0	0.03
"Taenia solium(t24h)"	"P. vivax(pvrbp2b)"	0.75	0.22	2.74	0.65	0.89
"Taenia solium(t24h)"	"Brugia malayi (bm14)"	1.93	1.06	3.48	0.03	0.21
"Taenia solium(t24h)"	"Brugia malayi (bm33)"	1.44	0.88	2.36	0.15	0.46
"Taenia solium(t24h)"	"Chlamydia trachomatis (ct694)"	0.97	0.59	1.6	0.91	0.98
"Taenia solium(t24h)"	"Chlamydia trachomatis (pgp3)"	1.04	0.63	1.72	0.88	0.96
"Taenia solium(t24h)"	"Onchocerca volvulus (ov16)"	0.33	0.08	1.32	0.13	0.42
"Taenia solium(t24h)"	"Schistosoma mansoni (sea)"	1.09	0.62	1.98	0.76	0.94
"Taenia solium(t24h)"	"Schistosoma mansoni (sm25)"	6.12	2.57	17.38	0	0
"Taenia solium(t24h)"	"Strongyloides stercoralis (nie)"	0.82	0.5	1.32	0.42	0.75
"Taenia solium(t24h)"	"Taenia solium(es33)"	1.56	0.96	2.53	0.07	0.31
"Taenia solium(t24h)"	"Treponema palladium (rp17)"	0.63	0.37	1.05	0.08	0.34
"Taenia solium(t24h)"	"Treponema palladium (tmpa)"	2.07	0.88	4.79	0.09	0.35
"Taenia solium(t24h)"	"Wuchereria bancrofti (wb123)"	2.44	1.57	3.8	0	0
"Taenia solium(t24h)"	"Diptheria (Dip tox)"	0.82	0.49	1.38	0.44	0.76
"Taenia solium(t24h)"	"Measles (wMev)"	1.01	0.63	1.64	0.96	0.99
"Taenia solium(t24h)"	"Rubella (wRuv)"	1.75	0.95	3.35	0.08	0.34
"Taenia solium(t24h)"	"Tetanus (Tet tox)"	0.97	0.65	1.45	0.89	0.97
"Treponema palladium (rp17)"	"SARS-CoV-2(sars2np)"	1.08	0.4	2.67	0.87	0.96
"Treponema palladium (rp17)"	"SARS-CoV-2(sars2rbd)"	0.29	0.03	1.49	0.18	0.51
"Treponema palladium (rp17)"	"Cryptosporidium parvum(cp17)"	1.1	0.56	2.29	0.8	0.96
"Treponema palladium (rp17)"	"Cryptosporidium parvum(cp23)"	0.87	0.55	1.38	0.54	0.83
"Treponema palladium (rp17)"	"Giardia lamblia(vsp3)"	1.56	1.03	2.39	0.04	0.24
"Treponema palladium (rp17)"	"Giardia lamblia(vsp5)"	1.44	0.92	2.27	0.11	0.4
"Treponema palladium (rp17)"	"P. falciparum(csp)"	1.23	0.81	1.88	0.33	0.68
"Treponema palladium (rp17)"	"P. falciparum(etramp5ag1)"	1.36	0.9	2.06	0.14	0.45
"Treponema palladium (rp17)"	"P. falciparum(gexp18)"	0.76	0.4	1.41	0.39	0.73
"Treponema palladium (rp17)"	"P. falciparum(glurpr2)"	1.17	0.69	2.03	0.56	0.85
"Treponema palladium (rp17)"	"P. falciparum(pfama1)"	1.17	0.65	2.18	0.61	0.86

"Treponema palladium (rp17)"	"P. falciparum(pfmsp119)"	1.99	1.28	3.12	0	0.04
"Treponema palladium (rp17)"	"P. falciparum(rh42)"	1.14	0.7	1.83	0.61	0.86
"Treponema palladium (rp17)"	"P. malariae(pmmsp119)"	1.32	0.83	2.09	0.24	0.59
"Treponema palladium (rp17)"	"P. ovale(pomsp119)"	0.62	0.32	1.17	0.15	0.46
"Treponema palladium (rp17)"	"P. vivax(pvdbprii)"	0.46	0.12	1.63	0.24	0.59
"Treponema palladium (rp17)"	"P. vivax(pvmsp119)"	1.01	0.44	2.28	0.98	1
"Treponema palladium (rp17)"	"P. vivax(pvrbp2b)"	1.94	0.56	7.12	0.31	0.65
"Treponema palladium (rp17)"	"Brugia malayi (bm14)"	1.19	0.68	2.07	0.53	0.82
"Treponema palladium (rp17)"	"Brugia malayi (bm33)"	1.58	1	2.53	0.05	0.27
"Treponema palladium (rp17)"	"Chlamydia trachomatis (ct694)"	1.85	1.16	2.94	0.01	0.1
"Treponema palladium (rp17)"	"Chlamydia trachomatis (pgp3)"	1.11	0.69	1.78	0.65	0.89
"Treponema palladium (rp17)"	"Onchocerca volvulus (ov16)"	2.32	0.75	7.25	0.14	0.45
"Treponema palladium (rp17)"	"Schistosoma mansoni (sea)"	0.97	0.59	1.61	0.91	0.98
"Treponema palladium (rp17)"	"Schistosoma mansoni (sm25)"	1.38	0.77	2.54	0.29	0.63
"Treponema palladium (rp17)"	"Strongyloides stercoralis (nie)"	1.38	0.89	2.13	0.15	0.46
"Treponema palladium (rp17)"	"Taenia solium(es33)"	1.43	0.88	2.28	0.14	0.45
"Treponema palladium (rp17)"	"Taenia solium(t24h)"	0.59	0.35	0.96	0.04	0.24
"Treponema palladium (rp17)"	"Treponema palladium (tmpa)"	7.59	3.55	16.86	0	0
"Treponema palladium (rp17)"	"Wuchereria bancrofti (wb123)"	1.22	0.78	1.88	0.38	0.72
"Treponema palladium (rp17)"	"Diptheria (Dip tox)"	0.87	0.56	1.39	0.56	0.85
"Treponema palladium (rp17)"	"Measles (wMev)"	1.53	0.98	2.44	0.07	0.29
"Treponema palladium (rp17)"	"Rubella (wRuv)"	0.66	0.39	1.13	0.12	0.42
"Treponema palladium (rp17)"	"Tetanus (Tet tox)"	0.87	0.6	1.25	0.45	0.77
"Treponema palladium (tmpa)"	"SARS-CoV-2(sars2np)"	0.68	0.03	4.25	0.73	0.93
"Treponema palladium (tmpa)"	"SARS-CoV-2(sars2rbd)"	0	0	529916564.05	0.99	1
"Treponema palladium (tmpa)"	"Cryptosporidium parvum(cp17)"	0.31	0.1	1.03	0.05	0.26
"Treponema palladium (tmpa)"	"Cryptosporidium parvum(cp23)"	0.89	0.37	2.31	0.81	0.96
"Treponema palladium (tmpa)"	"Giardia lamblia(vsp3)"	1.17	0.51	2.72	0.71	0.92
"Treponema palladium (tmpa)"	"Giardia lamblia(vsp5)"	1.42	0.62	3.2	0.4	0.74
"Treponema palladium (tmpa)"	"P. falciparum(csp)"	1.76	0.76	4.34	0.2	0.53
"Treponema palladium (tmpa)"	"P. falciparum(etramp5ag1)"	0.5	0.21	1.14	0.11	0.39
"Treponema palladium (tmpa)"	"P. falciparum(gexp18)"	1.39	0.4	4.19	0.58	0.86
"Treponema palladium (tmpa)"	"P. falciparum(glurpr2)"	1.73	0.55	6.34	0.38	0.72
"Treponema palladium (tmpa)"	"P. falciparum(pfama1)"	3.7	0.9	25.68	0.11	0.39
"Treponema palladium (tmpa)"	"P. falciparum(pfmsp119)"	1.23	0.52	3.07	0.64	0.89
"Treponema palladium (tmpa)"	"P. falciparum(rh42)"	2.12	0.88	5.08	0.09	0.35
"Treponema palladium (tmpa)"	"P. malariae(pmmsp119)"	0.66	0.25	1.62	0.38	0.72
"Treponema palladium (tmpa)"	"P. ovale(pomsp119)"	1.22	0.32	4	0.76	0.94
"Treponema palladium (tmpa)"	"P. vivax(pvdbprii)"	0.73	0.03	8.28	0.82	0.96
"Treponema palladium (tmpa)"	"P. vivax(pvmsp119)"	0.16	0.02	0.9	0.05	0.27
"Treponema palladium (tmpa)"	"P. vivax(pvrbp2b)"	0.16	0.01	2.2	0.21	0.56
"Treponema palladium (tmpa)"	"Brugia malayi (bm14)"	0.68	0.23	1.82	0.46	0.78
"Treponema palladium (tmpa)"	"Brugia malayi (bm33)"	0.9	0.34	2.41	0.82	0.96
"Treponema palladium (tmpa)"	"Chlamydia trachomatis (ct694)"	0.59	0.23	1.51	0.28	0.62
"Treponema palladium (tmpa)"	"Chlamydia trachomatis (pgp3)"	1.83	0.77	4.42	0.18	0.5
"Treponema palladium (tmpa)"	"Onchocerca volvulus (ov16)"	8.99	2.23	35.04	0	0.03

"Treponema palladium (tmpa)"	"Schistosoma mansoni (sea)"	0.67	0.25	1.9	0.43	0.76
"Treponema palladium (tmpa)"	"Schistosoma mansoni (sm25)"	1	0.29	3.78	0.99	1
"Treponema palladium (tmpa)"	"Strongyloides stercoralis (nie)"	1.09	0.45	2.52	0.84	0.96
"Treponema palladium (tmpa)"	"Taenia solium(es33)"	1.44	0.59	3.37	0.41	0.74
"Treponema palladium (tmpa)"	"Taenia solium(t24h)"	2.67	1.11	6.39	0.03	0.2
"Treponema palladium (tmpa)"	"Treponema palladium (rp17)"	8.42	3.95	18.75	0	0
"Treponema palladium (tmpa)"	"Wuchereria bancrofti (wb123)"	0.87	0.37	2.04	0.75	0.93
"Treponema palladium (tmpa)"	"Diptheria (Dip tox)"	1.55	0.62	4.39	0.37	0.72
"Treponema palladium (tmpa)"	"Measles (wMev)"	0.99	0.41	2.53	0.98	1
"Treponema palladium (tmpa)"	"Rubella (wRuv)"	3.06	0.86	15.27	0.12	0.41
"Treponema palladium (tmpa)"	"Tetanus (Tet tox)"	0.93	0.47	1.85	0.84	0.96
"Wuchereria bancrofti (wb123)"	"SARS-CoV-2(sars2np)"	1.04	0.42	2.44	0.93	0.98
"Wuchereria bancrofti (wb123)"	"SARS-CoV-2(sars2rbd)"	0.55	0.1	2.26	0.44	0.76
"Wuchereria bancrofti (wb123)"	"Cryptosporidium parvum(cp17)"	1.66	0.8	3.79	0.2	0.53
"Wuchereria bancrofti (wb123)"	"Cryptosporidium parvum(cp23)"	0.72	0.47	1.11	0.13	0.44
"Wuchereria bancrofti (wb123)"	"Giardia lamblia(vsp3)"	0.97	0.64	1.45	0.87	0.96
"Wuchereria bancrofti (wb123)"	"Giardia lamblia(vsp5)"	1.46	0.93	2.29	0.1	0.36
"Wuchereria bancrofti (wb123)"	"P. falciparum(csp)"	1.94	1.3	2.89	0	0.02
"Wuchereria bancrofti (wb123)"	"P. falciparum(etramp5ag1)"	1.23	0.81	1.86	0.32	0.66
"Wuchereria bancrofti (wb123)"	"P. falciparum(gexp18)"	1.52	0.81	2.85	0.19	0.52
"Wuchereria bancrofti (wb123)"	"P. falciparum(glurpr2)"	0.88	0.54	1.43	0.6	0.86
"Wuchereria bancrofti (wb123)"	"P. falciparum(pfama1)"	0.91	0.53	1.57	0.72	0.92
"Wuchereria bancrofti (wb123)"	"P. falciparum(pfmsp119)"	1.09	0.72	1.66	0.67	0.9
"Wuchereria bancrofti (wb123)"	"P. falciparum(rh42)"	0.9	0.55	1.46	0.68	0.9
"Wuchereria bancrofti (wb123)"	"P. malariae(pmmsp119)"	0.99	0.62	1.58	0.96	0.99
"Wuchereria bancrofti (wb123)"	"P. ovale(pomsp119)"	1.09	0.59	1.97	0.79	0.95
"Wuchereria bancrofti (wb123)"	"P. vivax(pvdbprii)"	0.73	0.21	2.29	0.6	0.86
"Wuchereria bancrofti (wb123)"	"P. vivax(pvmsp119)"	1.43	0.59	3.63	0.44	0.76
"Wuchereria bancrofti (wb123)"	"P. vivax(pvrbp2b)"	0.72	0.23	2.49	0.59	0.86
"Wuchereria bancrofti (wb123)"	"Brugia malayi (bm14)"	2.15	1.23	3.79	0.01	0.09
"Wuchereria bancrofti (wb123)"	"Brugia malayi (bm33)"	2.31	1.52	3.51	0	0
"Wuchereria bancrofti (wb123)"	"Chlamydia trachomatis (ct694)"	0.96	0.61	1.52	0.87	0.96
"Wuchereria bancrofti (wb123)"	"Chlamydia trachomatis (pgp3)"	1.37	0.85	2.21	0.2	0.53
"Wuchereria bancrofti (wb123)"	"Onchocerca volvulus (ov16)"	1.83	0.54	6.88	0.35	0.69
"Wuchereria bancrofti (wb123)"	"Schistosoma mansoni (sea)"	2.5	1.49	4.3	0	0.02
"Wuchereria bancrofti (wb123)"	"Schistosoma mansoni (sm25)"	1.79	1.01	3.27	0.05	0.27
"Wuchereria bancrofti (wb123)"	"Strongyloides stercoralis (nie)"	1.23	0.78	1.92	0.38	0.72
"Wuchereria bancrofti (wb123)"	"Taenia solium(es33)"	1.3	0.82	2.04	0.26	0.61
"Wuchereria bancrofti (wb123)"	"Taenia solium(t24h)"	2.4	1.56	3.7	0	0
"Wuchereria bancrofti (wb123)"	"Treponema palladium (rp17)"	1.21	0.77	1.87	0.4	0.74
"Wuchereria bancrofti (wb123)"	"Treponema palladium (tmpa)"	0.89	0.39	2.05	0.79	0.95
"Wuchereria bancrofti (wb123)"	"Diptheria (Dip tox)"	1.38	0.88	2.19	0.17	0.49
"Wuchereria bancrofti (wb123)"	"Measles (wMev)"	1.49	0.98	2.29	0.06	0.29
"Wuchereria bancrofti (wb123)"	"Rubella (wRuv)"	0.67	0.4	1.12	0.12	0.42
"Wuchereria bancrofti (wb123)"	"Tetanus (Tet tox)"	0.73	0.51	1.05	0.09	0.35
"Diptheria (Dip tox)"	"SARS-CoV-2(sars2np)"	0.38	0.2	0.72	0	0.04

"Diptheria (Dip tox)"	"SARS-CoV-2(sars2rbd)"	1.34	0.49	3.76	0.57	0.85
"Diptheria (Dip tox)"	"Cryptosporidium parvum(cp17)"	0.98	0.66	1.43	0.91	0.98
"Diptheria (Dip tox)"	"Cryptosporidium parvum(cp23)"	1.63	1.19	2.22	0	0.04
"Diptheria (Dip tox)"	"Giardia lamblia(vsp3)"	0.81	0.56	1.17	0.26	0.61
"Diptheria (Dip tox)"	"Giardia lamblia(vsp5)"	1.32	0.88	1.99	0.19	0.52
"Diptheria (Dip tox)"	"P. falciparum(csp)"	1.41	1	2.01	0.05	0.27
"Diptheria (Dip tox)"	"P. falciparum(etramp5ag1)"	1.27	0.84	1.92	0.26	0.61
"Diptheria (Dip tox)"	"P. falciparum(gexp18)"	0.75	0.39	1.46	0.38	0.72
"Diptheria (Dip tox)"	"P. falciparum(glurpr2)"	1.52	1.07	2.15	0.02	0.16
"Diptheria (Dip tox)"	"P. falciparum(pfama1)"	0.74	0.52	1.05	0.09	0.36
"Diptheria (Dip tox)"	"P. falciparum(pfmsp119)"	1.05	0.77	1.44	0.74	0.93
"Diptheria (Dip tox)"	"P. falciparum(rh42)"	0.61	0.38	0.98	0.04	0.24
"Diptheria (Dip tox)"	"P. malariae(pmmsp119)"	1.48	0.9	2.51	0.13	0.44
"Diptheria (Dip tox)"	"P. ovale(pomsp119)"	0.65	0.37	1.17	0.14	0.45
"Diptheria (Dip tox)"	"P. vivax(pvdbprii)"	0.9	0.3	2.84	0.86	0.96
"Diptheria (Dip tox)"	"P. vivax(pvmsp119)"	1.91	0.64	7.22	0.28	0.63
"Diptheria (Dip tox)"	"P. vivax(pvrbp2b)"	0.73	0.24	2.14	0.56	0.85
"Diptheria (Dip tox)"	"Brugia malayi (bm14)"	0.75	0.42	1.36	0.32	0.66
"Diptheria (Dip tox)"	"Brugia malayi (bm33)"	1.2	0.85	1.71	0.3	0.64
"Diptheria (Dip tox)"	"Chlamydia trachomatis (ct694)"	1	0.65	1.53	1	1
"Diptheria (Dip tox)"	"Chlamydia trachomatis (pgp3)"	0.83	0.54	1.27	0.39	0.73
"Diptheria (Dip tox)"	"Onchocerca volvulus (ov16)"	0.79	0.24	3.23	0.72	0.92
"Diptheria (Dip tox)"	"Schistosoma mansoni (sea)"	0.89	0.65	1.22	0.47	0.78
"Diptheria (Dip tox)"	"Schistosoma mansoni (sm25)"	1.14	0.8	1.62	0.46	0.78
"Diptheria (Dip tox)"	"Strongyloides stercoralis (nie)"	1.12	0.72	1.78	0.61	0.86
"Diptheria (Dip tox)"	"Taenia solium(es33)"	2.03	1.18	3.66	0.01	0.13
"Diptheria (Dip tox)"	"Taenia solium(t24h)"	0.94	0.58	1.56	0.82	0.96
"Diptheria (Dip tox)"	"Treponema palladium (rp17)"	0.9	0.58	1.43	0.66	0.89
"Diptheria (Dip tox)"	"Treponema palladium (tmpa)"	1.62	0.68	4.36	0.3	0.65
"Diptheria (Dip tox)"	"Wuchereria bancrofti (wb123)"	1.37	0.89	2.14	0.16	0.47
"Diptheria (Dip tox)"	"Measles (wMev)"	1.36	1.01	1.83	0.04	0.25
"Diptheria (Dip tox)"	"Rubella (wRuv)"	1.4	0.99	1.98	0.05	0.27
"Diptheria (Dip tox)"	"Tetanus (Tet tox)"	1.78	1.34	2.35	0	0
"Measles (wMev)"	"SARS-CoV-2(sars2np)"	1.92	0.9	4.55	0.11	0.4
"Measles (wMev)"	"SARS-CoV-2(sars2rbd)"	0.46	0.15	1.4	0.17	0.49
"Measles (wMev)"	"Cryptosporidium parvum(cp17)"	1.48	1.01	2.18	0.05	0.25
"Measles (wMev)"	"Cryptosporidium parvum(cp23)"	1.3	0.95	1.77	0.1	0.37
"Measles (wMev)"	"Giardia lamblia(vsp3)"	0.94	0.66	1.32	0.7	0.91
"Measles (wMev)"	"Giardia lamblia(vsp5)"	1.56	1.06	2.31	0.03	0.2
"Measles (wMev)"	"P. falciparum(csp)"	1.07	0.77	1.49	0.69	0.9
"Measles (wMev)"	"P. falciparum(etramp5ag1)"	0.68	0.47	0.99	0.04	0.25
"Measles (wMev)"	"P. falciparum(gexp18)"	1.11	0.6	2.13	0.74	0.93
"Measles (wMev)"	"P. falciparum(glurpr2)"	0.95	0.67	1.34	0.76	0.94
"Measles (wMev)"	"P. falciparum(pfama1)"	0.73	0.51	1.04	0.08	0.34
"Measles (wMev)"	"P. falciparum(pfmsp119)"	1.16	0.85	1.57	0.34	0.69
"Measles (wMev)"	"P. falciparum(rh42)"	1.01	0.64	1.6	0.98	1

"Measles (wMev)"	"P. malariae(pmmsp119)"	1.21	0.77	1.93	0.41	0.74
"Measles (wMev)"	"P. ovale(pomsp119)"	2.11	1.13	4.18	0.02	0.19
"Measles (wMev)"	"P. vivax(pvdbprii)"	1.05	0.37	3.14	0.93	0.98
"Measles (wMev)"	"P. vivax(pvmsp119)"	1.11	0.46	2.86	0.83	0.96
"Measles (wMev)"	"P. vivax(pvrbp2b)"	0.47	0.16	1.3	0.15	0.46
"Measles (wMev)"	"Brugia malayi (bm14)"	1.63	0.92	3.01	0.1	0.38
"Measles (wMev)"	"Brugia malayi (bm33)"	1.43	1.02	1.99	0.04	0.24
"Measles (wMev)"	"Chlamydia trachomatis (ct694)"	0.95	0.64	1.41	0.8	0.95
"Measles (wMev)"	"Chlamydia trachomatis (pgp3)"	1.17	0.78	1.77	0.45	0.77
"Measles (wMev)"	"Onchocerca volvulus (ov16)"	1.25	0.37	5.37	0.74	0.93
"Measles (wMev)"	"Schistosoma mansoni (sea)"	1.06	0.77	1.45	0.72	0.92
"Measles (wMev)"	"Schistosoma mansoni (sm25)"	1.13	0.79	1.61	0.5	0.79
"Measles (wMev)"	"Strongyloides stercoralis (nie)"	1.23	0.81	1.89	0.34	0.68
"Measles (wMev)"	"Taenia solium(es33)"	1.28	0.81	2.05	0.3	0.65
"Measles (wMev)"	"Taenia solium(t24h)"	0.86	0.55	1.36	0.51	0.8
"Measles (wMev)"	"Treponema palladium (rp17)"	1.36	0.89	2.11	0.16	0.49
"Measles (wMev)"	"Treponema palladium (tmpa)"	1.01	0.46	2.38	0.99	1
"Measles (wMev)"	"Wuchereria bancrofti (wb123)"	1.32	0.89	1.97	0.17	0.5
"Measles (wMev)"	"Diptheria (Dip tox)"	1.35	1	1.82	0.05	0.27
"Measles (wMev)"	"Rubella (wRuv)"	4.67	3.37	6.51	0	0
"Measles (wMev)"	"Tetanus (Tet tox)"	1.33	1.02	1.75	0.04	0.24
"Rubella (wRuv)"	"SARS-CoV-2(sars2np)"	3.68	1.01	21.01	0.09	0.35
"Rubella (wRuv)"	"SARS-CoV-2(sars2rbd)"	0.45	0.08	2.49	0.36	0.7
"Rubella (wRuv)"	"Cryptosporidium parvum(cp17)"	0.83	0.52	1.33	0.45	0.77
"Rubella (wRuv)"	"Cryptosporidium parvum(cp23)"	0.71	0.48	1.03	0.08	0.32
"Rubella (wRuv)"	"Giardia lamblia(vsp3)"	1.16	0.76	1.81	0.49	0.79
"Rubella (wRuv)"	"Giardia lamblia(vsp5)"	0.51	0.32	0.81	0	0.06
"Rubella (wRuv)"	"P. falciparum(csp)"	1.04	0.69	1.57	0.86	0.96
"Rubella (wRuv)"	"P. falciparum(etramp5ag1)"	1.14	0.71	1.85	0.58	0.86
"Rubella (wRuv)"	"P. falciparum(gexp18)"	0.65	0.31	1.44	0.27	0.62
"Rubella (wRuv)"	"P. falciparum(glurpr2)"	1.03	0.69	1.54	0.87	0.96
"Rubella (wRuv)"	"P. falciparum(pfama1)"	0.73	0.48	1.11	0.14	0.45
"Rubella (wRuv)"	"P. falciparum(pfmsp119)"	1.41	0.98	2.04	0.07	0.3
"Rubella (wRuv)"	"P. falciparum(rh42)"	1.67	0.93	3.11	0.1	0.36
"Rubella (wRuv)"	"P. malariae(pmmsp119)"	1.18	0.66	2.18	0.58	0.86
"Rubella (wRuv)"	"P. ovale(pomsp119)"	0.75	0.37	1.56	0.42	0.75
"Rubella (wRuv)"	"P. vivax(pvdbprii)"	1.52	0.44	5.74	0.52	0.81
"Rubella (wRuv)"	"P. vivax(pvmsp119)"	1.84	0.52	8.21	0.38	0.72
"Rubella (wRuv)"	"P. vivax(pvrbp2b)"	0.5	0.14	1.72	0.28	0.62
"Rubella (wRuv)"	"Brugia malayi (bm14)"	0.71	0.35	1.5	0.35	0.7
"Rubella (wRuv)"	"Brugia malayi (bm33)"	0.83	0.55	1.25	0.37	0.72
"Rubella (wRuv)"	"Chlamydia trachomatis (ct694)"	0.73	0.45	1.21	0.22	0.57
"Rubella (wRuv)"	"Chlamydia trachomatis (pgp3)"	2.78	1.6	4.93	0	0.01
"Rubella (wRuv)"	"Onchocerca volvulus (ov16)"	0.32	0.09	1.24	0.08	0.34
"Rubella (wRuv)"	"Schistosoma mansoni (sea)"	1.29	0.88	1.87	0.19	0.52
"Rubella (wRuv)"	"Schistosoma mansoni (sm25)"	1.21	0.79	1.87	0.37	0.72

"Rubella (wRuv)"	"Strongyloides stercoralis (nie)"	0.94	0.55	1.64	0.81	0.96
"Rubella (wRuv)"	"Taenia solium(es33)"	0.89	0.52	1.55	0.67	0.9
"Rubella (wRuv)"	"Taenia solium(t24h)"	1.4	0.78	2.59	0.27	0.62
"Rubella (wRuv)"	"Treponema palladium (rp17)"	0.72	0.42	1.24	0.22	0.57
"Rubella (wRuv)"	"Treponema palladium (tmpa)"	4.35	1.26	21.62	0.04	0.24
"Rubella (wRuv)"	"Wuchereria bancrofti (wb123)"	0.69	0.42	1.13	0.14	0.45
"Rubella (wRuv)"	"Diptheria (Dip tox)"	1.3	0.91	1.86	0.15	0.46
"Rubella (wRuv)"	"Measles (wMev)"	4.53	3.27	6.33	0	0
"Rubella (wRuv)"	"Tetanus (Tet tox)"	1.74	1.26	2.43	0	0.02
"Tetanus (Tet tox)"	"SARS-CoV-2(sars2np)"	2.12	1.13	4.16	0.02	0.19
"Tetanus (Tet tox)"	"SARS-CoV-2(sars2rbd)"	0.55	0.2	1.5	0.24	0.59
"Tetanus (Tet tox)"	"Cryptosporidium parvum(cp17)"	1.02	0.71	1.48	0.9	0.98
"Tetanus (Tet tox)"	"Cryptosporidium parvum(cp23)"	1.18	0.89	1.57	0.26	0.61
"Tetanus (Tet tox)"	"Giardia lamblia(vsp3)"	0.95	0.7	1.29	0.74	0.93
"Tetanus (Tet tox)"	"Giardia lamblia(vsp5)"	1.29	0.92	1.81	0.14	0.45
"Tetanus (Tet tox)"	"P. falciparum(csp)"	0.97	0.71	1.3	0.82	0.96
"Tetanus (Tet tox)"	"P. falciparum(etramp5ag1)"	0.94	0.68	1.3	0.69	0.9
"Tetanus (Tet tox)"	"P. falciparum(gexp18)"	1.21	0.71	2.05	0.49	0.79
"Tetanus (Tet tox)"	"P. falciparum(glurpr2)"	1.01	0.73	1.39	0.96	0.99
"Tetanus (Tet tox)"	"P. falciparum(pfama1)"	0.81	0.59	1.12	0.21	0.55
"Tetanus (Tet tox)"	"P. falciparum(pfmsp119)"	1.24	0.94	1.63	0.13	0.44
"Tetanus (Tet tox)"	"P. falciparum(rh42)"	0.65	0.44	0.96	0.03	0.22
"Tetanus (Tet tox)"	"P. malariae(pmmsp119)"	1.03	0.7	1.51	0.89	0.97
"Tetanus (Tet tox)"	"P. ovale(pomsp119)"	1.63	1	2.69	0.05	0.27
"Tetanus (Tet tox)"	"P. vivax(pvdbprii)"	1.66	0.67	4.29	0.28	0.63
"Tetanus (Tet tox)"	"P. vivax(pvmsp119)"	1.34	0.66	2.81	0.42	0.75
"Tetanus (Tet tox)"	"P. vivax(pvrbp2b)"	0.63	0.25	1.54	0.32	0.66
"Tetanus (Tet tox)"	"Brugia malayi (bm14)"	1.35	0.85	2.15	0.21	0.55
"Tetanus (Tet tox)"	"Brugia malayi (bm33)"	0.71	0.52	0.97	0.03	0.21
"Tetanus (Tet tox)"	"Chlamydia trachomatis (ct694)"	1.14	0.8	1.62	0.47	0.78
"Tetanus (Tet tox)"	"Chlamydia trachomatis (pgp3)"	1.47	1.03	2.1	0.03	0.23
"Tetanus (Tet tox)"	"Onchocerca volvulus (ov16)"	0.42	0.15	1.12	0.09	0.35
"Tetanus (Tet tox)"	"Schistosoma mansoni (sea)"	1.03	0.77	1.38	0.83	0.96
"Tetanus (Tet tox)"	"Schistosoma mansoni (sm25)"	0.95	0.69	1.32	0.78	0.95
"Tetanus (Tet tox)"	"Strongyloides stercoralis (nie)"	0.95	0.67	1.36	0.79	0.95
"Tetanus (Tet tox)"	"Taenia solium(es33)"	1.05	0.71	1.54	0.81	0.96
"Tetanus (Tet tox)"	"Taenia solium(t24h)"	1.01	0.69	1.48	0.97	0.99
"Tetanus (Tet tox)"	"Treponema palladium (rp17)"	0.88	0.61	1.27	0.5	0.79
"Tetanus (Tet tox)"	"Treponema palladium (tmpa)"	0.95	0.49	1.82	0.88	0.96
"Tetanus (Tet tox)"	"Wuchereria bancrofti (wb123)"	0.74	0.52	1.04	0.08	0.33
"Tetanus (Tet tox)"	"Diptheria (Dip tox)"	1.78	1.35	2.36	0	0
"Tetanus (Tet tox)"	"Measles (wMev)"	1.34	1.02	1.75	0.03	0.23
"Tetanus (Tet tox)"	"Rubella (wRuv)"	1.8	1.31	2.49	0	0.01

Adjusted odds ratios of seropositivity to outcome antigens as compared to regressor antigens among all 1,292 participants. All odds ratios are adjusted for seropositivity to all other antigens, age in years, Glutathione-S-transferase (GST) reactivity and uninfected Vero cell lysate reactivity and urban or rural

environment. P. is short for *plasmodium*. Adjusted p-values considered statistical significant at ($p<0.05$ for 2-sided hypothesis test, values adjusted for multiple hypothesis testing).

Supplementary References

1. McCaffrey DF, Ridgeway G, Morral AR. Propensity score estimation with boosted regression for evaluating causal effects in observational studies. *Psychol Methods* 2004; **9**(4): 403-25.
2. Ridgeway G, Kovalchik SA, Griffin BA, Kabeto MU. Propensity Score Analysis with Survey Weighted Data. *J Causal Inference* 2015; **3**(2): 237-49.
3. Potter FZ, Y. Methods and Issues in Trimming Extreme Weights in Sample Surveys. Joint Statistical Meetings 2015 Survey Research Methods Section; 2015; Seattle, Washington: American Statistical Association; 2015. p. 2707-19.
4. Hens N SZ, Aerts M, Faes C, Van Damme P, Beutels P. Modeling Infectious Disease Parameters Based on Serological and Social Contact data. 1 ed. New York, NY: Springer; 2012.
5. Gelman AR, D.B. Inference from iterative simulation using multiple sequences. *Statistical Science* 1992; (7): 457-72.
6. Benjamini Y HY. Controlling the false discovery rate: a practical and powerful approach to multiple testing. *Journal of the Royal Statistical Society, Series B (Methodological)* 1995; **57**: 289-300.
7. Rahman MH, Macicame I, Wilson E, Nhachungue S, Amouzou A. Verbal and Social Autopsy of Adult Deaths and Adult Care-Seeking Pattern in Mozambique, 2019-2020. *Am J Trop Med Hyg* 2023; **108**(5_Suppl): 17-28.
8. Chandrashekar R, Curtis KC, Ramzy RM, Liftis F, Li BW, Weil GJ. Molecular cloning of Brugia malayi antigens for diagnosis of lymphatic filariasis. *Mol Biochem Parasitol* 1994; **64**(2): 261-71.
9. Hamlin KL, Moss DM, Priest JW, et al. Longitudinal monitoring of the development of antifilarial antibodies and acquisition of Wuchereria bancrofti in a highly endemic area of Haiti. *PLoS Negl Trop Dis* 2012; **6**(12): e1941.
10. Dissanayake S, Xu M, Nkenfou C, Piessens WF. Molecular cloning and serological characterization of a Brugia malayi pepsin inhibitor homolog. *Mol Biochem Parasitol* 1993; **62**(1): 143-6.
11. Moss DM, Priest JW, Boyd A, et al. Multiplex bead assay for serum samples from children in Haiti enrolled in a drug study for the treatment of lymphatic filariasis. *Am J Trop Med Hyg* 2011; **85**(2): 229-37.
12. Wang J, Zhang Y, Lu C, Lei L, Yu P, Zhong G. A genome-wide profiling of the humoral immune response to Chlamydia trachomatis infection reveals vaccine candidate antigens expressed in humans. *J Immunol* 2010; **185**(3): 1670-80.
13. Goodhew EB, Priest JW, Moss DM, et al. CT694 and pgp3 as serological tools for monitoring trachoma programs. *PLoS Negl Trop Dis* 2012; **6**(11): e1873.
14. Priest JW, Kwon JP, Arrowood MJ, Lammie PJ. Cloning of the immunodominant 17-kDa antigen from Cryptosporidium parvum. *Mol Biochem Parasitol* 2000; **106**(2): 261-71.
15. Moss DM, Montgomery JM, Newland SV, Priest JW, Lammie PJ. Detection of cryptosporidium antibodies in sera and oral fluids using multiplex bead assay. *J Parasitol* 2004; **90**(2): 397-404.
16. Priest JW, Moss DM. Measuring Cryptosporidium Serologic Responses by Multiplex Bead Assay. *Methods Mol Biol* 2020; **2052**: 61-85.

17. Priest JW, Kwon JP, Moss DM, et al. Detection by enzyme immunoassay of serum immunoglobulin G antibodies that recognize specific Cryptosporidium parvum antigens. *J Clin Microbiol* 1999; **37**(5): 1385-92.
18. Stickings P, Rigsby P, Coombes L, et al. Calibration and commutability assessment of the 1st International Standard for Diphtheria Antitoxin Human. *Biologicals* 2013; **41**(6): 384-92.
19. Scobie HM, Khetsuriani N, Efstratiou A, Priest JW. Validation of a diphtheria toxoid multiplex bead assay for serosurveys. *Diagn Microbiol Infect Dis* 2021; **100**(3): 115371.
20. Smith DB, Johnson KS. Single-step purification of polypeptides expressed in Escherichia coli as fusions with glutathione S-transferase. *Gene* 1988; **67**(1): 31-40.
21. Priest JW, Moss DM, Visvesvara GS, Jones CC, Li A, Isaac-Renton JL. Multiplex assay detection of immunoglobulin G antibodies that recognize Giardia intestinalis and Cryptosporidium parvum antigens. *Clin Vaccine Immunol* 2010; **17**(11): 1695-707.
22. Coughlin MM, Matson Z, Sowers SB, et al. Development of a Measles and Rubella Multiplex Bead Serological Assay for Assessing Population Immunity. *J Clin Microbiol* 2021; **59**(6).
23. Smits GP, van Gageldonk PG, Schouls LM, van der Klis FR, Berbers GA. Development of a bead-based multiplex immunoassay for simultaneous quantitative detection of IgG serum antibodies against measles, mumps, rubella, and varicella-zoster virus. *Clin Vaccine Immunol* 2012; **19**(3): 396-400.
24. Feeser KR, Cama V, Priest JW, et al. Characterizing Reactivity to Onchocerca volvulus Antigens in Multiplex Bead Assays. *Am J Trop Med Hyg* 2017; **97**(3): 666-72.
25. Kastenmuller K, Espinosa DA, Trager L, et al. Full-length Plasmodium falciparum circumsporozoite protein administered with long-chain poly(I.C) or the Toll-like receptor 4 agonist glucopyranosyl lipid adjuvant-stable emulsion elicits potent antibody and CD4+ T cell immunity and protection in mice. *Infect Immun* 2013; **81**(3): 789-800.
26. Wu L, Hall T, Ssewanyana I, et al. Optimisation and standardisation of a multiplex immunoassay of diverse Plasmodium falciparum antigens to assess changes in malaria transmission using sero-epidemiology. *Wellcome Open Res* 2019; **4**: 26.
27. Spielmann T, Fergusen DJ, Beck HP. etramps, a new Plasmodium falciparum gene family coding for developmentally regulated and highly charged membrane proteins located at the parasite-host cell interface. *Mol Biol Cell* 2003; **14**(4): 1529-44.
28. Druetz T, van den Hoogen L, Stresman G, et al. Etramp5 as a useful serological marker in children to assess the immediate effects of mass drug campaigns for malaria. *BMC Infect Dis* 2022; **22**(1): 643.
29. Wu L, Hsiang MS, Prach LM, et al. Serological evaluation of the effectiveness of reactive focal mass drug administration and reactive vector control to reduce malaria transmission in Zambezi Region, Namibia: Results from a secondary analysis of a cluster randomised trial. *EClinicalMedicine* 2022; **44**: 101272.
30. Wu L, Mwesigwa J, Affara M, et al. Antibody responses to a suite of novel serological markers for malaria surveillance demonstrate strong correlation with clinical and parasitological infection across seasons and transmission settings in The Gambia. *BMC Med* 2020; **18**(1): 304.
31. Helb DA, Tetteh KK, Felgner PL, et al. Novel serologic biomarkers provide accurate estimates of recent Plasmodium falciparum exposure for individuals and communities. *Proc Natl Acad Sci U S A* 2015; **112**(32): E4438-47.
32. Theisen M, Vuust J, Gottschau A, Jepsen S, Hogh B. Antigenicity and immunogenicity of recombinant glutamate-rich protein of Plasmodium falciparum expressed in Escherichia coli. *Clin Diagn Lab Immunol* 1995; **2**(1): 30-4.

33. Collins CR, Withers-Martinez C, Bentley GA, Batchelor AH, Thomas AW, Blackman MJ. Fine mapping of an epitope recognized by an invasion-inhibitory monoclonal antibody on the malaria vaccine candidate apical membrane antigen 1. *J Biol Chem* 2007; **282**(10): 7431-41.
34. Rogier E, Wiegand R, Moss D, et al. Multiple comparisons analysis of serological data from an area of low Plasmodium falciparum transmission. *Malar J* 2015; **14**: 436.
35. Polley SD, Tetteh KK, Cavanagh DR, et al. Repeat sequences in block 2 of Plasmodium falciparum merozoite surface protein 1 are targets of antibodies associated with protection from malaria. *Infect Immun* 2003; **71**(4): 1833-42.
36. Burghaus PA, Holder AA. Expression of the 19-kilodalton carboxy-terminal fragment of the Plasmodium falciparum merozoite surface protein-1 in Escherichia coli as a correctly folded protein. *Mol Biochem Parasitol* 1994; **64**(1): 165-9.
37. Blackman MJ, Ling IT, Nicholls SC, Holder AA. Proteolytic processing of the Plasmodium falciparum merozoite surface protein-1 produces a membrane-bound fragment containing two epidermal growth factor-like domains. *Mol Biochem Parasitol* 1991; **49**(1): 29-33.
38. Egan A, Waterfall M, Pinder M, Holder A, Riley E. Characterization of human T- and B-cell epitopes in the C terminus of Plasmodium falciparum merozoite surface protein 1: evidence for poor T-cell recognition of polypeptides with numerous disulfide bonds. *Infect Immun* 1997; **65**(8): 3024-31.
39. Assefa A, Ali Ahmed A, Deressa W, et al. Multiplex serology demonstrate cumulative prevalence and spatial distribution of malaria in Ethiopia. *Malar J* 2019; **18**(1): 246.
40. Reiling L, Richards JS, Fowkes FJ, et al. The Plasmodium falciparum erythrocyte invasion ligand Pfrh4 as a target of functional and protective human antibodies against malaria. *PLoS One* 2012; **7**(9): e45253.
41. Beeson JG, Drew DR, Boyle MJ, Feng G, Fowkes FJ, Richards JS. Merozoite surface proteins in red blood cell invasion, immunity and vaccines against malaria. *FEMS Microbiol Rev* 2016; **40**(3): 343-72.
42. Priest JW, Plucinski MM, Huber CS, et al. Specificity of the IgG antibody response to Plasmodium falciparum, Plasmodium vivax, Plasmodium malariae, and Plasmodium ovale MSP1(19) subunit proteins in multiplexed serologic assays. *Malar J* 2018; **17**(1): 417.
43. Oviedo A, Herman C, Knipes A, et al. Spatial cluster analysis of Plasmodium vivax and P. malariae exposure using serological data among Haitian school children sampled between 2014 and 2016. *PLoS Negl Trop Dis* 2022; **16**(1): e0010049.
44. Byrne I, Cramer E, Nelli L, et al. Characterizing the spatial distribution of multiple malaria diagnostic endpoints in a low-transmission setting in Lao PDR. *Front Med (Lausanne)* 2022; **9**: 929366.
45. King CL, Michon P, Shakri AR, et al. Naturally acquired Duffy-binding protein-specific binding inhibitory antibodies confer protection from blood-stage Plasmodium vivax infection. *Proc Natl Acad Sci U S A* 2008; **105**(24): 8363-8.
46. Grimberg BT, Udomsangpetch R, Xainli J, et al. Plasmodium vivax invasion of human erythrocytes inhibited by antibodies directed against the Duffy binding protein. *PLoS Med* 2007; **4**(12): e337.
47. Hietanen J, Chim-Ong A, Chiramanewong T, et al. Gene Models, Expression Repertoire, and Immune Response of Plasmodium vivax Reticulocyte Binding Proteins. *Infect Immun* 2015; **84**(3): 677-85.
48. Franca CT, He WQ, Gruszczyk J, et al. Plasmodium vivax Reticulocyte Binding Proteins Are Key Targets of Naturally Acquired Immunity in Young Papua New Guinean Children. *PLoS Negl Trop Dis* 2016; **10**(9): e0005014.
49. Noval MG, Kaczmarek ME, Koide A, et al. Antibody isotype diversity against SARS-CoV-2 is associated with differential serum neutralization capacities. *Sci Rep* 2021; **11**(1): 5538.

50. Lawal BJ, Gallagher KE, Kitonsa J, et al. Prevalence of immunoglobulin G and M to SARS-CoV-2 and other human coronaviruses in The Democratic Republic of Congo, Sierra Leone, and Uganda: A longitudinal study. *Int J Infect Dis* 2023; **131**: 183-92.
51. Carter CE, Colley DG. An electrophoretic analysis of Schistosoma mansoni soluble egg antigen preparation. *J Parasitol* 1978; **64**(3): 285-90.
52. Won KY, Kanyi HM, Mwende FM, et al. Multiplex Serologic Assessment of Schistosomiasis in Western Kenya: Antibody Responses in Preschool Aged Children as a Measure of Reduced Transmission. *Am J Trop Med Hyg* 2017; **96**(6): 1460-7.
53. Ali PO, Jeffs SA, Meadows HM, et al. Structure of Sm25, an antigenic integral membrane glycoprotein of adult Schistosoma mansoni. *Mol Biochem Parasitol* 1991; **45**(2): 215-22.
54. Tsang VC, Hancock K, Kelly MA, Wilson BC, Maddison SE. Schistosoma mansoni adult microsomal antigens, a serologic reagent. II. Specificity of antibody responses to the S. mansoni microsomal antigen (MAMA). *J Immunol* 1983; **130**(3): 1366-70.
55. Ravi V, Ramachandran S, Thompson RW, Andersen JF, Neva FA. Characterization of a recombinant immunodiagnostic antigen (NIE) from Strongyloides stercoralis L3-stage larvae. *Mol Biochem Parasitol* 2002; **125**(1-2): 73-81.
56. Rascoe LN, Price C, Shin SH, McAuliffe I, Priest JW, Handali S. Development of Ss-NIE-1 recombinant antigen based assays for immunodiagnosis of strongyloidiasis. *PLoS Negl Trop Dis* 2015; **9**(4): e0003694.
57. Levine MZ, Calderon JC, Wilkins PP, et al. Characterization, cloning, and expression of two diagnostic antigens for Taenia solium tapeworm infection. *J Parasitol* 2004; **90**(3): 631-8.
58. Hancock K, Pattabhi S, Whitfield FW, et al. Characterization and cloning of T24, a Taenia solium antigen diagnostic for cysticercosis. *Mol Biochem Parasitol* 2006; **147**(1): 109-17.
59. Hernandez-Gonzalez A, Noh J, Perteguer MJ, Garate T, Handali S. Comparison of T24H-his, GST-T24H and GST-Ts8B2 recombinant antigens in western blot, ELISA and multiplex bead-based assay for diagnosis of neurocysticercosis. *Parasit Vectors* 2017; **10**(1): 237.
60. Scobie HM, Mao B, Buth S, et al. Tetanus Immunity among Women Aged 15 to 39 Years in Cambodia: a National Population-Based Serosurvey, 2012. *Clin Vaccine Immunol* 2016; **23**(7): 546-54.
61. Sesardic D, Wong MY, Gaines Das RE, Corbel MJ. The First International Standard for Antitetanus Immunoglobulin, Human; pharmaceutical evaluation and international collaborative study. *Biologicals* 1993; **21**(1): 67-75.
62. Cooley GM, Mitja O, Goodhew B, et al. Evaluation of Multiplex-Based Antibody Testing for Use in Large-Scale Surveillance for Yaws: a Comparative Study. *J Clin Microbiol* 2016; **54**(5): 1321-5.
63. Ijsselmuiden OE, Schouls LM, Stolz E, et al. Sensitivity and specificity of an enzyme-linked immunosorbent assay using the recombinant DNA-derived Treponema pallidum protein TmpA for serodiagnosis of syphilis and the potential use of TmpA for assessing the effect of antibiotic therapy. *J Clin Microbiol* 1989; **27**(1): 152-7.
64. Kubofcik J, Fink DL, Nutman TB. Identification of Wb123 as an early and specific marker of Wuchereria bancrofti infection. *PLoS Negl Trop Dis* 2012; **6**(12): e1930.
65. Steel C, Golden A, Kubofcik J, et al. Rapid Wuchereria bancrofti-specific antigen Wb123-based IgG4 immunoassays as tools for surveillance following mass drug administration programs on lymphatic filariasis. *Clin Vaccine Immunol* 2013; **20**(8): 1155-61.
66. Priest JW, Jenks MH, Moss DM, et al. Integration of Multiplex Bead Assays for Parasitic Diseases into a National, Population-Based Serosurvey of Women 15-39 Years of Age in Cambodia. *PLoS Negl Trop Dis* 2016; **10**(5): e0004699.
