# Supplementary material for: Multiplex bead assays enable integrated serological surveillance and reveal cross-pathogen vulnerabilities in Zambezia Province, Mozambique
Source: Nat Commun. 2025 Aug 26;16:7946. doi: 10.1038/s41467-025-62305-9 (PMC12381283; doi:10.1038/s41467-025-62305-9)
Supplement: Supplementary file 3 — Reporting Summary [file 41467_2025_62305_MOESM3_ESM.pdf]

nature portfolio

Corresponding author(s): Andrea Carcelen
Last updated by author(s): Jul 2, 2025

# Reporting Summary

Nature Portfolio wishes to improve the reproducibility of the work that we publish. This form provides structure for consistency and transparency in reporting. For further information on Nature Portfolio policies, see our Editorial Policies and the Editorial Policy Checklist.

## Statistics

For all statistical analyses, confirm that the following items are present in the figure legend, table legend, main text, or Methods section.

| n/a | Confirmed | |
|---|---|---|
| ☐ | ☒ | The exact sample size (*n*) for each experimental group/condition, given as a discrete number and unit of measurement |
| ☐ | ☒ | A statement on whether measurements were taken from distinct samples or whether the same sample was measured repeatedly |
| ☐ | ☒ | The statistical test(s) used AND whether they are one- or two-sided<br>*Only common tests should be described solely by name; describe more complex techniques in the Methods section.* |
| ☐ | ☒ | A description of all covariates tested |
| ☐ | ☒ | A description of any assumptions or corrections, such as tests of normality and adjustment for multiple comparisons |
| ☐ | ☒ | A full description of the statistical parameters including central tendency (e.g. means) or other basic estimates (e.g. regression coefficient) AND variation (e.g. standard deviation) or associated estimates of uncertainty (e.g. confidence intervals) |
| ☐ | ☒ | For null hypothesis testing, the test statistic (e.g. *F*, *t*, *r*) with confidence intervals, effect sizes, degrees of freedom and *P* value noted<br>*Give P values as exact values whenever suitable.* |
| ☐ | ☒ | For Bayesian analysis, information on the choice of priors and Markov chain Monte Carlo settings |
| ☒ | ☐ | For hierarchical and complex designs, identification of the appropriate level for tests and full reporting of outcomes |
| ☐ | ☒ | Estimates of effect sizes (e.g. Cohen's *d*, Pearson's *r*), indicating how they were calculated |

*Our web collection on statistics for biologists contains articles on many of the points above.*

## Software and code

Policy information about availability of computer code

| | |
|---|---|
| Data collection | Data were collected electronically on tablets using ODK software. |
| Data analysis | Analyses were performed using STATA (version 14.2), SAS (version 9.4), and R (version 4.2.2). R code for this analysis is available at https://github.com/sberube3/COMSA_moz_analysis.git. |

For manuscripts utilizing custom algorithms or software that are central to the research but not yet described in published literature, software must be made available to editors and reviewers. We strongly encourage code deposition in a community repository (e.g. GitHub). See the Nature Portfolio guidelines for submitting code & software for further information.

## Data

Policy information about availability of data

All manuscripts must include a data availability statement. This statement should provide the following information, where applicable:

- Accession codes, unique identifiers, or web links for publicly available datasets
- A description of any restrictions on data availability
- For clinical datasets or third party data, please ensure that the statement adheres to our policy

Raw individual-level data that underlie the results reported in this article (text, tables, figures, and supplementary material) and the study protocol are protected and are not available publicly due to data privacy laws. These data are available after de-identification upon request to the National Institute of Health of Mozambique via the corresponding author beginning one year after publication and ending three years after publication. Researchers must submit a proposal to be approved by authorities in Mozambique using an institutional data request form. The processed data denoting overall seroprevalence, seroprevalence by age, sex,

geography and cluster are provided in Supplementary Information.

## Research involving human participants, their data, or biological material

Policy information about studies with human participants or human data. See also policy information about sex, gender (identity/presentation), and sexual orientation and race, ethnicity and racism.

| | |
|---|---|
| Reporting on sex and gender | Sex was based on self-report in the sociodemographic questionnaire. Analyses included seroprevalence by sex to identify any differences. |
| Reporting on race, ethnicity, or other socially relevant groupings | Race and ethnicity were not included in this analysis. A wealth index was calculated, as described in the supplemental methods. |
| Population characteristics | Age, sex, education, occupation, and socioeconomic status were self-reported. Age was reported based on date of birth or estimated age in years (unless under 2, when reported in months). Clusters were defined and cluster-level designation of rural-urban was based on the statistical office designation. Population characteristics are defined in Table 2. |
| Recruitment | The Mozambique Community Mortality Surveillance for Action (COMSA) platform was used as the sampling frame to select eligible participants. The DBS serological survey team confirmed the eligibility of the participant upon arrival at the participant's house and assessed eligibility criteria (inclusion/exclusion criteria). If the participant is not eligible or consent/ assent/parental permission is not provided, the study teams ended the visit.<br>Participants in the selected households were informed of the opportunity to participate in a research study that aims to estimate the proportion of the community protected against or exposed to malaria, HIV, vaccine preventable diseases and other infectious diseases. Participation in the serological survey was voluntary. Each eligible individual or their parent/ guardian was asked to sign a consent, assent and/or a parental permission form. The recruitment process was managed by the COMSA study team from the National Institute of Statistics (INE). The COMSA study team appointed teams consisting of trained data collectors, a supervisor, and at least one staff member with finger prick blood collection experience to enroll participants and carry out study procedures.<br>There are potential self-selection biases in terms of who agreed to participate in the study and provide blood specimens. We accounted for non-response in the weighting, as detailed in supplementary methods. If participants who were not included were more likely to have been exposed, seroprevalence will be underestimated. However, if participants who were not included were less likely to be vaccinated, seroprevalence would be overestimated. We cannot say the direction of the bias. |
| Ethics oversight | The study protocol was approved by the National Bioethics Committee for Health of Mozambique and the Johns Hopkins Bloomberg School of Public Health Institutional Review Board. Written informed consent for each participant, parental permission for children younger than 18 years of age, and written assent for children 12–17 years of age was obtained. |

Note that full information on the approval of the study protocol must also be provided in the manuscript.

# Field-specific reporting

Please select the one below that is the best fit for your research. If you are not sure, read the appropriate sections before making your selection.

- [x] Life sciences
- [ ] Behavioural & social sciences
- [ ] Ecological, evolutionary & environmental sciences

For a reference copy of the document with all sections, see nature.com/documents/nr-reporting-summary-flat.pdf

# Life sciences study design

All studies must disclose on these points even when the disclosure is negative.

| | |
|---|---|
| Sample size | Sample size was first computed to estimate malaria seroprevalence with +8% precision and then estimated to assess whether the proposed sample size is sufficient to measure the VPD serology outcomes. Specifically, for the malaria calculations, the sample size is sufficient to estimate malaria seroprevalence within 8% assuming a conservative 50% estimate of malaria prevalence (based on recent IMASIDA survey in the area), 5% two-sided alpha, 80% power, and accounting for correlation of results within clusters. The estimated sample size is also expected to detect measles IgG seroprevalence within +6% for each age group. We assumed a non-availability rate of 25% based on a previous IMASIDA survey with blood collection with additional conservative adjustments for specimen collection refusals (18%) or collection problems(5%) (e.g., low volume, fungal contamination). This sample size was the same for each of 3 age strata (6-59 months, 5-17 years, 18-49 years) and divided evenly across 30 clusters. |
| Data exclusions | The survey enrolled 1,409 participants, with dried blood spots collected from 98% (N=1,383). After excluding specimens that did not pass quality control during laboratory testing due to fungal growth, incorrect labels, and low bead counts when run on the Magpix. The final analytic sample comprised 1,292 specimens |
| Replication | Replication was done in terms or rerunning analyses by different authors, but this was not an experimental study. |
| Randomization | No randomization was done, as this was a cross-sectional study design to estimate seroprevalence. |
| Blinding | No blinding was done, as this was a cross-sectional study design to estimate seroprevalence. |

# Reporting for specific materials, systems and methods

We require information from authors about some types of materials, experimental systems and methods used in many studies. Here, indicate whether each material, system or method listed is relevant to your study. If you are not sure if a list item applies to your research, read the appropriate section before selecting a response.

## Materials & experimental systems

| n/a | Involved in the study |
|---|---|
| ☒ | ☐ Antibodies |
| ☒ | ☐ Eukaryotic cell lines |
| ☒ | ☐ Palaeontology and archaeology |
| ☒ | ☐ Animals and other organisms |
| ☒ | ☐ Clinical data |
| ☒ | ☐ Dual use research of concern |
| ☒ | ☐ Plants |

## Methods

| n/a | Involved in the study |
|---|---|
| ☒ | ☐ ChIP-seq |
| ☒ | ☐ Flow cytometry |
| ☒ | ☐ MRI-based neuroimaging |

## Plants

Seed stocks

*Report on the source of all seed stocks or other plant material used. If applicable, state the seed stock centre and catalogue number. If plant specimens were collected from the field, describe the collection location, date and sampling procedures.*

Novel plant genotypes

*Describe the methods by which all novel plant genotypes were produced. This includes those generated by transgenic approaches, gene editing, chemical/radiation-based mutagenesis and hybridization. For transgenic lines, describe the transformation method, the number of independent lines analyzed and the generation upon which experiments were performed. For gene-edited lines, describe the editor used, the endogenous sequence targeted for editing, the targeting guide RNA sequence (if applicable) and how the editor was applied.*

Authentication

*Describe any authentication procedures for each seed stock used or novel genotype generated. Describe any experiments used to assess the effect of a mutation and, where applicable, how potential secondary effects (e.g. second site T-DNA insertions, mosiacism, off-target gene editing) were examined.*
